# Ca^2+^ dynamics in zebrafish morphogenesis

**DOI:** 10.7717/peerj.2894

**Published:** 2017-01-19

**Authors:** Yusuke Tsuruwaka, Eriko Shimada, Kenta Tsutsui, Tomohisa Ogawa

**Affiliations:** 1 Marine Bioresource Exploration Research Group, Japan Agency for Marine-Earth Science and Technology (JAMSTEC), Yokosuka, Japan; 2 Department of Animal Science, University of California, Davis, CA, United States; 3 Cellevolt, Yokohama, Japan

**Keywords:** Yellow cameleon, Intracellular calcium, Zebrafish

## Abstract

Intracellular calcium ion (Ca^2+^) signaling is heavily involved in development, as illustrated by the use of a number of Ca^2+^ indicators. However, continuous Ca^2+^ patterns during morphogenesis have not yet been studied using fluorescence resonance energy transfer to track the Ca^2+^ sensor. In the present study, we monitored Ca^2+^ levels during zebrafish morphogenesis and differentiation with yellow cameleon, YC2.12. Our results show not only clear changes in Ca^2+^ levels but also continuous Ca^2+^ patterns at 24 hpf and later periods for the first time. Serial Ca^2+^dynamics during early pharyngula period (Prim-5-20; 24–33 hpf) was successfully observed with cameleon, which have not reported anywhere yet. In fact, high Ca^2+^ level occurred concurrently with hindbrain development in segmentation and pharyngula periods. Ca^2+^ patterns in the late gastrula through segmentation periods which were obtained with cameleon, were similar to those obtained previously with other Ca^2+^sensor. Our results suggested that the use of various Ca^2+^ sensors may lead to novel findings in studies of Ca^2+^ dynamics. We hope that these results will prove valuable for further research in Ca^2+^ signaling.

## Introduction

Intracellular calcium ions (Ca^2+^) act as second messengers in organism cellular signaling pathways. Ca^2+^ is relevant to most biological phenomena, and is particularly relevant to early development (Niki et al., 1996; Berridge, Lipp & Bootman, 2000; Slusarski & Pelegri, 2007). Patterning intracellular Ca^2+^ concentration is important for the study of living organisms. Ca^2+^ has been measured using aequorin since the late 1960s, and using fluorescent proteins such as modified green fluorescent protein since the late 1990s (Shimomura, Johnson & Saiga, 1963; Miyawaki et al., 1999; Takahashi et al., 1999). To date, Ca^2+^ patterns during zebrafish development have been studied mostly using aequorin, and many patterns have been described (Créton, Speksnijder & Jaffe, 1998; Jaffe, 1999; Webb, Chan & Miller, 2013). However, to image Ca^2+^ patterns in more detail, a multifaceted analysis with a variety of chemical indicators is required. Advantage of a luminescent Ca^2+^ sensor such as aequorin is that not carrying phototoxicity due to excitation lights. On the other hand, disadvantages are (1) requirement of the substrate coelenterazine which is gradually consumed, (2) difficulty of detecting subtle signals which is weaker than the one fluorescent Ca^2+^ sensor emits, (3) occasionally unsuitable for a long-term and high-speed photography. To present, ‘continuous’ Ca^2+^ patterns such as long-term time lapse imaging in zebrafish morphogenesis after 24 hpf (hour post fertilization) have not been reported yet. Meanwhile, stable Ca^2+^ signals are expected with fluorescent Ca^2+^ sensors such as yellow cameleon YC2.12 because the sensor molecule is integrated into cells. This is advantageous in long-term measuring since Ca^2+^ sensor is synthesized *in vivo* and does not require a substrate like luminescent Ca^2+^ sensor does. Fluorescence emits stronger light than luminescence in general although requiring an excitation light, which enables us to measure real-time and to detect subtle signals.

Recently, we also reported that morphological changes which had been the consequences of *wwox* gene down regulation by morpholino injection brought about dramatic transition in Ca^2+^ signaling (Tsuruwaka, Konishi & Shimada, 2015). To date, with cameleon consecutive Ca^2+^ dynamics of zebrafish gastrulation was reported (Tsuruwaka et al., 2007). The purpose of the present study was to analyze serial Ca^2+^ patterns for long-term periods, from late gastrula to pharyngula periods, using cameleon.

## Materials and Methods

### Zebrafish and Ca^2+^ imaging

Experiments were conducted as previously described (Tsuruwaka et al., 2007; Tsuruwaka, Konishi & Shimada, 2015). Briefly, 3 nL of synthetic YC 2.12 mRNA (0.5 ng/mL) was injected into blastodiscs of each single-cell embryo. After YC2.12 had confirmed to be distributed ubiquitously in the whole embryo, FRET analyses were performed as followed. Fluorescence images were obtained using a Zeiss Axiovert 200 microscope equipped with a combination of two filters, i.e., CFP-CFP, YFP-YFP, and CFP-YFP filters (Carl Zeiss, Oberkochen, Germany). Amplification and numerical aperture of the objective lens were 5× and 0.16, respectively. An AxioCam MRc5 camera (Carl Zeiss) was used to photograph the images, and the image analysis was performed using Axiovert FRET version 4.4 software (Carl Zeiss). Fluorescence was quantified following the manufacturer’s instructions. The control experiment was performed using Ca^2+^-ATPase inhibitor thapsigargin (Wako Pure Chemical Industries, Osaka, Japan) to confirm YC2.12 would work correctly (Schneider et al., 2008; Popgeorgiev et al., 2011). The number of eggs analyzed was 300 each experiment and the experiments were performed for total 37 times. Of those, 50 eggs were employed in the control experiment. No approval was required to conduct studies on fish according to the Ministry of Education, Culture, Sports, Science and Technology, Notice No. 71 (in effect since June 1, 2006).

## Results and Discussion

### Ca^2+^ dynamics during zebrafish morphogenesis

**Figure 1:**
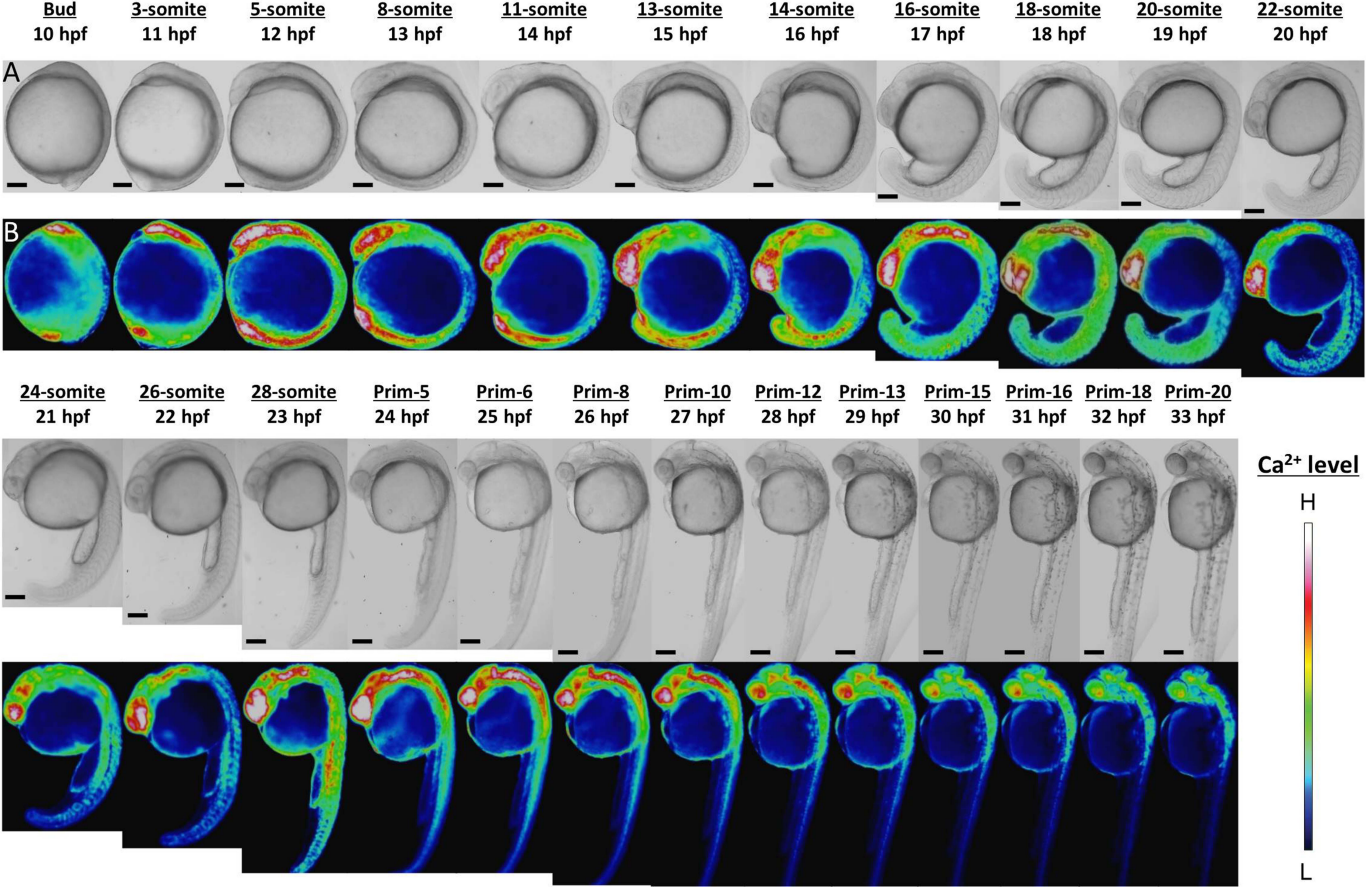
Ca^2+^ dynamics in the late gastrula, segmentation, and early pharyngula periods. (A) Bright field image; (B) color-coded image; scale bar, 200 µm (magnification, ×50). The color-coded image shows Ca^2+^ levels as white (high Ca^2+^) and blue (low Ca^2+^). Embryos used in this experiment demonstrated normal development and grew to adulthood.

Ca^2+^ patterns showed dynamic changes during zebrafish morphogenesis (Fig. 1). Since the Ca^2+^ monitoring had been well studied with aquorin by Créton, Speksnijder & Jaffe (1998), we mainly focused on novel findings here. High Ca^2+^ levels were observed in the anterior and posterior body regions from stages bud to 16-somite (10–17 hpf). In the anterior trunk, the Ca^2+^ level reached a peak at 18-somite stage, whereas in the posterior trunk the Ca^2+^ peak was shown at 28-somite stage (Fig. S1).

In the developing head, the high level of Ca^2+^ was maintained through to prim-13 stage. Notably, this high Ca^2+^ level occurred concurrently with development of rhombomere, a segment of the developing hindbrain, from stages 26-somite to prim-10 (Fig. S2). Ca^2+^ level at presumptive midbrain increased at 26-somite stage and reached maximum level at prim-5 stage. Moreover, Ca^2+^ concentration at presumptive rhombomere 2 and 4 in hindbrain started to rise from 26-somite stage and then all rhombomeres showed relatively high Ca^2+^ levels at prim-5 stage. Ca^2+^ at rhombomere 2 reached maximum level at prim-5 stage, whereas rhombomere 1, 3 and 4 did at prim-6. With focusing on the rhombomere and midbrain hindbrain boundary (MHB), it is quite interesting to consider relevance between Ca^2+^ signals and formation of neuronal network. Ca^2+^ involves with neural network in zebrafish and Ca^2+^ sensors were used for studying neuronal activity and reflexive behavior (Higashijima et al., 2003; Muto et al., 2013; Portugues et al., 2014). Serial neural circuits such as sensory neuron, intercalated neuron, motor neuron, muscle were formed within 24 hpf in zebrafish (Saint-Amant & Drapeau, 1998; Downes & Granato, 2006; Fetcho, Higashijima & McLean, 2008; Pietri et al., 2009). When those circuits become active, zebrafish acquires stimulus-response. High Ca^2+^ levels at trunk and rhombomere regions in our results coincide with the development and activation of those circuits. Especially, Mauthner cells at rhombomere 4 become active and stimulate neural circuits, which results in triggering various body movements such as escape behavior (Korn & Faber, 2005). In fact, rhombomere and MHB during brain organization closely involved with Wnt signaling pathway which controls Ca^2+^ signaling (Webb & Miller, 2000; Prakash & Wurst, 2006). Therefore, Ca^2+^ dynamics at developing head in our results suggested intimate correlation with and formation and activation of neural circuits.

In the developing tail, the Ca^2+^ level had dropped by 20-somite stage and stabilized at a low level. The patterns in Ca^2+^ levels through the late gastrula and segmentation periods (Bud-28-somite stages; 10–23 hpf) that we obtained with yellow cameleon, YC2.12, were similar to those obtained previously with aequorin (Créton, Speksnijder & Jaffe, 1998; Webb & Miller, 2000). However, we succeeded in observing Ca^2+^patterns during early pharyngula period (Prim-5-20; 24–33 hpf) which have not reported anywhere yet.

Correlations between zebrafish morphogenesis and intracellular Ca^2+^ dynamics in the late gastrula-segmentation periods have been well characterized by Webb, Miller and colleagues (Gilland et al., 1999; Webb & Miller, 2007). Their work on Ca^2+^ dynamics during somitogenesis is particularly informative (Webb & Miller, 2010; Cheung et al., 2011; Webb et al., 2012).

Our finding of increasing Ca^2+^ levels in the anterior region during the pharyngula period, when the basic body plan is complete, is consistent with Ca^2+^-related gene expression, which controls the formation of the brain and nervous system (Zhou et al., 2008; Hsu & Tseng, 2010). Moreover, patterns of CaMK-II gene expression are in agreement with our observations of Ca^2+^ patterns at 3-somite, 18-somite, prim-5 stages and later, suggesting that this gene is closely involved with Ca^2+^ dynamics (Rothschild, Lister & Tombes, 2007). Figure S3 showed the compared images between our results and the CaMK-II expressions based on Rothschild, Lister & Tombes (2007). In fact, Freisinger et al. (2008) discuss correlations between Ca^2+^ signaling pathways and zebrafish body plan formation. The present study showed that cameleon, a genetically encoded Ca^2+^ sensor, enables us to analyze Ca^2+^ dynamics clearly during development and differentiation in a zebrafish embryo. YC2.12 worked correctly as Ca^2+^ sensor in whole living embryos since treatment with Ca^2+^-ATPase inhibitor thapsigargin induced altered Ca^2+^ level (Fig. S4). The embryo shown in Fig. S4B exhibited the increased Ca^2+^ level at later stages, which was consistent with the results reported by Popgeorgiev et al. (2011) (data not shown). We have achieved in tracking the serial Ca^2+^patterns from late gastrula to early pharyngula periods for the first time. This use of a variety of Ca^2+^ sensors has led to a novel perspective in the study of Ca^2+^ dynamics.

In future, tracking whole body Ca^2+^ signaling patterns with cameleon in addition to aequorin and other sensors may provide even more detail on Ca^2+^ signaling during zebrafish development. Thus, instead of discussing whether some Ca^2+^ indicators are superior to others, we propose that the use of a variety of indicators may give better results. Further comparison of our cameleon study results with those of previous Ca^2+^ studies should lead to more insight into Ca^2+^ dynamics.

## Conclusions

Ca^2+^ patterns showed dynamic changes during zebrafish morphogenesis, as illustrated using cameleon, a genetically encoded Ca^2+^ sensor. Continuous Ca^2+^ dynamics observed with cameleon at 24 hpf and later periods was investigated for the first time. The results suggested that the use of a variety of Ca^2+^ sensors may lead to novel findings in studies of Ca^2+^ dynamics.

##  Supplemental Information

10.7717/peerj.2894/supp-1 Figure S1 Ca^2+^ dynamics of the trunk region in the late segmentation period (A) Trunk area of zebrafish embryo. (B) Ca^2+^ patterns at trunk area from 14- to 28-somite stages. Ca^2+^ level reached a peak between the 14- and 18-somite stages, fluctuated until the 26-somite stage, and then showed another peak at the 28-somite stage. Scale bar, 200 µm.

10.7717/peerj.2894/supp-2 Figure S2 Ca^2+^ dynamics of the hindbrain region in the late segmentation to early pharyngula periods (A) Developing hindbrain and schematic rhombomeres (r1-7) of zebrafish embryo. (B) Ca^2+^ patterns at rhombomere region at 26-somite to prim-10 stages. Scale bar, 200 µm.

10.7717/peerj.2894/supp-3 Figure S3 Comparison of Ca^2+^ dynamics with CaMK-II gene expression Ca^2+^ patterns (upper) coincided with CaMK-II gene expression patterns (lower) at (A) 3-somite, (B) 18-somite and (C) prim-5 stages. Schematic images of CaMK-II expressions were created based on Rothschild, Lister & Tombes, 2007. Scale bar, 200 µm.

10.7717/peerj.2894/supp-4 Figure S4 Yellow cameleon YC2.12 as Ca^2+^ sensor YC2.12 injected zebrafish embryos were treated with thapsigargin at oblong stage. (A) Ca^2+^ pattern (upper) and bright field image (lower) of the normal embryo. (B) Ca^2+^ pattern (upper) and bright field image (lower) of the embryo treated with thapsigargin 2.5 µM for 10 m. The control experiment showed that YC2.12 was working correctly as Ca^2+^ sensor. Scale bar, 200 µm.

10.7717/peerj.2894/supp-5 Data S1 Raw data

## Additional Information and Declarations

### Data Availability

The following information was supplied regarding data availability: The raw data has been supplied as Data S1.
