# Supplementary figures and images for: Ca2+ dynamics in zebrafish morphogenesis
Source: PeerJ. 2017 Jan 19;5:e2894. doi: 10.7717/peerj.2894 (PMC5251937; doi:10.7717/peerj.2894)

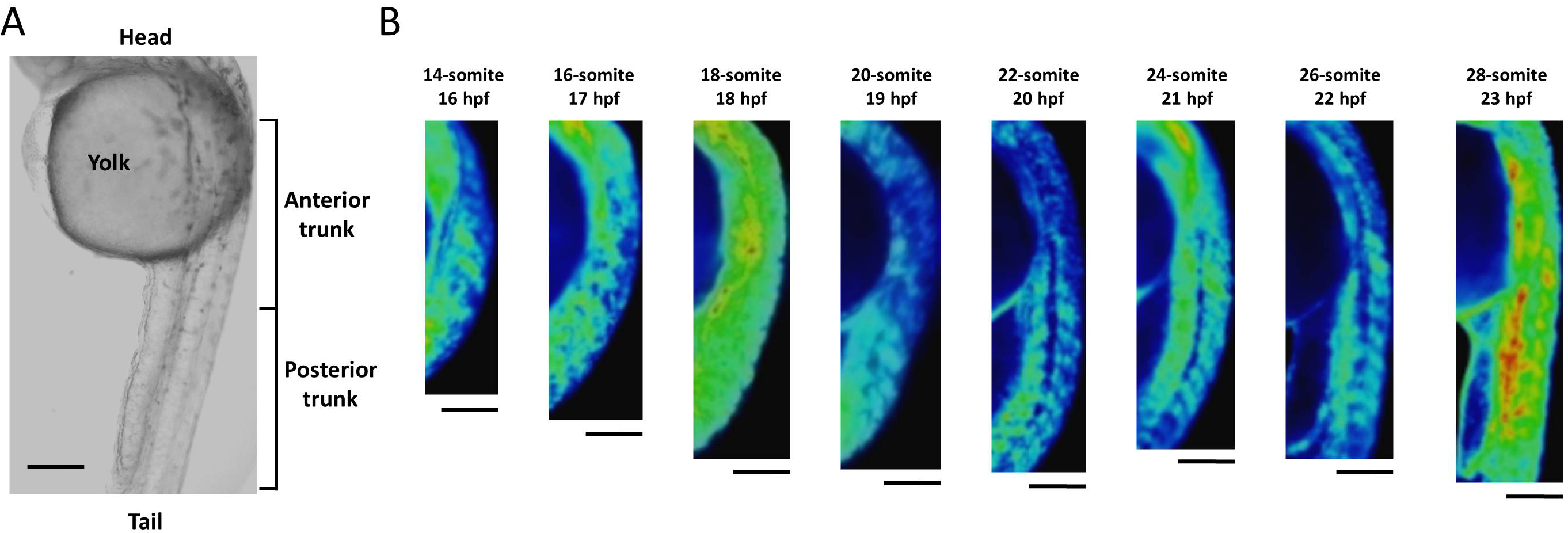

Supplement: Figure S1 — (A) Trunk area of zebrafish embryo. (B) Ca2+ patterns at trunk area from 14- to 28-somite stages. Ca2+ level reached a peak between the 14- and 18-somite stages, fluctuated until the 26-somite stage, and then showed another peak at the 28-somite stage. Scale bar, 200 µm. [file peerj-05-2894-s001.jpg]

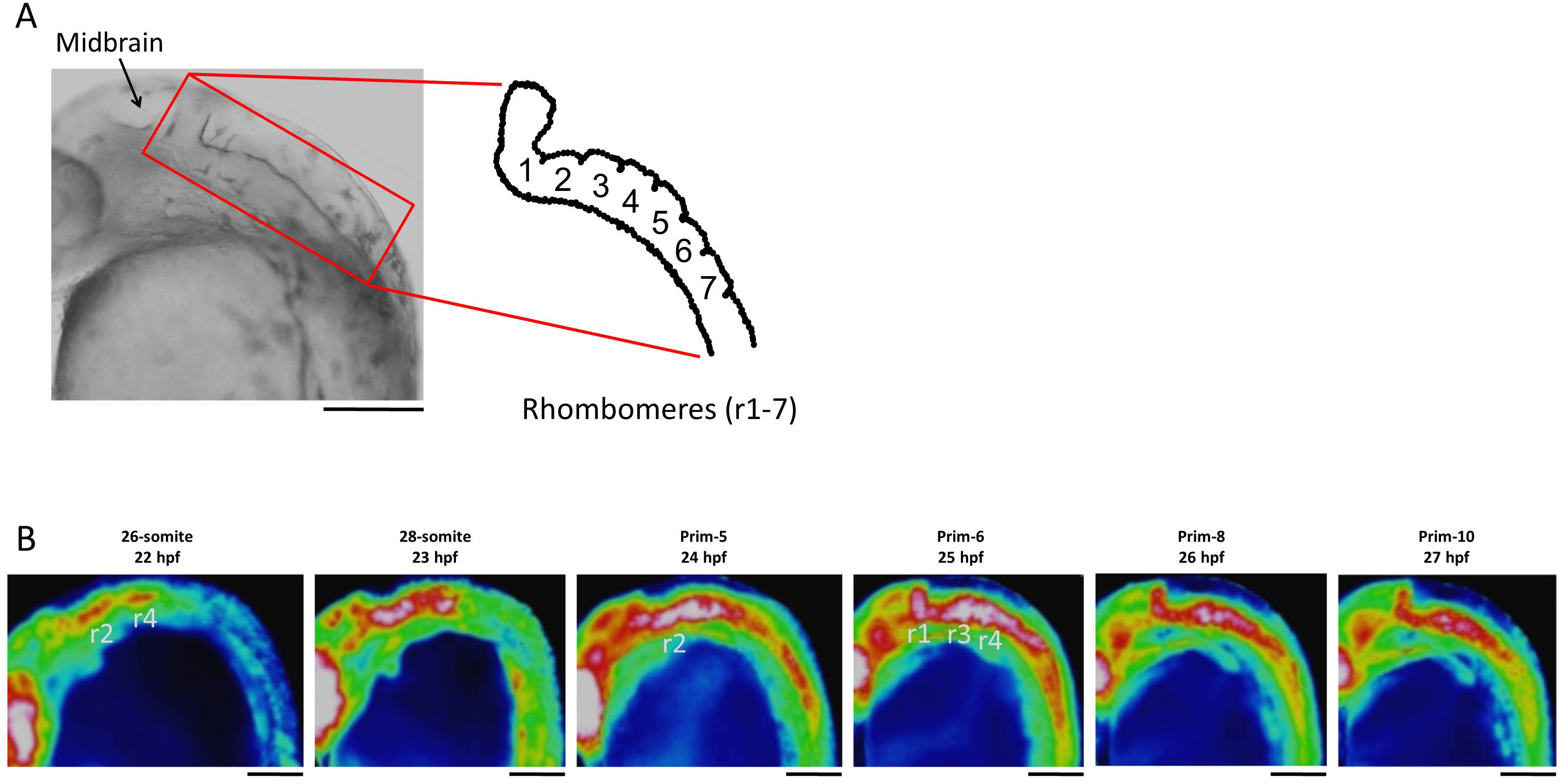

Supplement: Figure S2 — (A) Developing hindbrain and schematic rhombomeres (r1-7) of zebrafish embryo. (B) Ca2+ patterns at rhombomere region at 26-somite to prim-10 stages. Scale bar, 200 µm. [file peerj-05-2894-s002.jpg]

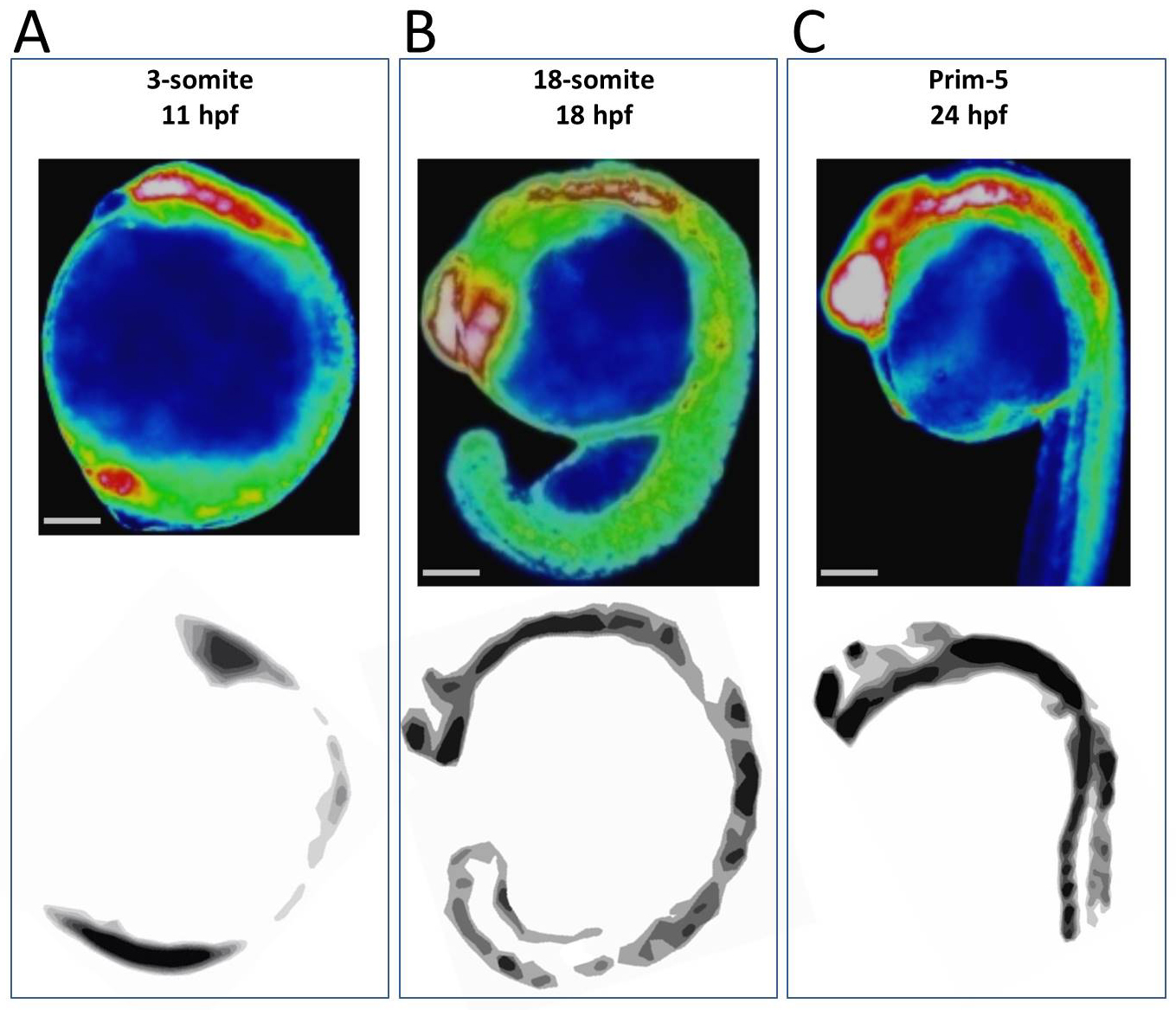

Supplement: Figure S3 — Ca2+ patterns (upper) coincided with CaMK-II gene expression patterns (lower) at (A) 3-somite, (B) 18-somite and (C) prim-5 stages. Schematic images of CaMK-II expressions were created based on Rothschild, Lister & Tombes, 2007. Scale bar, 200 µm. [file peerj-05-2894-s003.jpg]

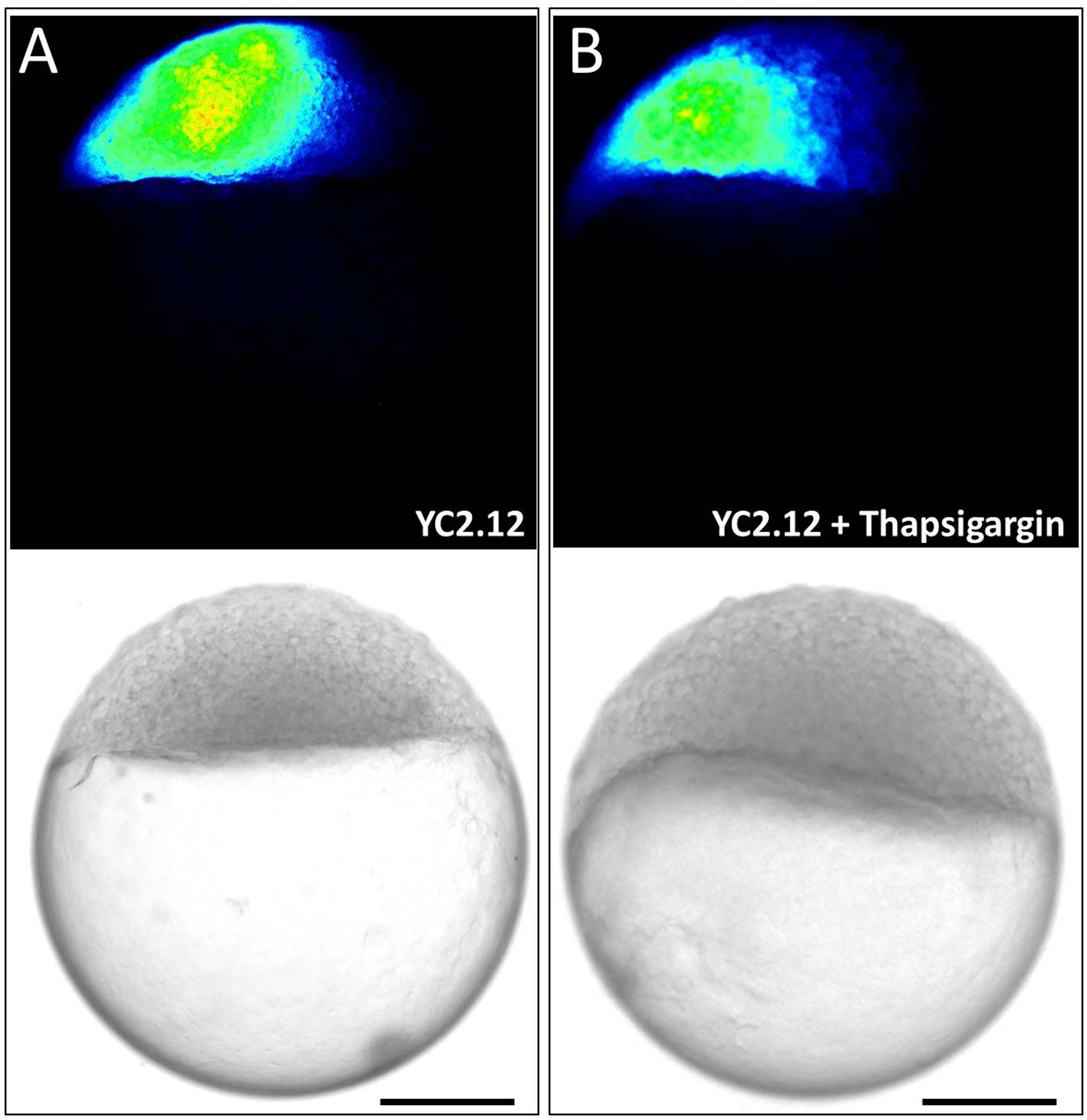

Supplement: Figure S4 — YC2.12 injected zebrafish embryos were treated with thapsigargin at oblong stage. (A) Ca2+ pattern (upper) and bright field image (lower) of the normal embryo. (B) Ca2+ pattern (upper) and bright field image (lower) of the embryo treated with thapsigargin 2.5 µM for 10 m. The control experiment showed that YC2.12 was working correctly as Ca2+ sensor. Scale bar, 200 µm. [file peerj-05-2894-s004.jpg]

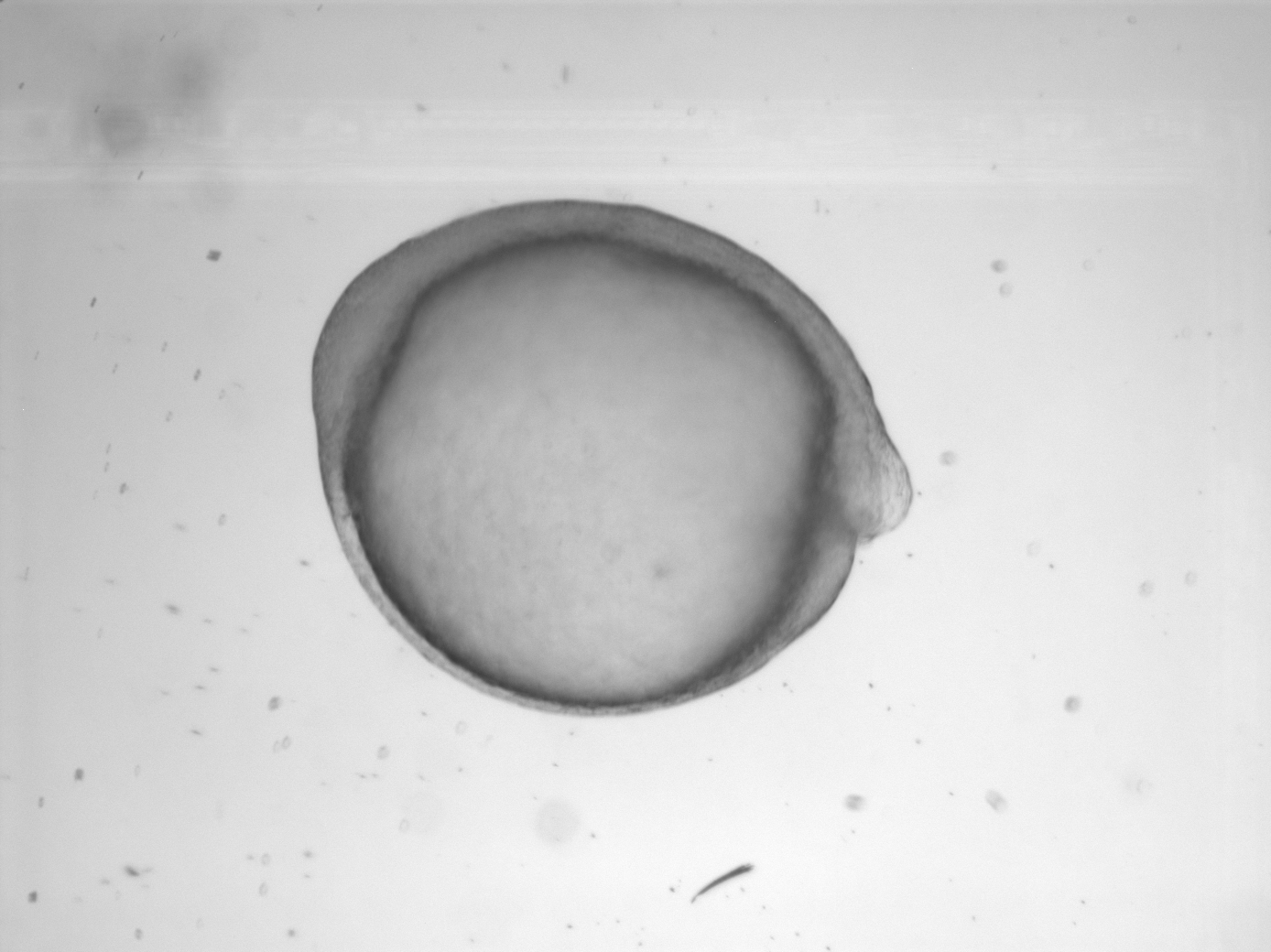

Supplement: Data S1 [file peerj-05-2894-s005.zip › Raw Data/10h-B.jpg]

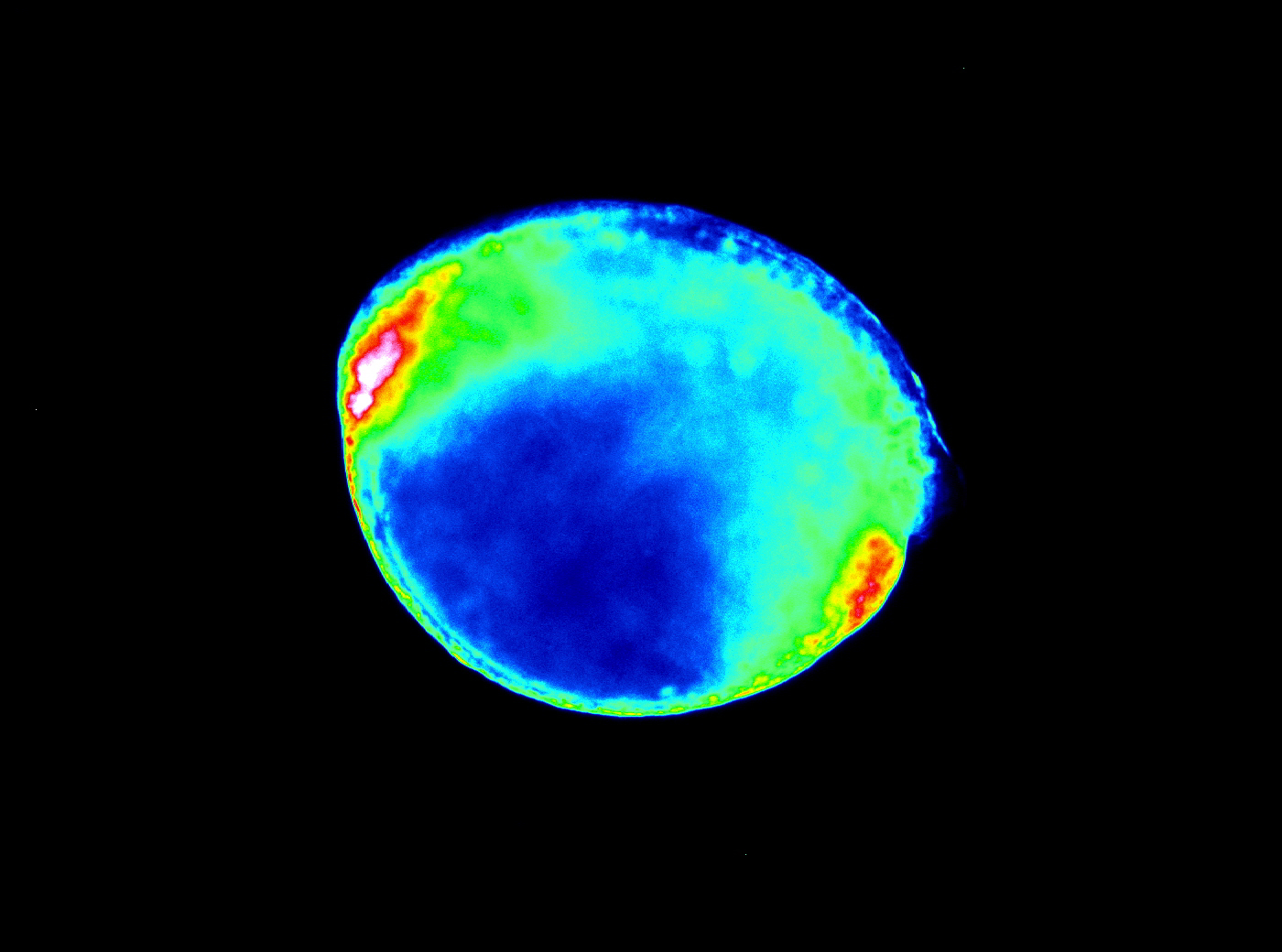

Supplement: Data S1 [file peerj-05-2894-s005.zip › Raw Data/10h-C.jpg]

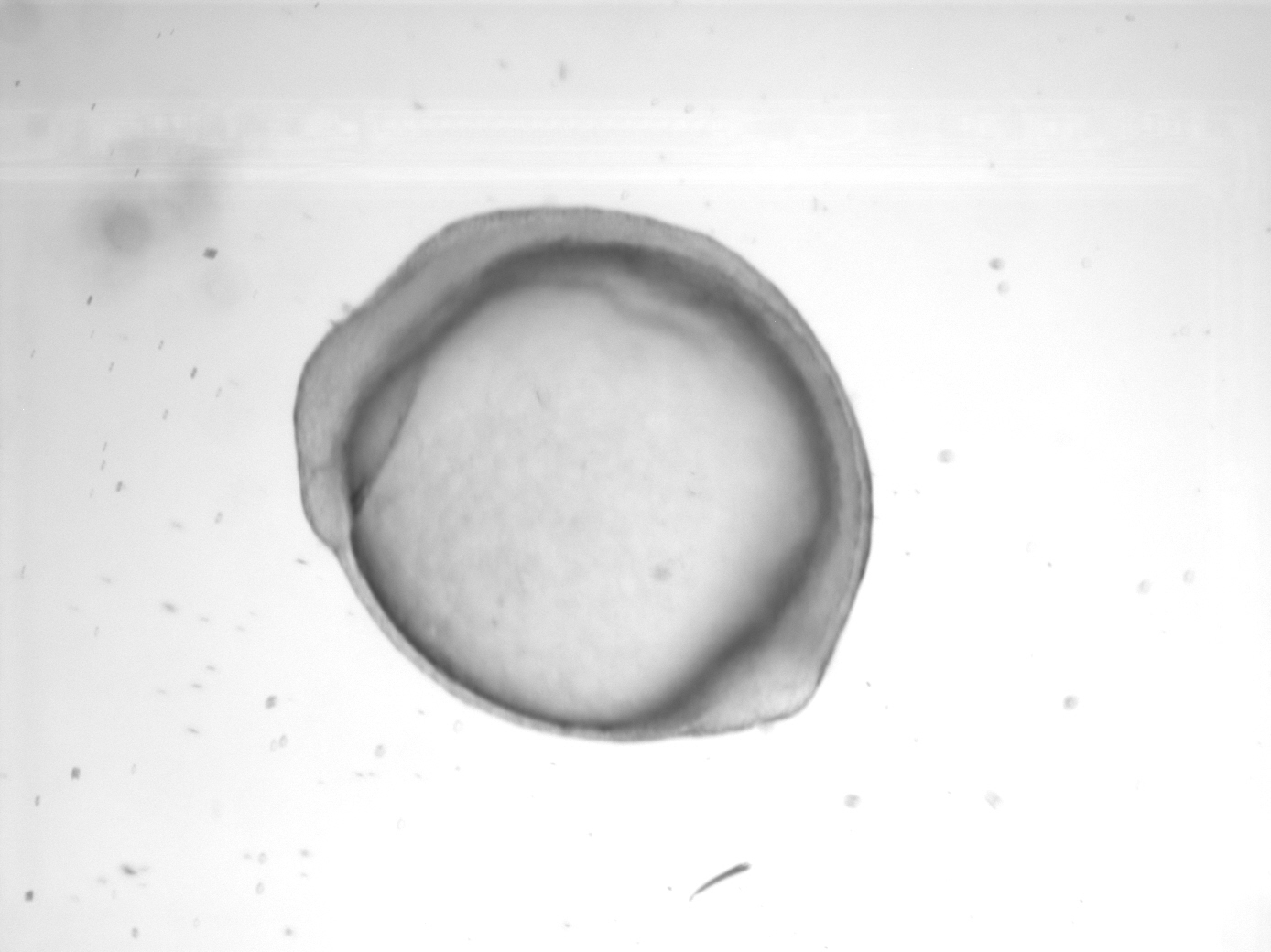

Supplement: Data S1 [file peerj-05-2894-s005.zip › Raw Data/11h-B.jpg]

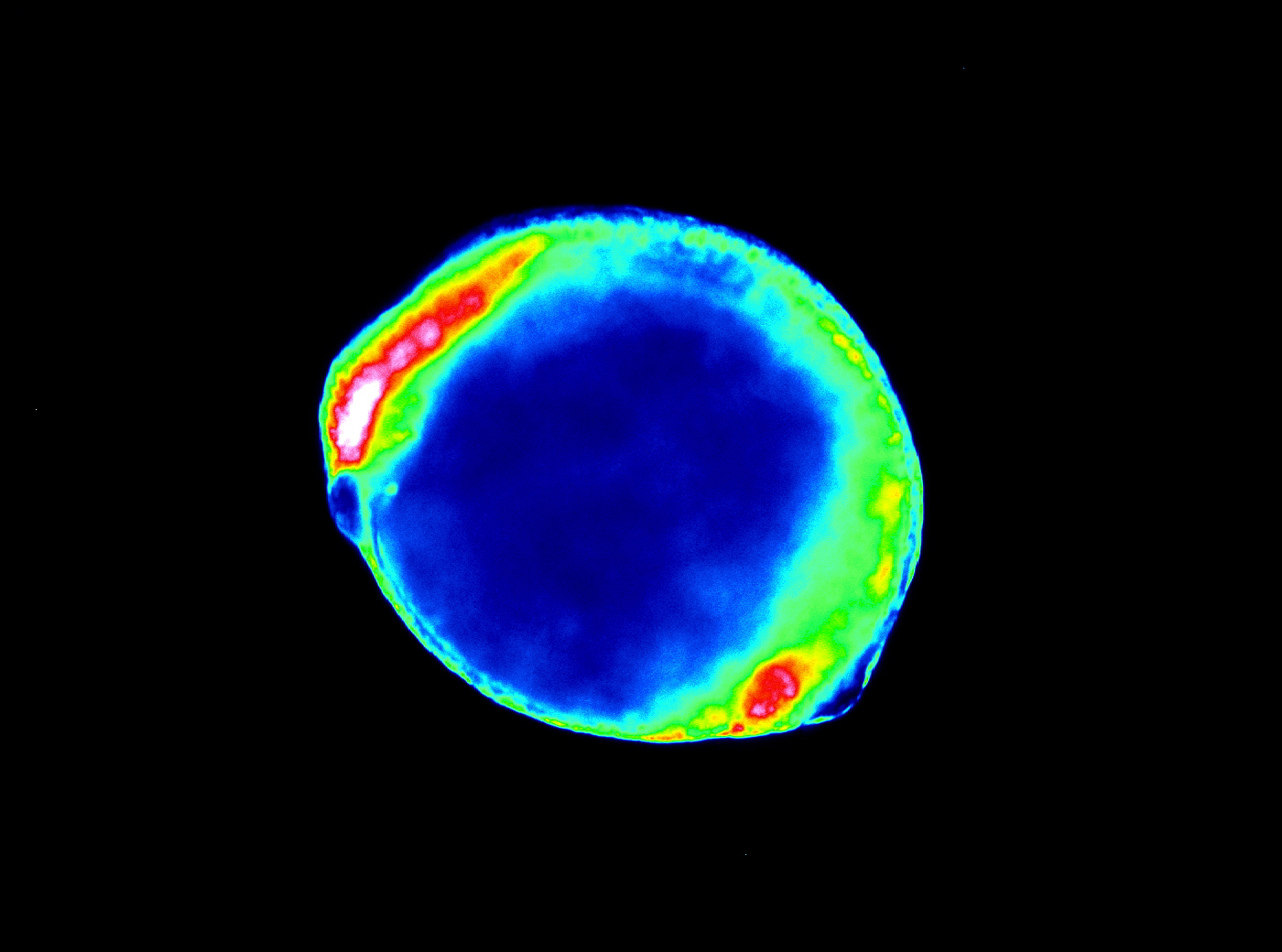

Supplement: Data S1 [file peerj-05-2894-s005.zip › Raw Data/11h-C.jpg]

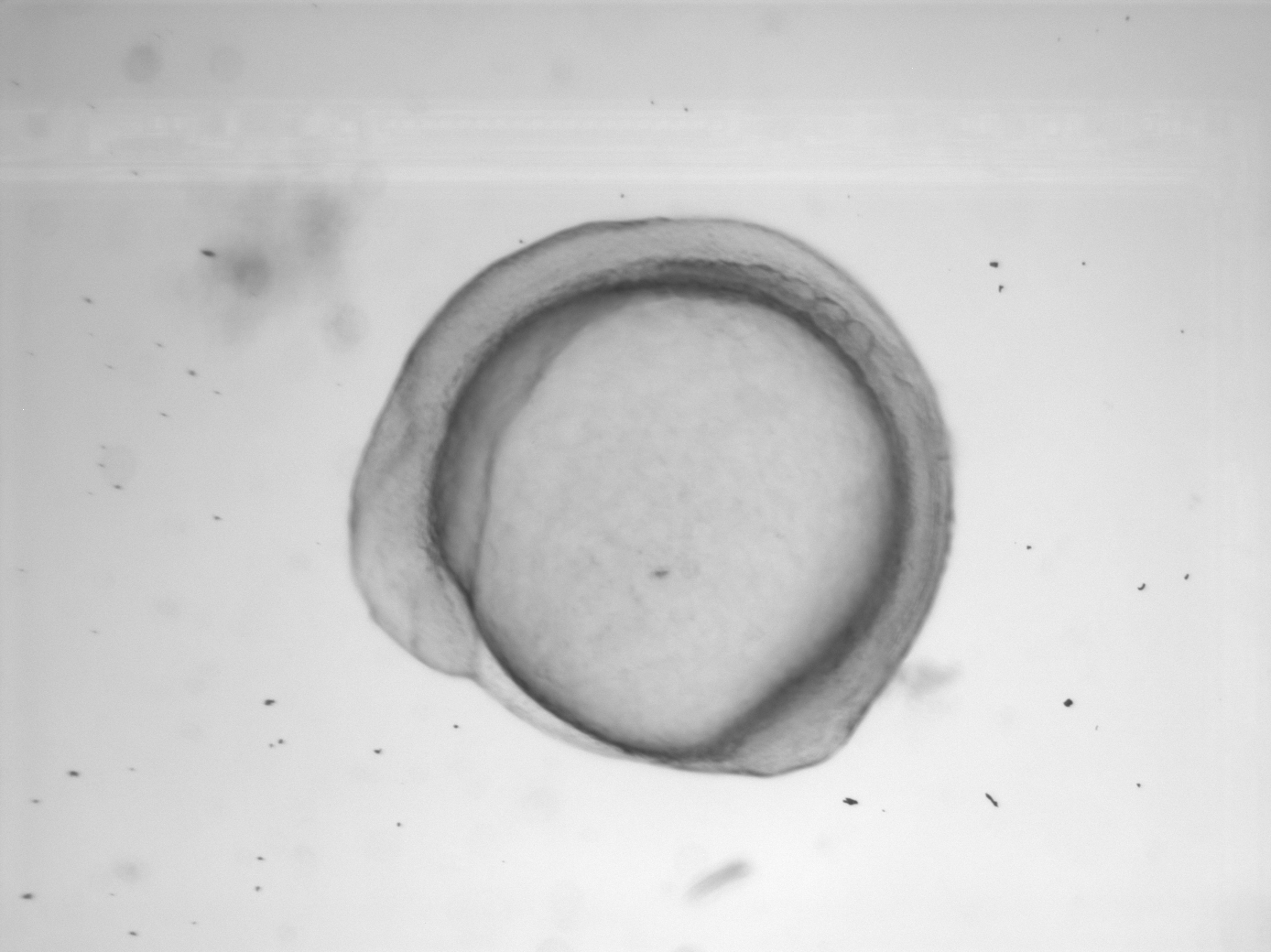

Supplement: Data S1 [file peerj-05-2894-s005.zip › Raw Data/12h-B.jpg]

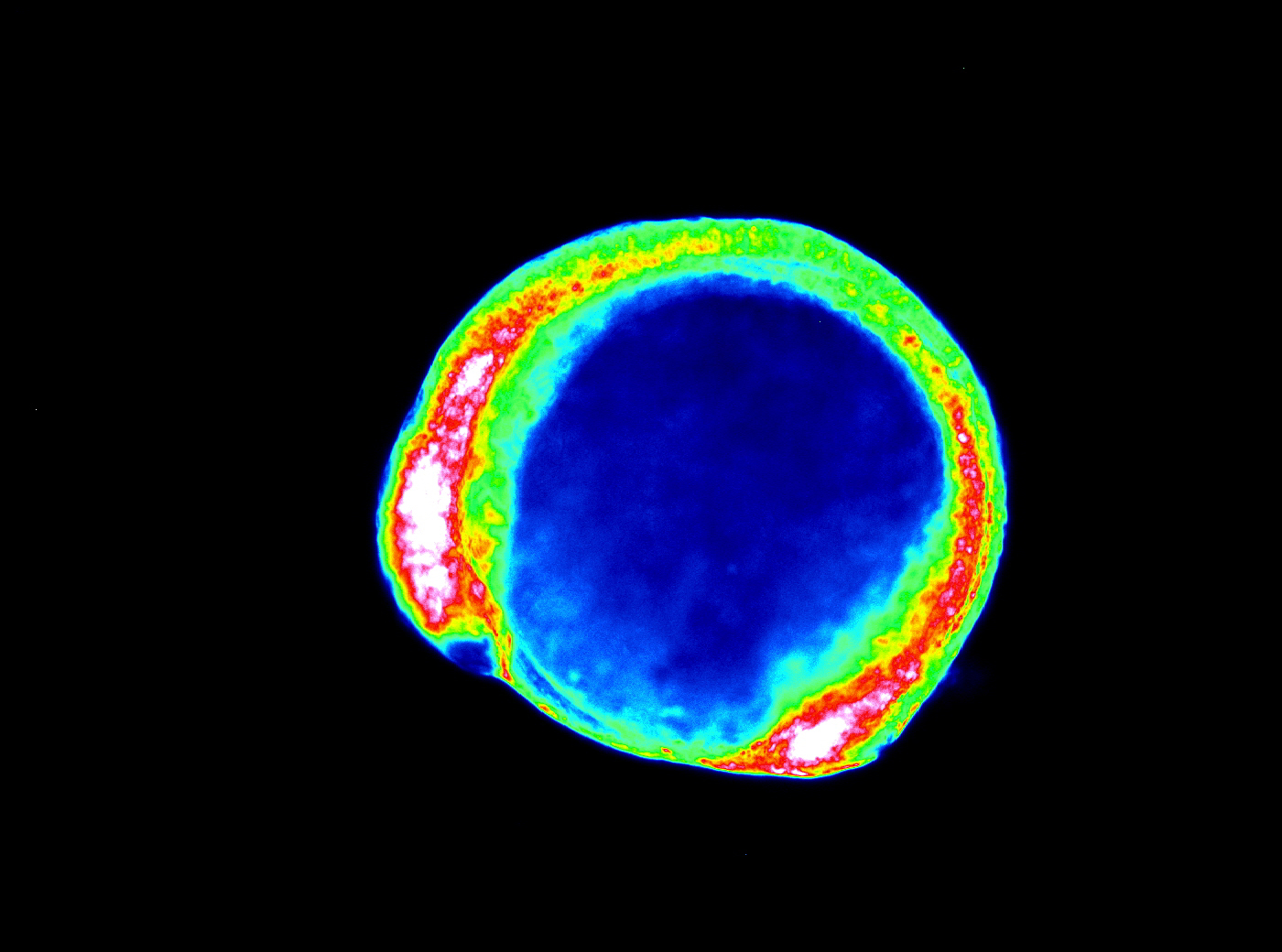

Supplement: Data S1 [file peerj-05-2894-s005.zip › Raw Data/12h-C.jpg]

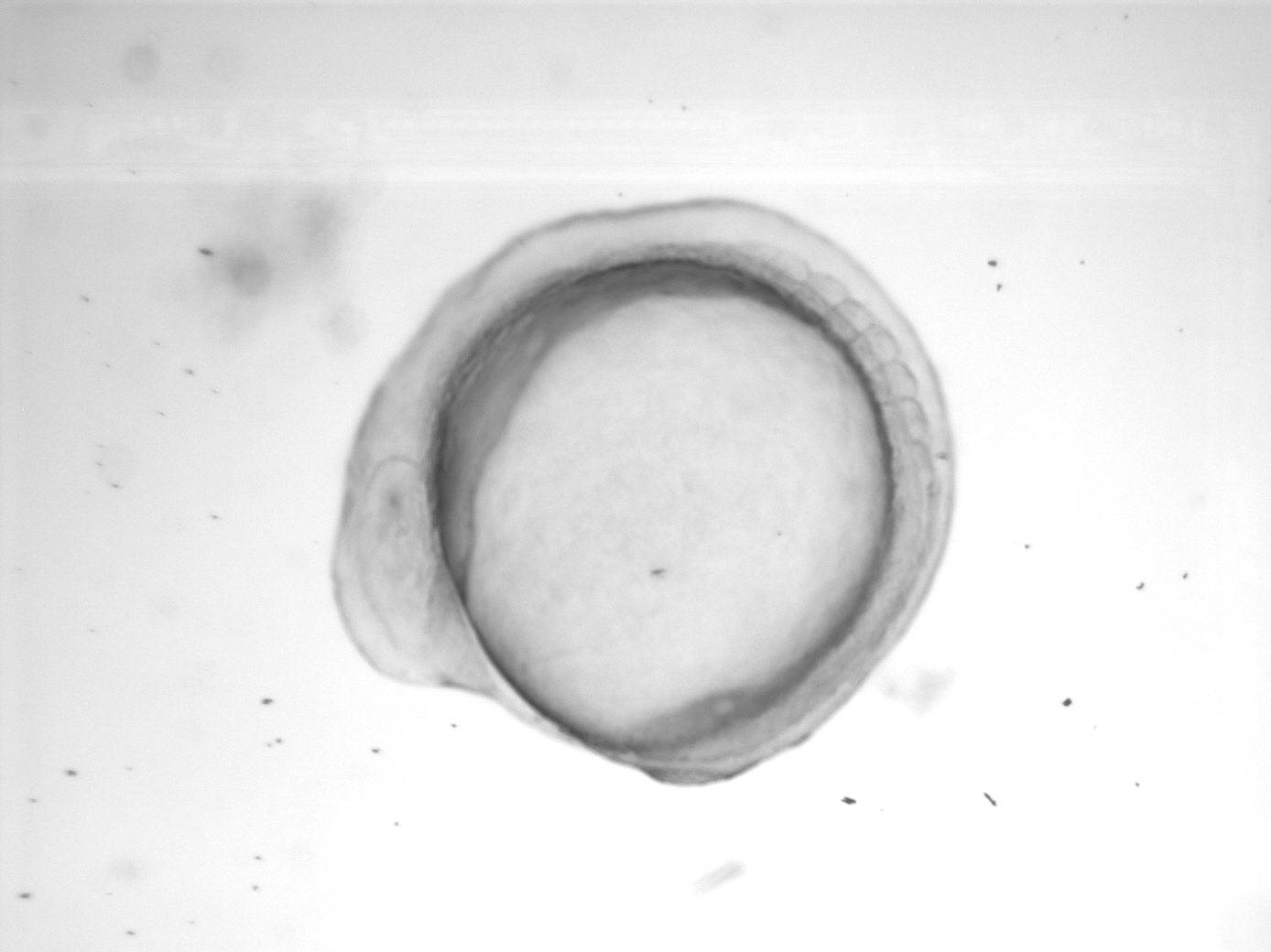

Supplement: Data S1 [file peerj-05-2894-s005.zip › Raw Data/13h-B.jpg]

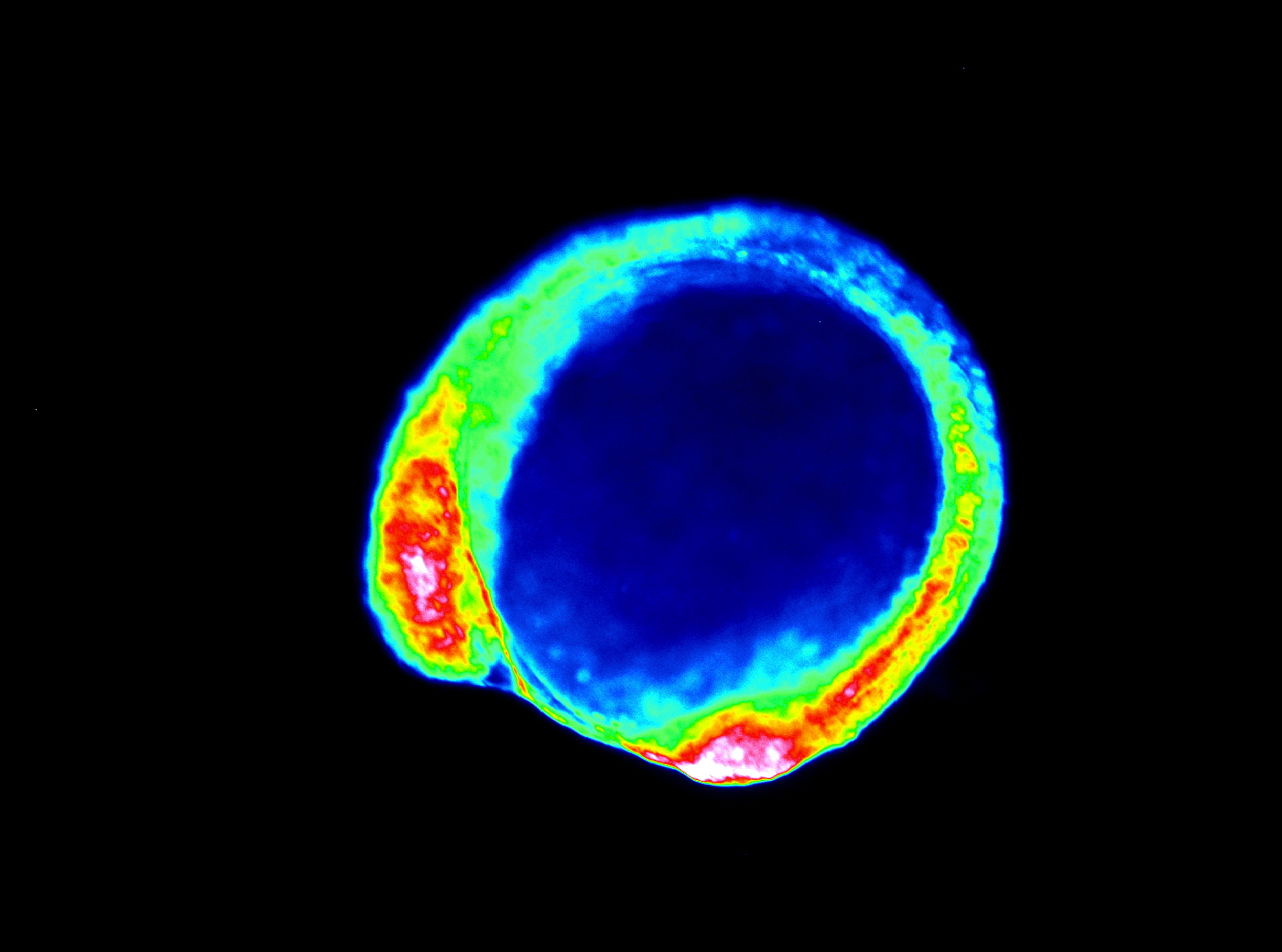

Supplement: Data S1 [file peerj-05-2894-s005.zip › Raw Data/13h-C.jpg]

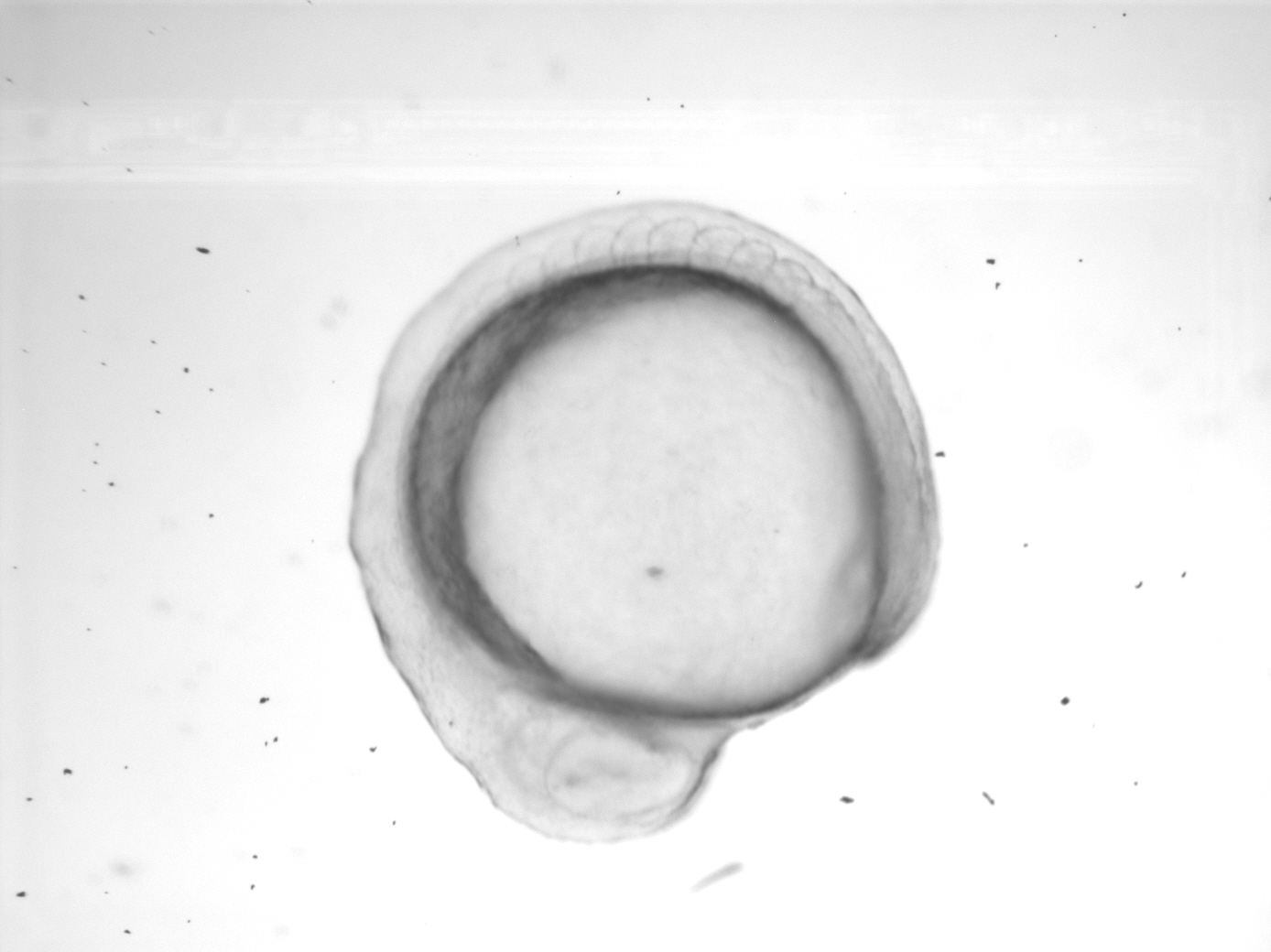

Supplement: Data S1 [file peerj-05-2894-s005.zip › Raw Data/14h-B.jpg]

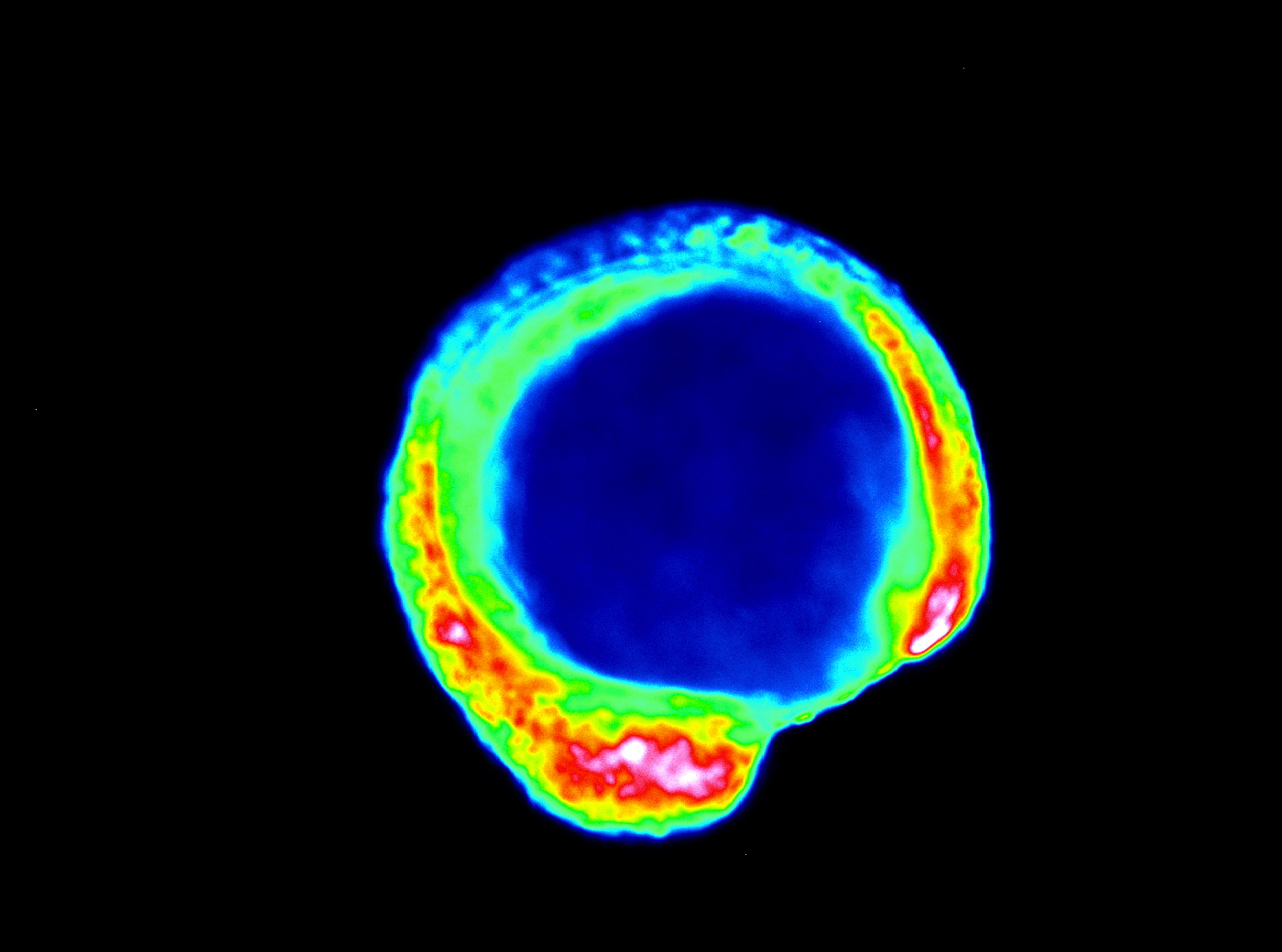

Supplement: Data S1 [file peerj-05-2894-s005.zip › Raw Data/14h-C.jpg]

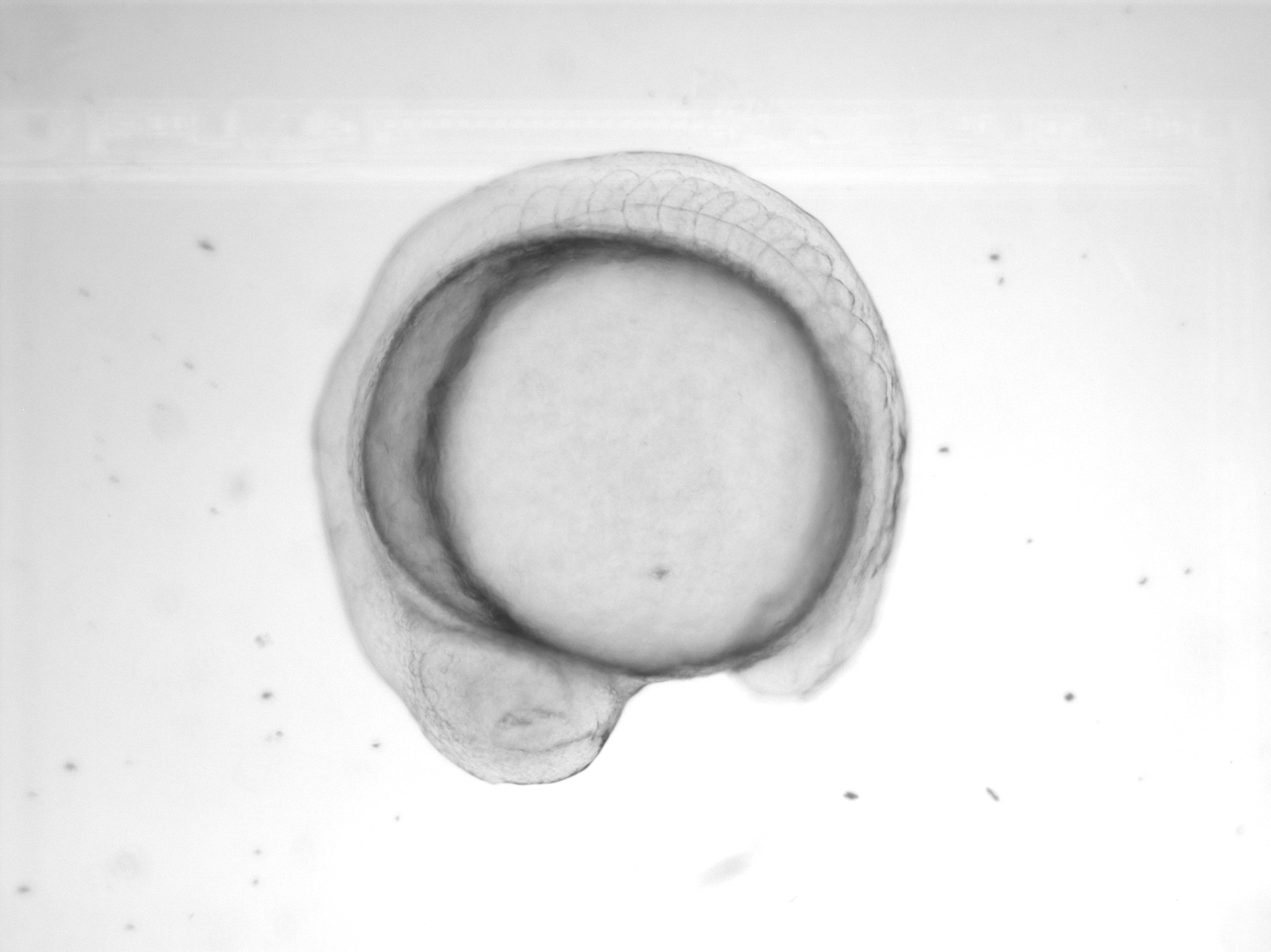

Supplement: Data S1 [file peerj-05-2894-s005.zip › Raw Data/15h-B.jpg]

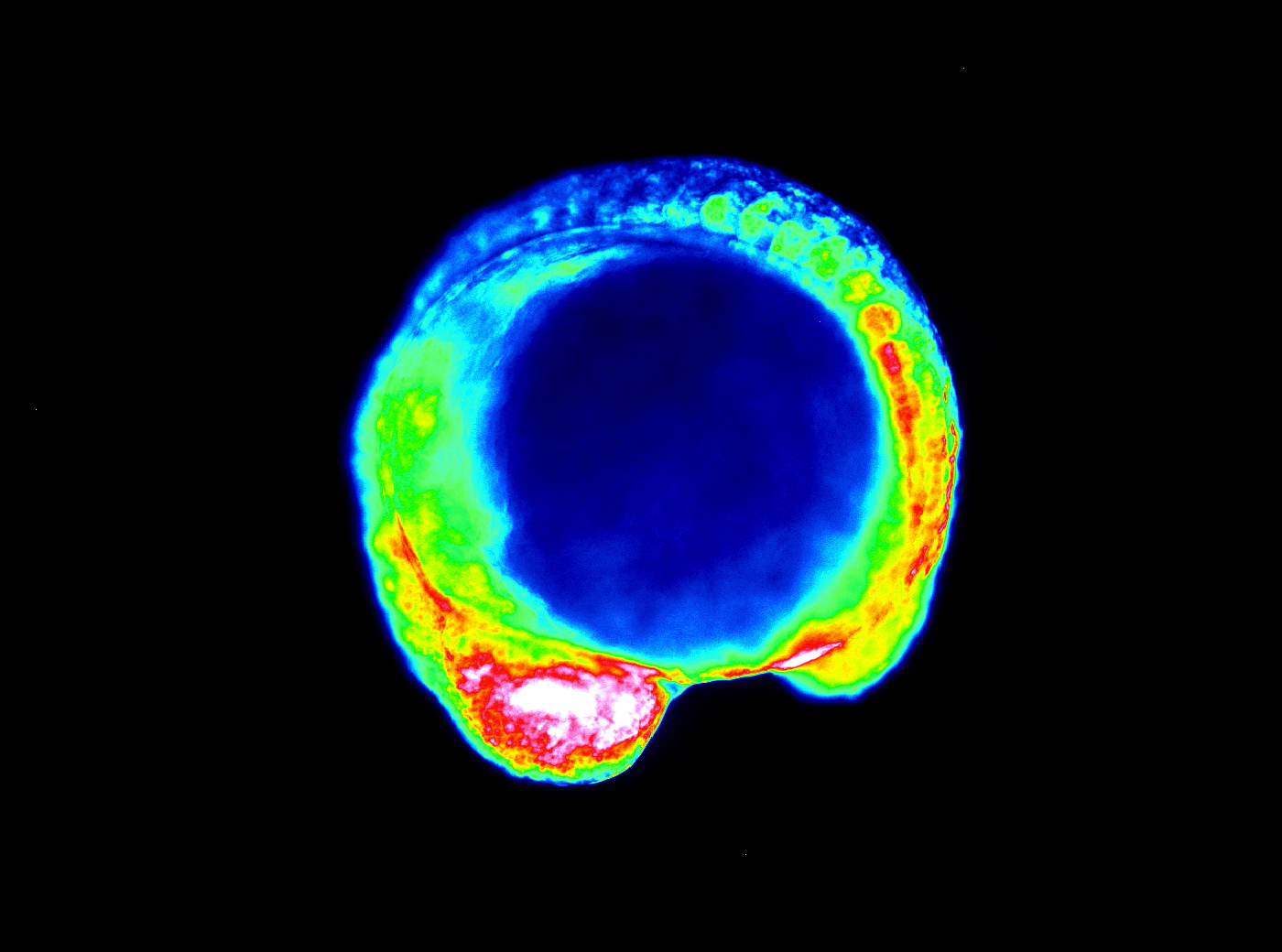

Supplement: Data S1 [file peerj-05-2894-s005.zip › Raw Data/15h-C.jpg]

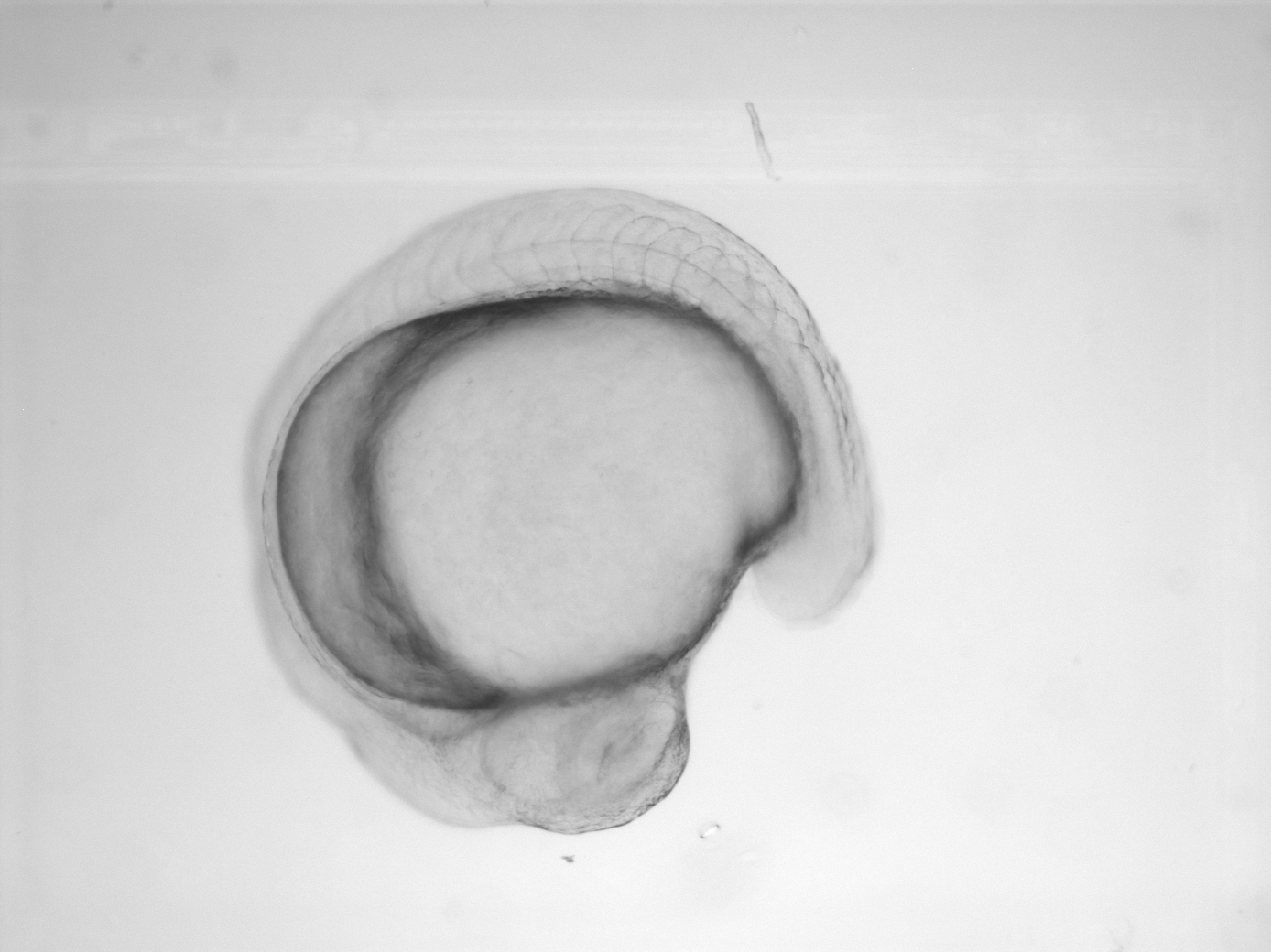

Supplement: Data S1 [file peerj-05-2894-s005.zip › Raw Data/16h-B.jpg]

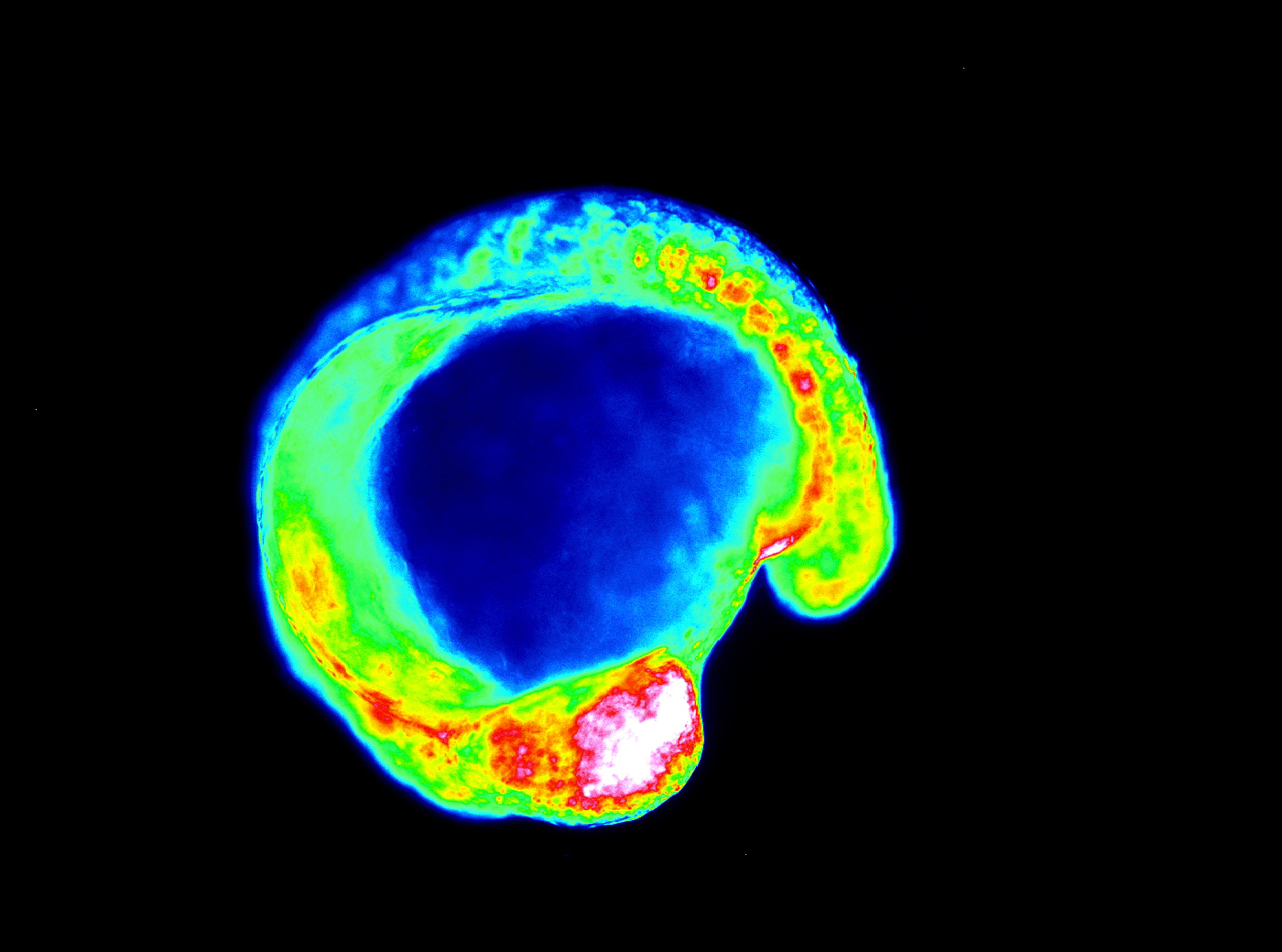

Supplement: Data S1 [file peerj-05-2894-s005.zip › Raw Data/16h-C.jpg]

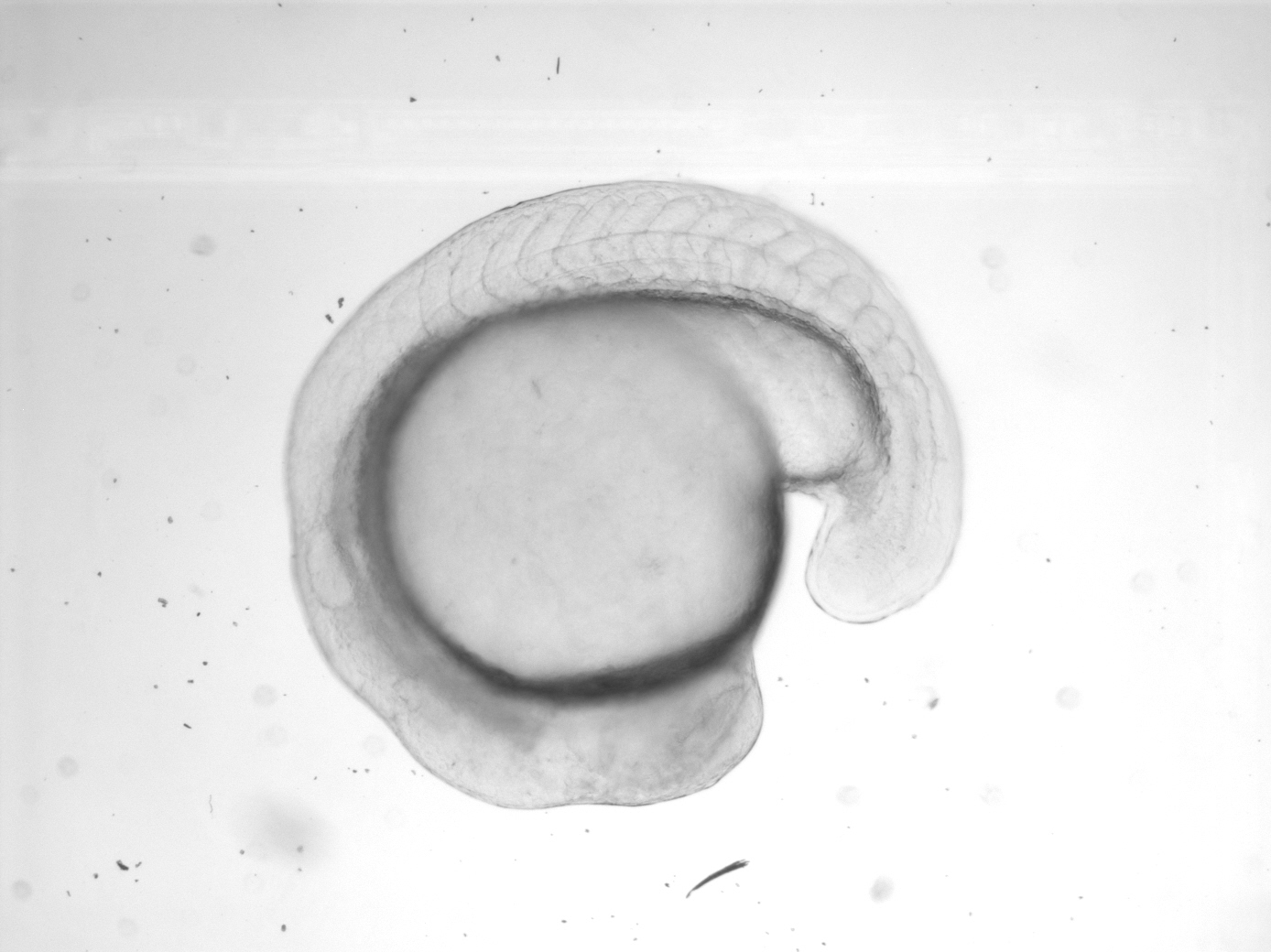

Supplement: Data S1 [file peerj-05-2894-s005.zip › Raw Data/17h-B.jpg]

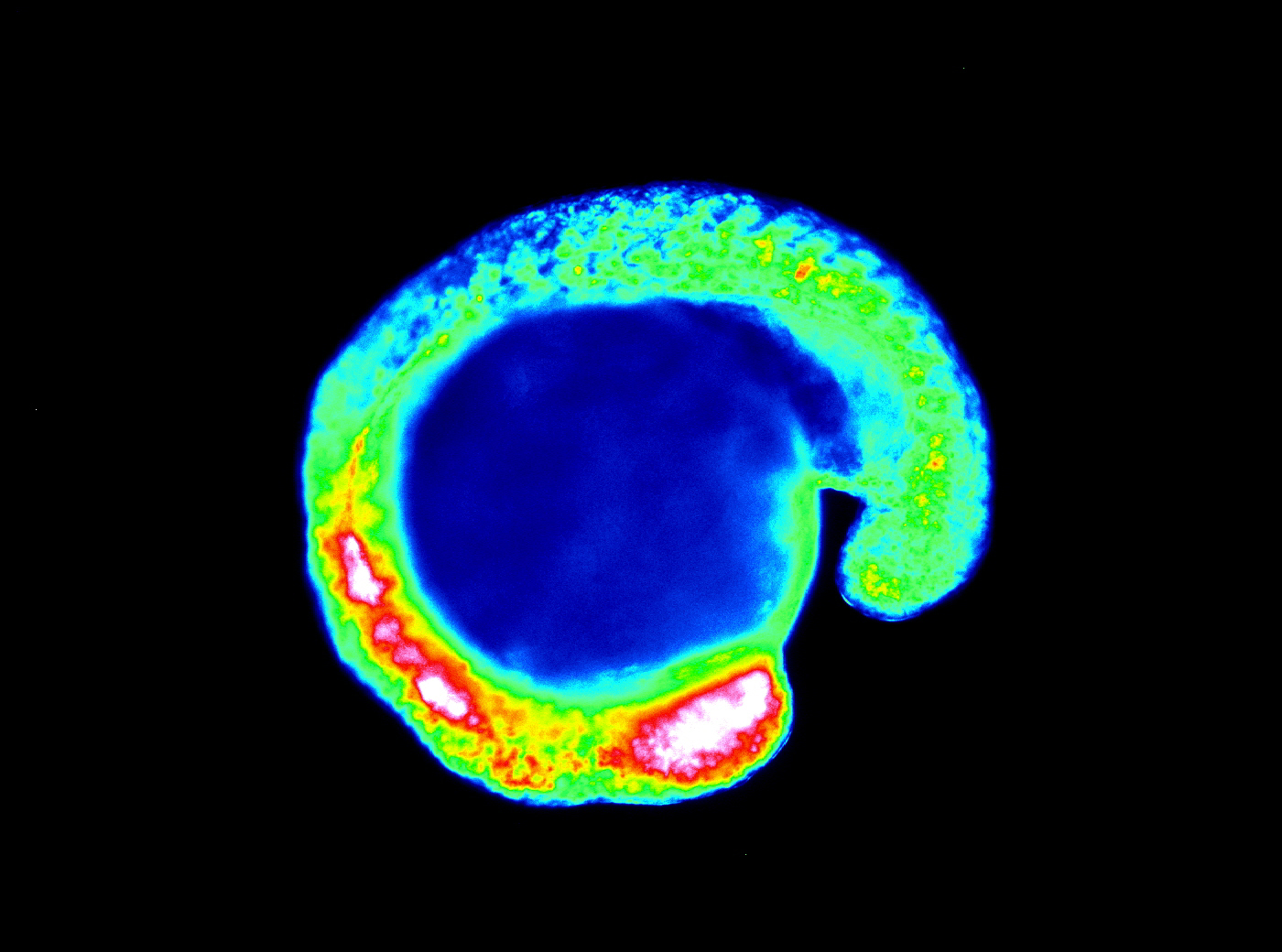

Supplement: Data S1 [file peerj-05-2894-s005.zip › Raw Data/17h-C.jpg]

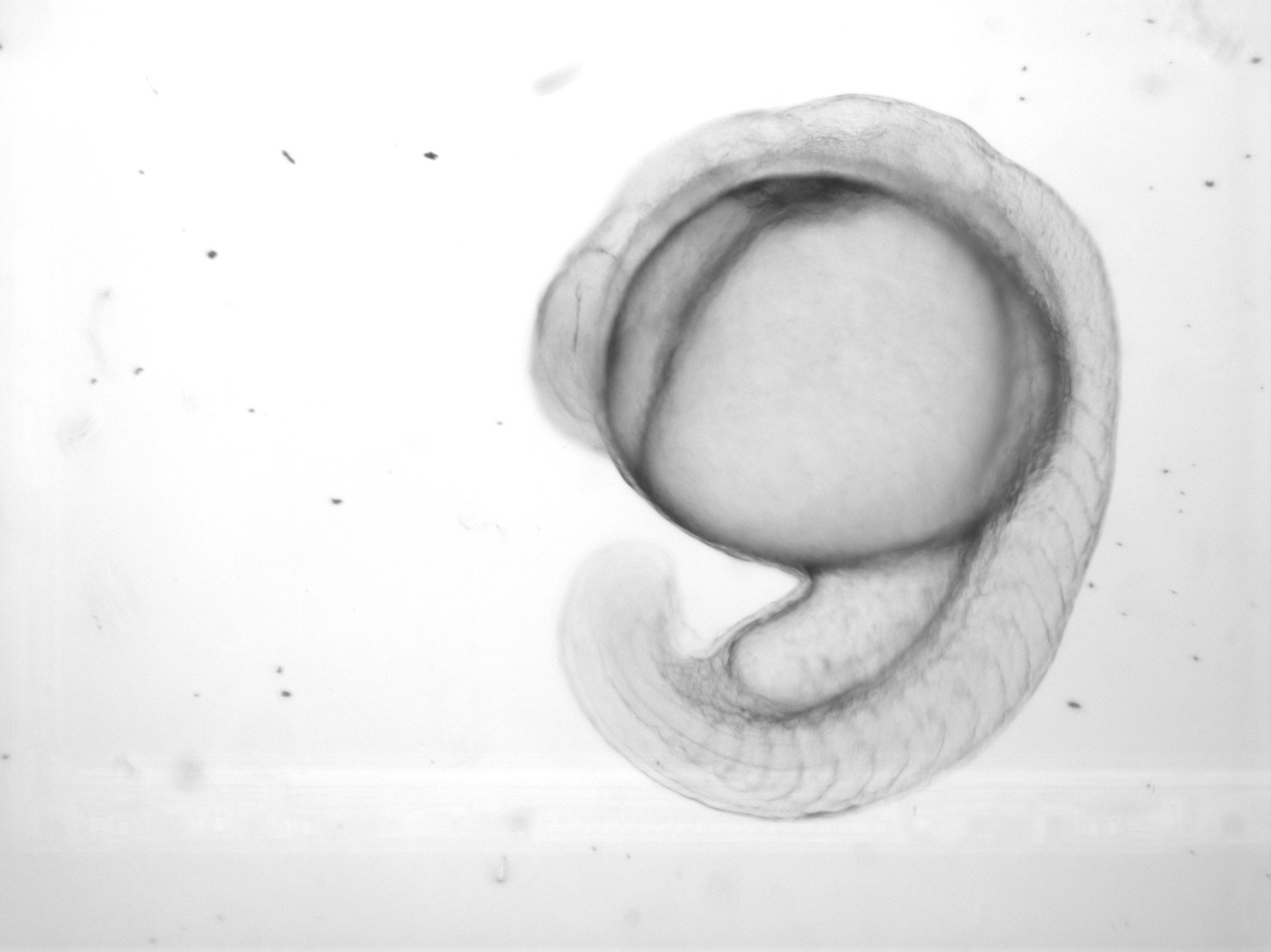

Supplement: Data S1 [file peerj-05-2894-s005.zip › Raw Data/18h-B.jpg]

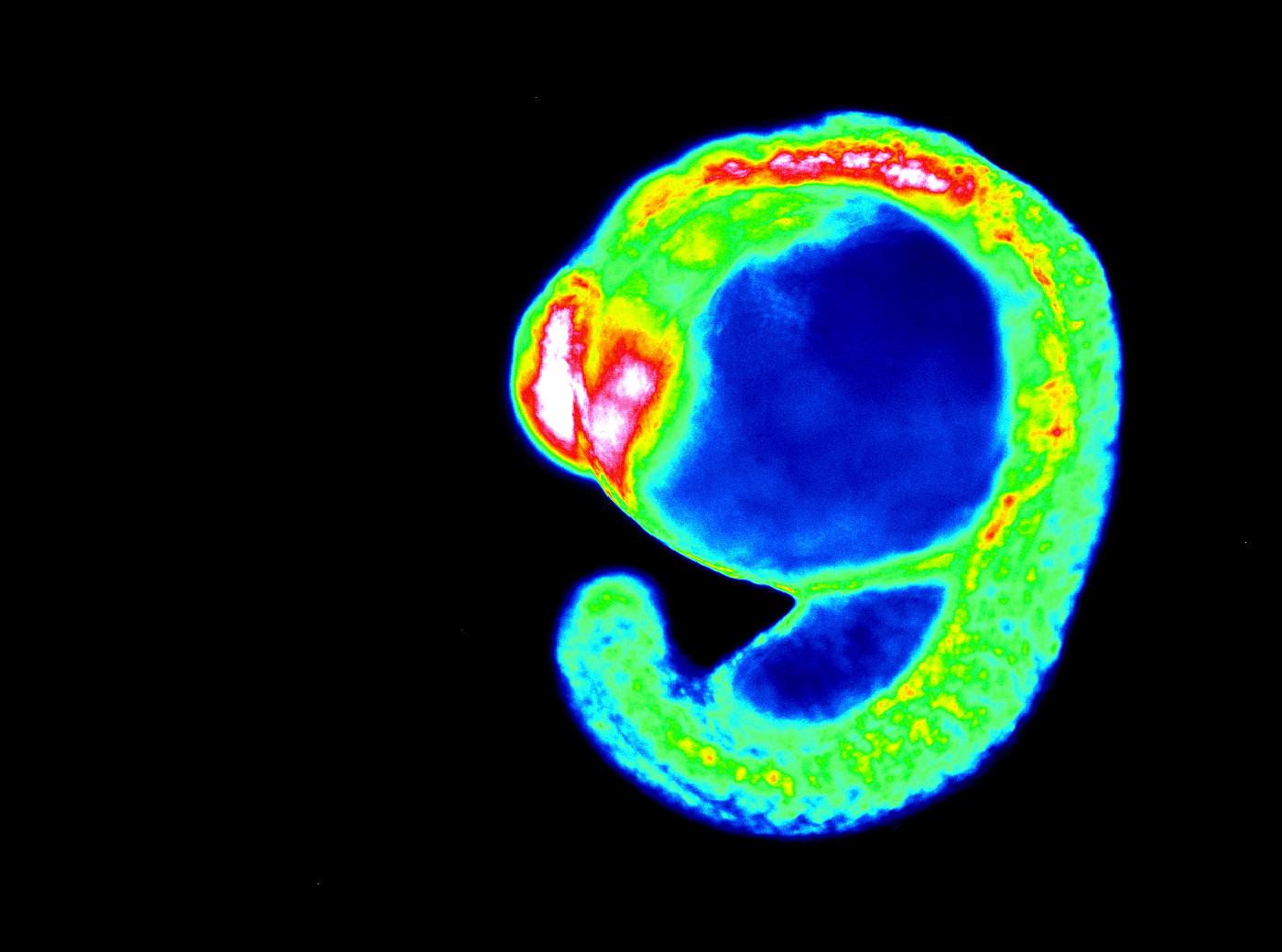

Supplement: Data S1 [file peerj-05-2894-s005.zip › Raw Data/18h-C.jpg]

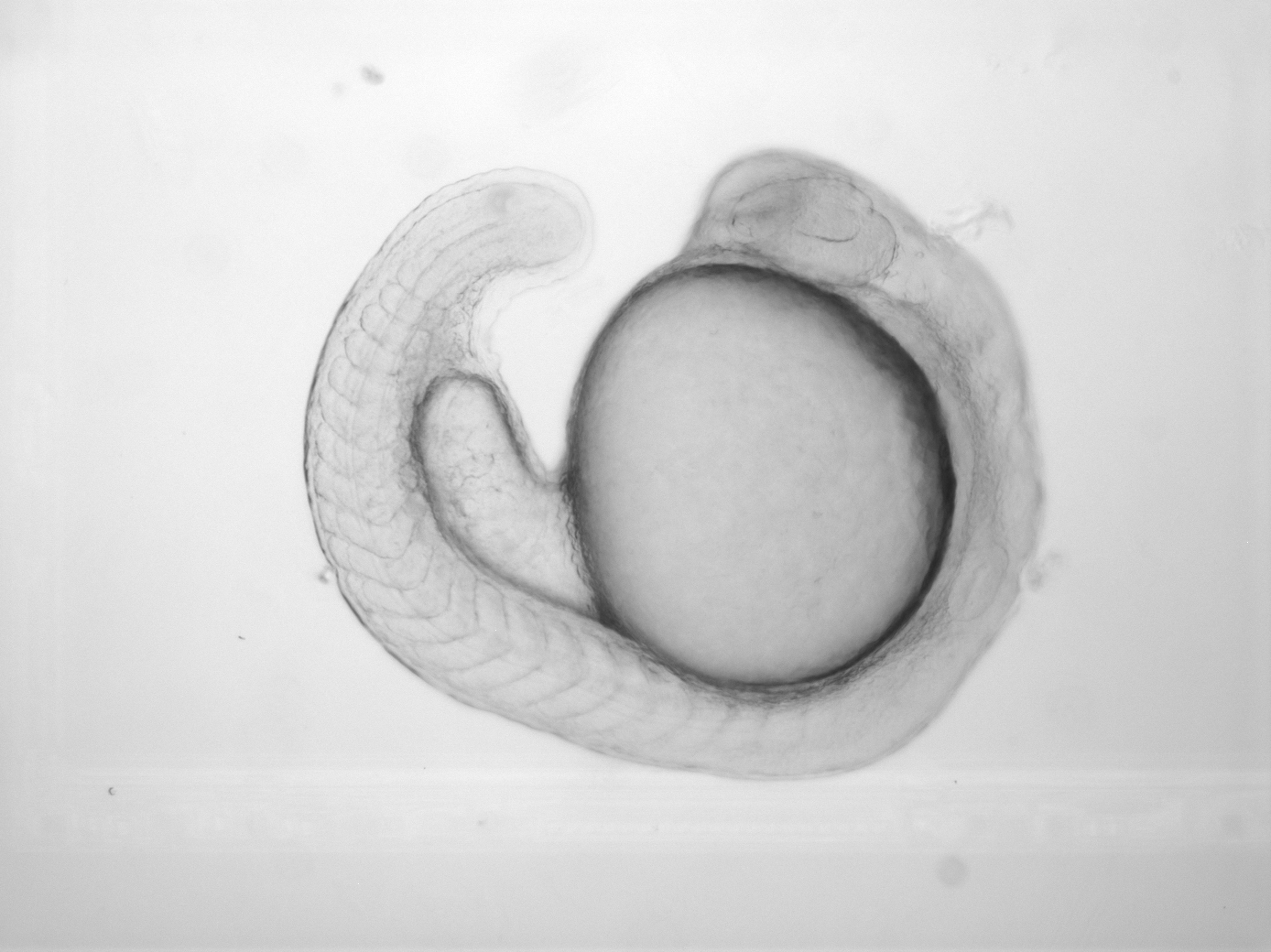

Supplement: Data S1 [file peerj-05-2894-s005.zip › Raw Data/19h-B.jpg]

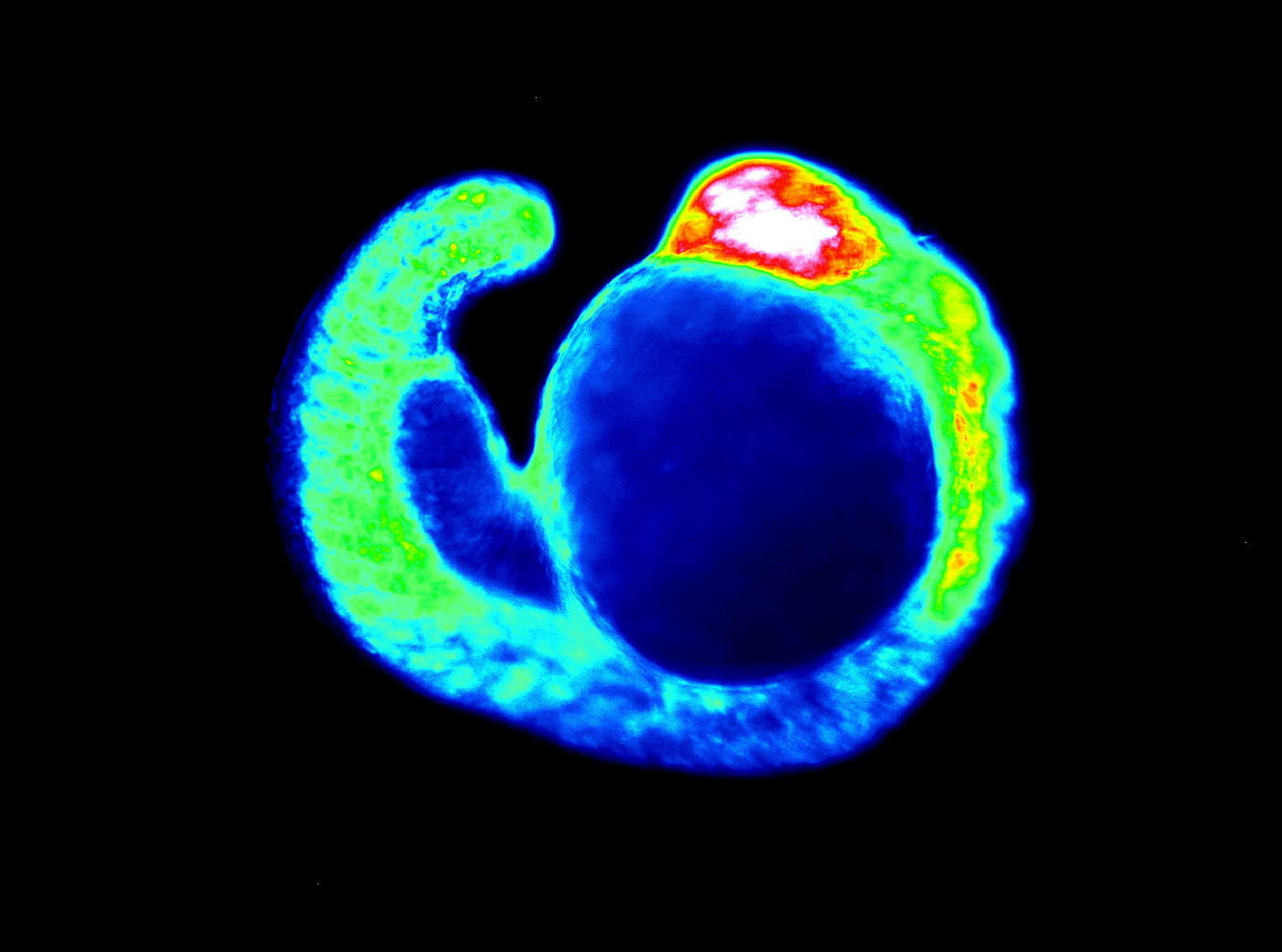

Supplement: Data S1 [file peerj-05-2894-s005.zip › Raw Data/19h-C.jpg]

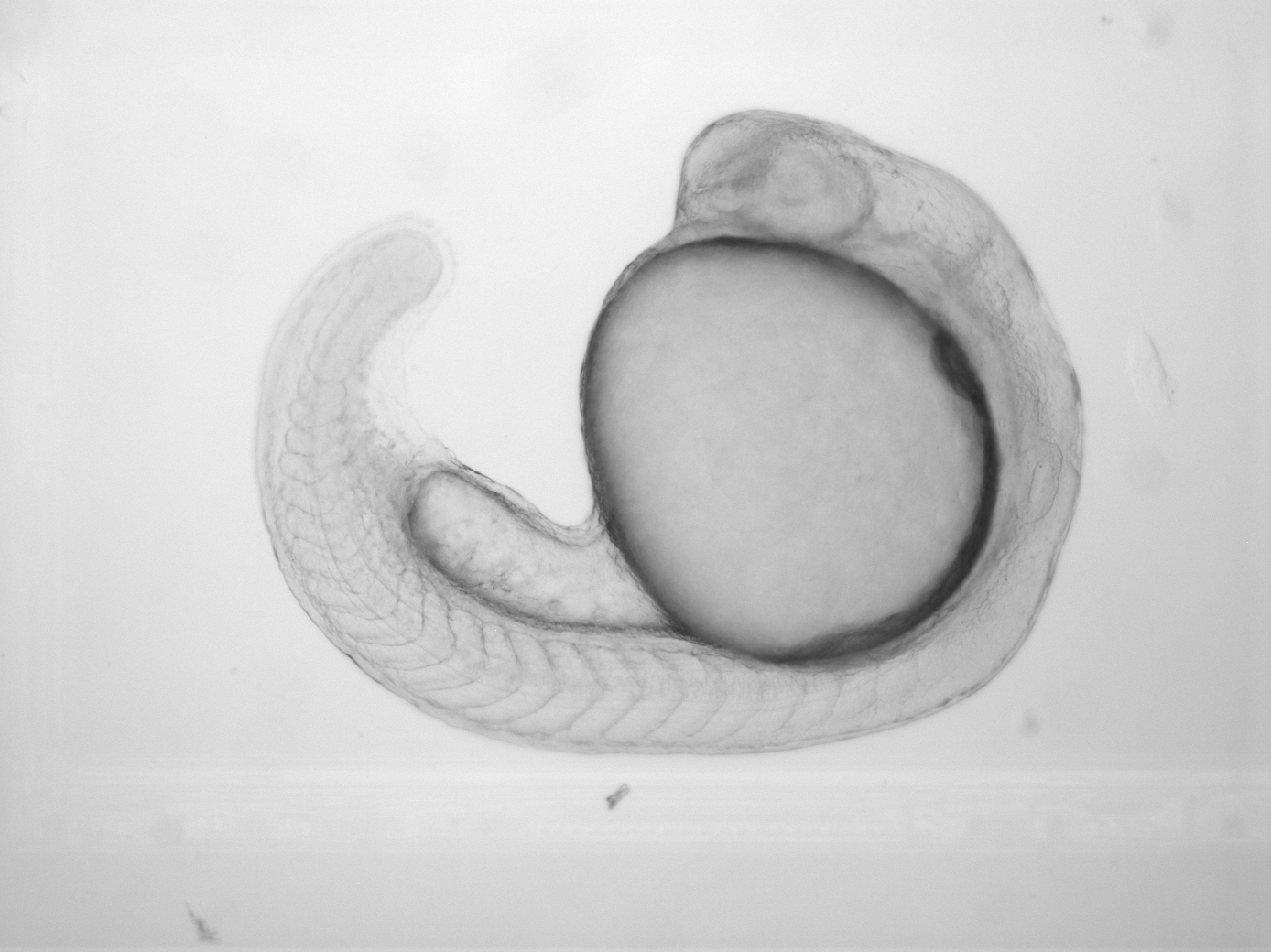

Supplement: Data S1 [file peerj-05-2894-s005.zip › Raw Data/20h-B.jpg]

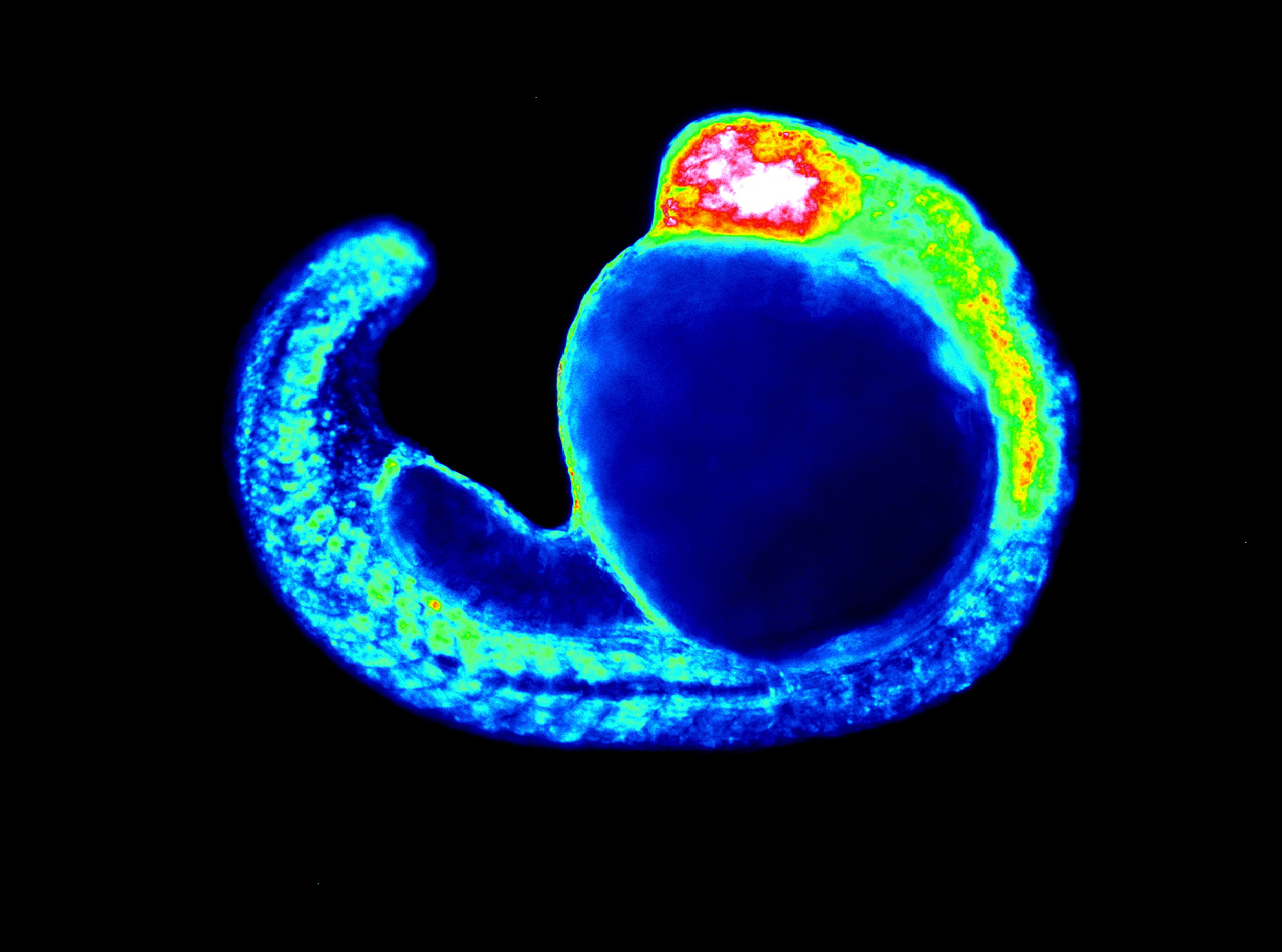

Supplement: Data S1 [file peerj-05-2894-s005.zip › Raw Data/20h-C.jpg]

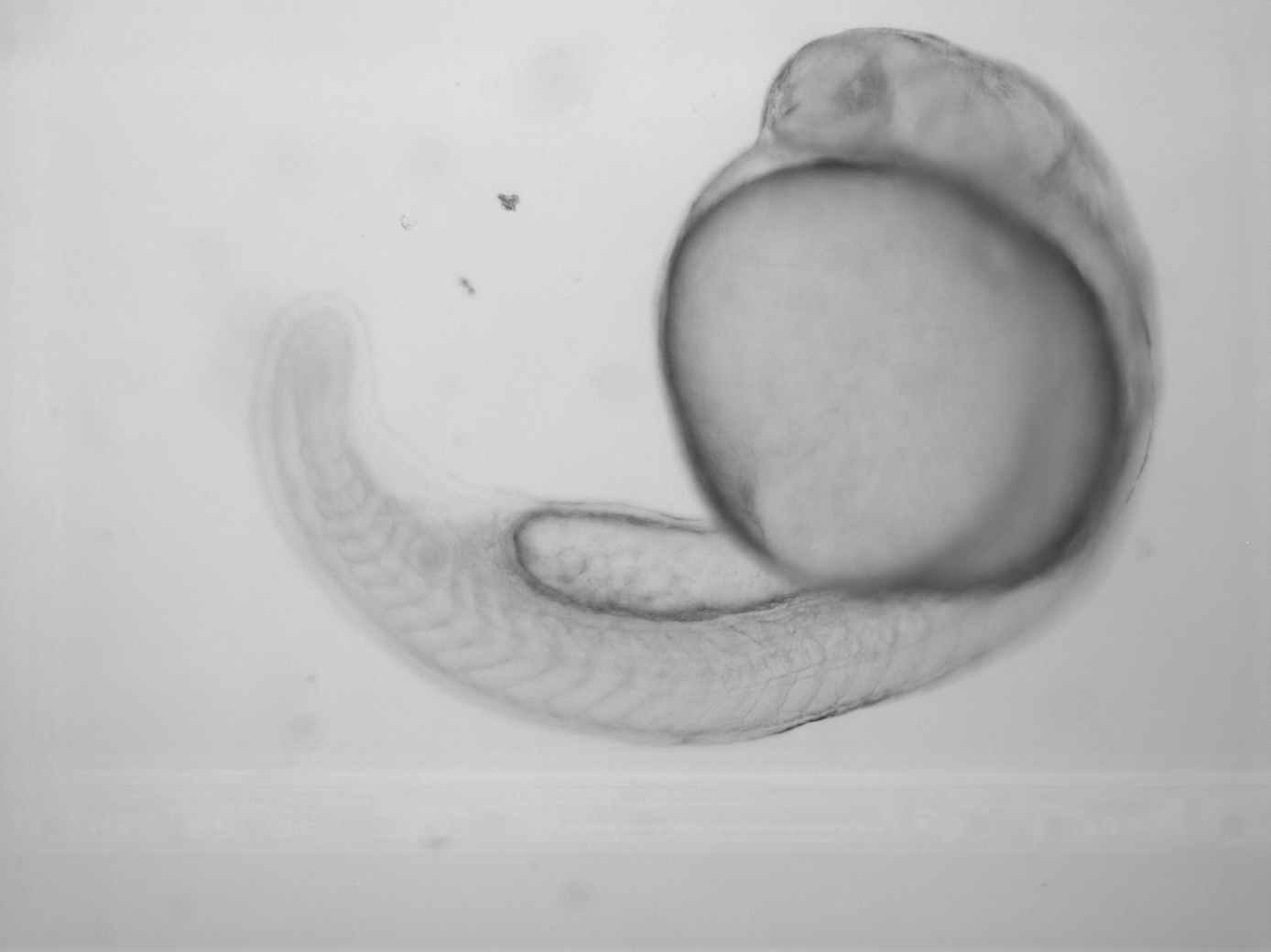

Supplement: Data S1 [file peerj-05-2894-s005.zip › Raw Data/21h-B.jpg]

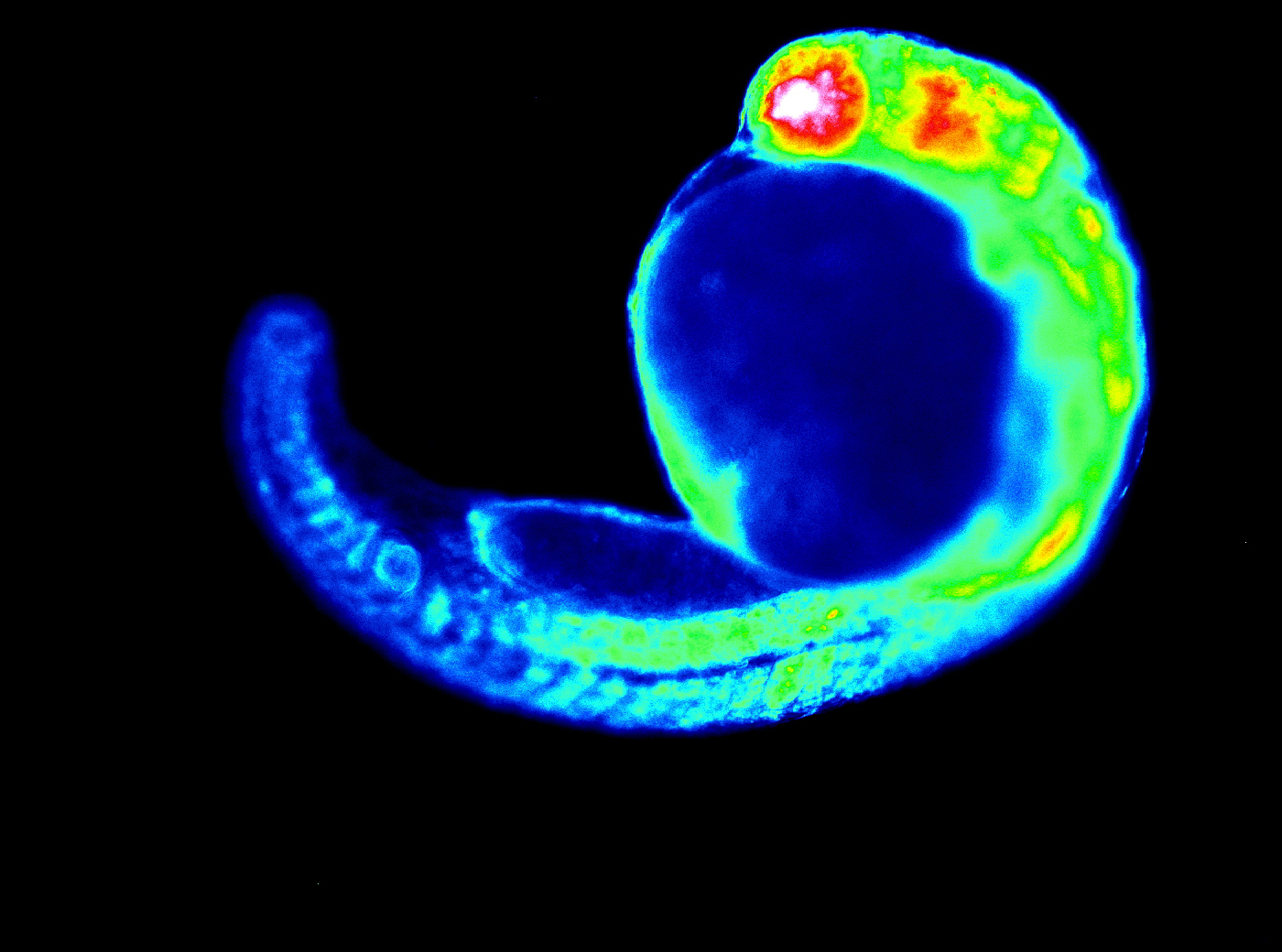

Supplement: Data S1 [file peerj-05-2894-s005.zip › Raw Data/21h-C.jpg]

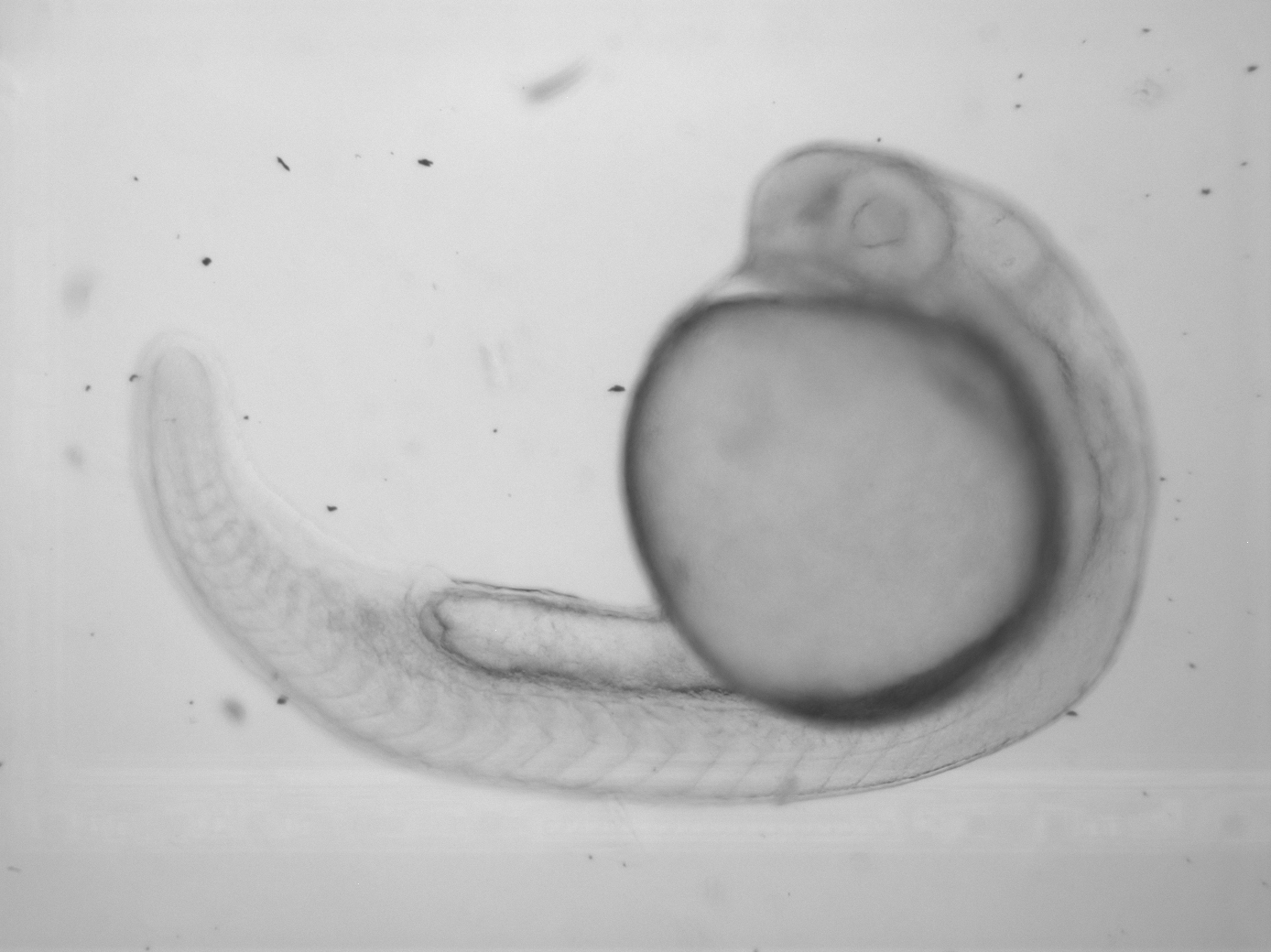

Supplement: Data S1 [file peerj-05-2894-s005.zip › Raw Data/22h-B.jpg]

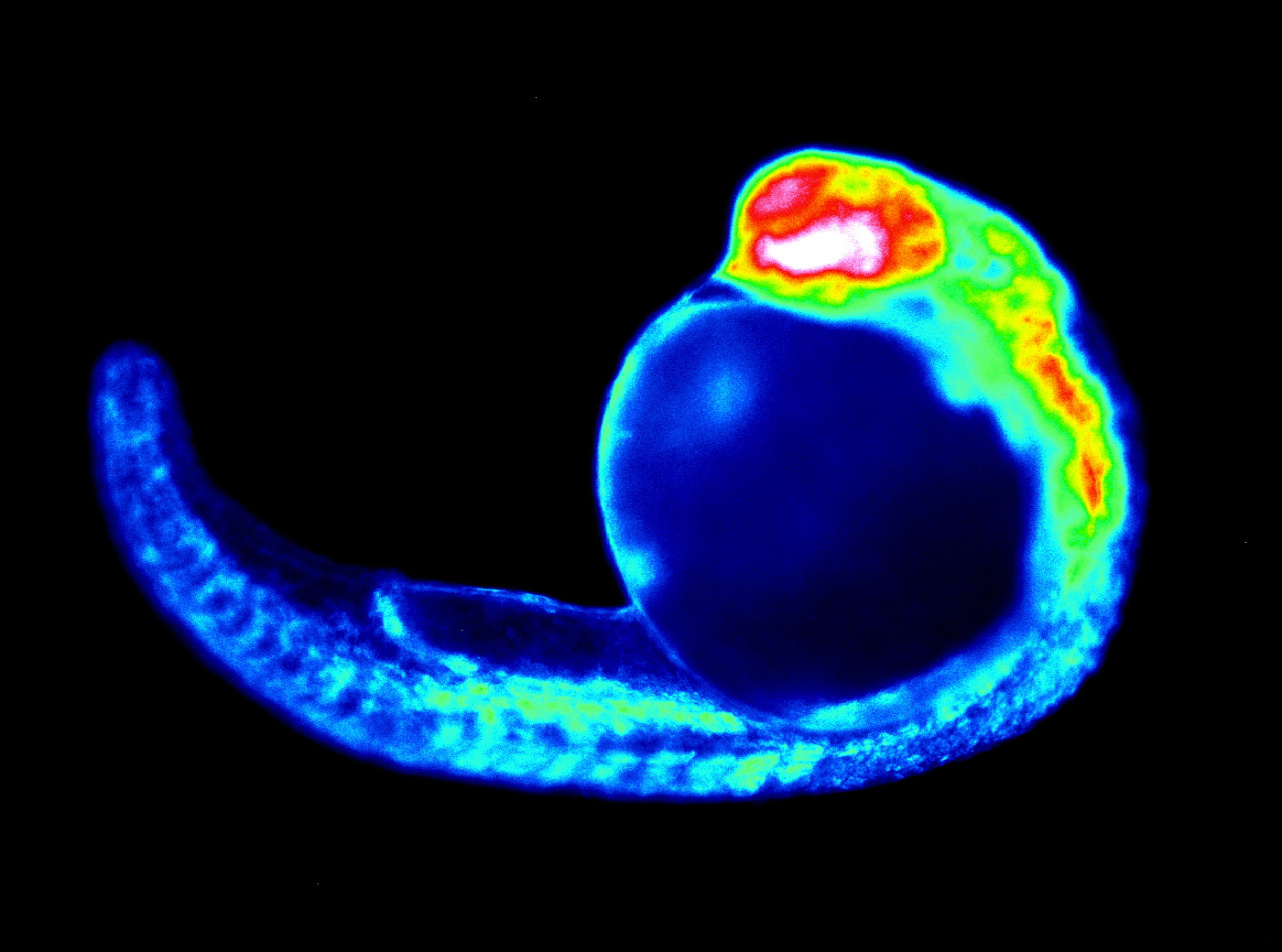

Supplement: Data S1 [file peerj-05-2894-s005.zip › Raw Data/22h-C.jpg]

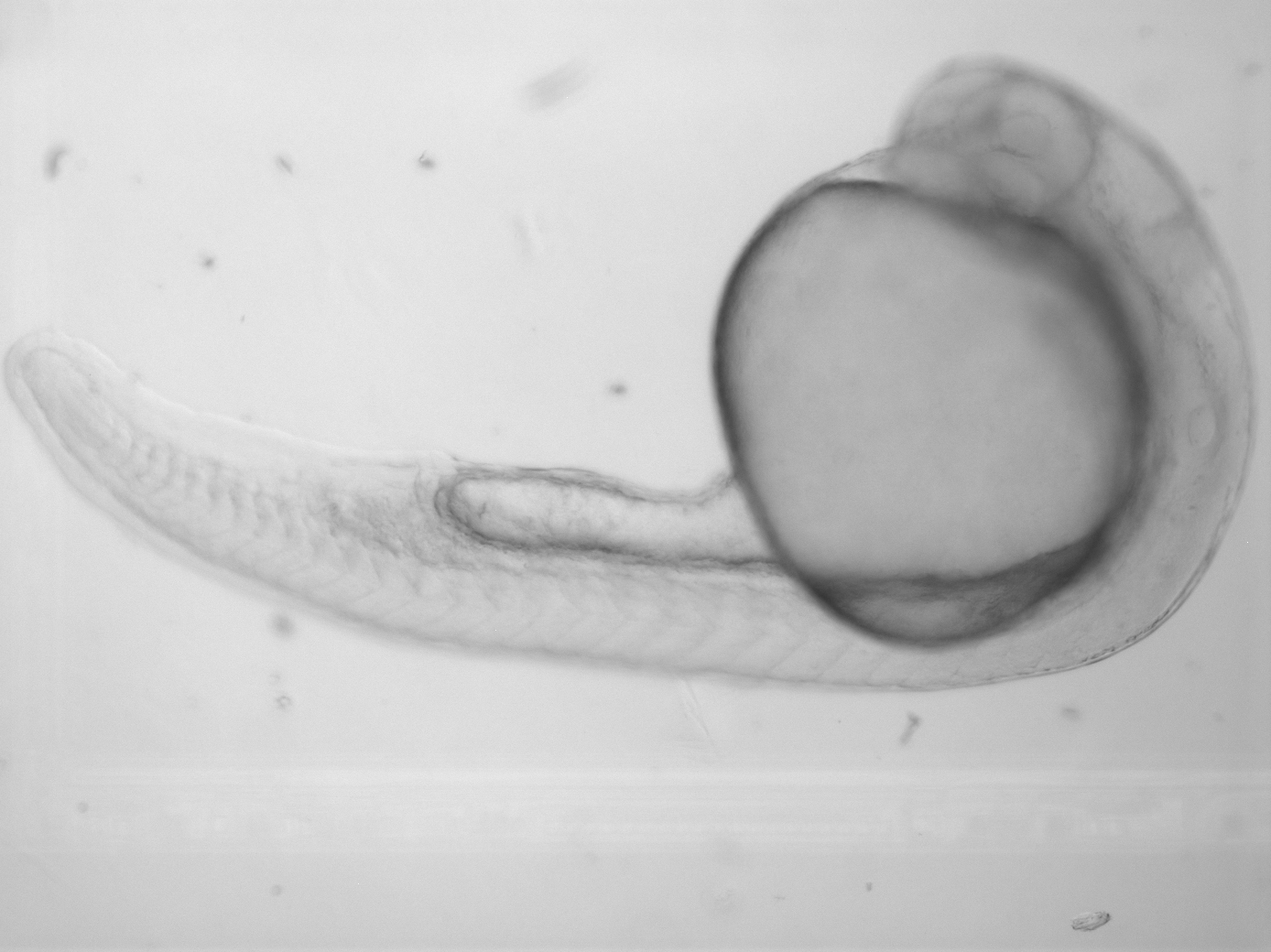

Supplement: Data S1 [file peerj-05-2894-s005.zip › Raw Data/23h-B.jpg]

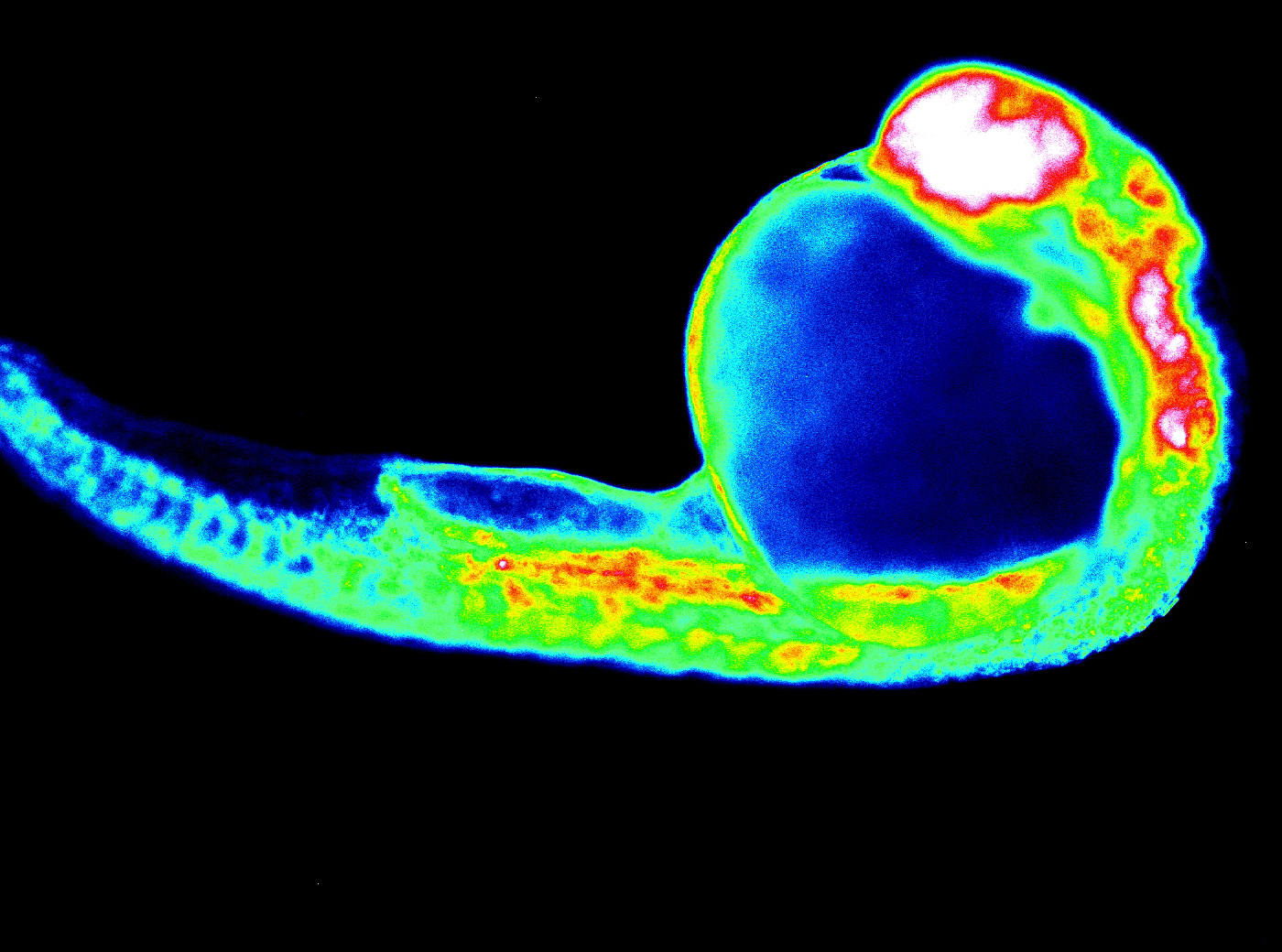

Supplement: Data S1 [file peerj-05-2894-s005.zip › Raw Data/23h-C.jpg]

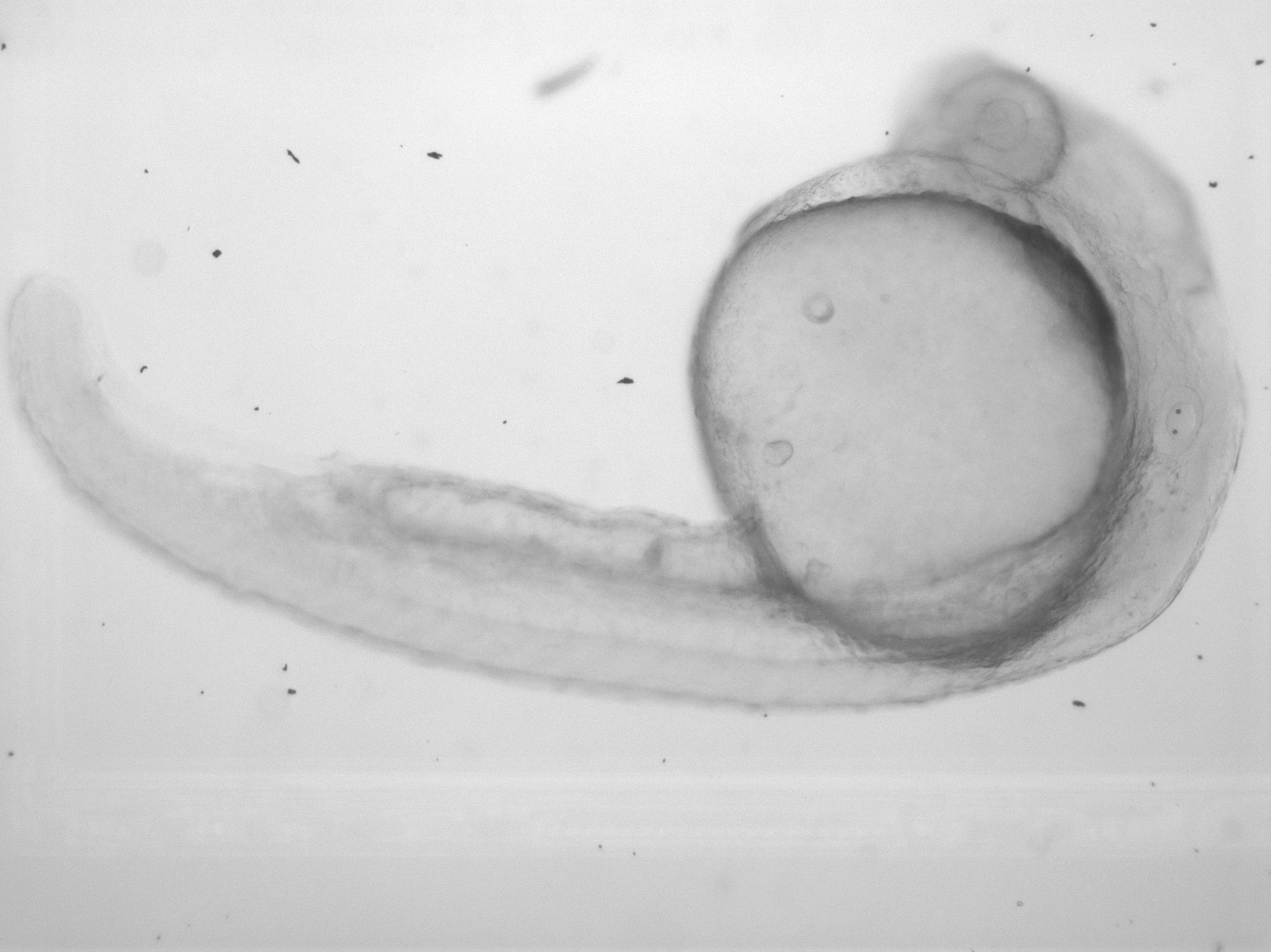

Supplement: Data S1 [file peerj-05-2894-s005.zip › Raw Data/24h-B.jpg]

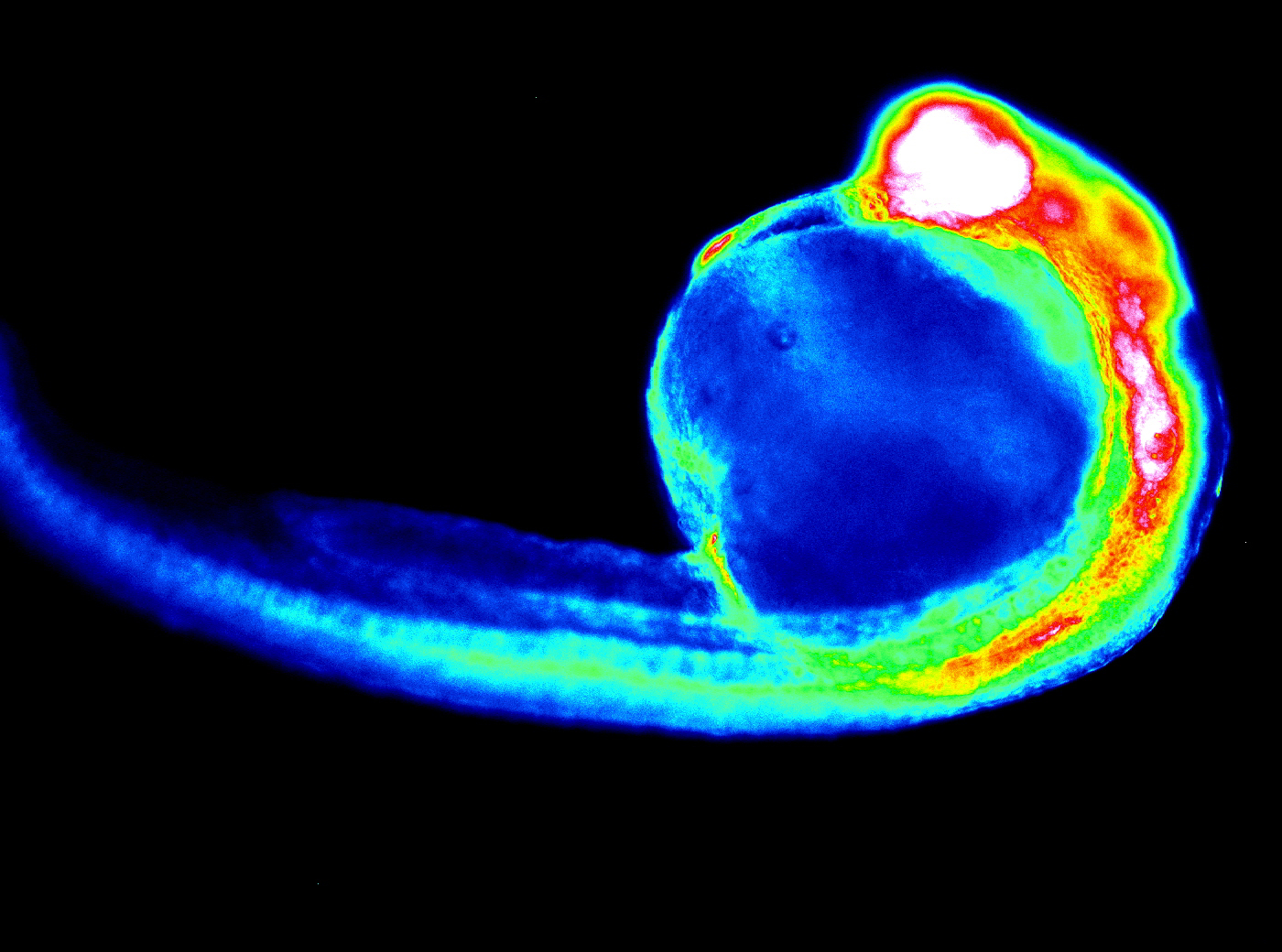

Supplement: Data S1 [file peerj-05-2894-s005.zip › Raw Data/24h-C.jpg]

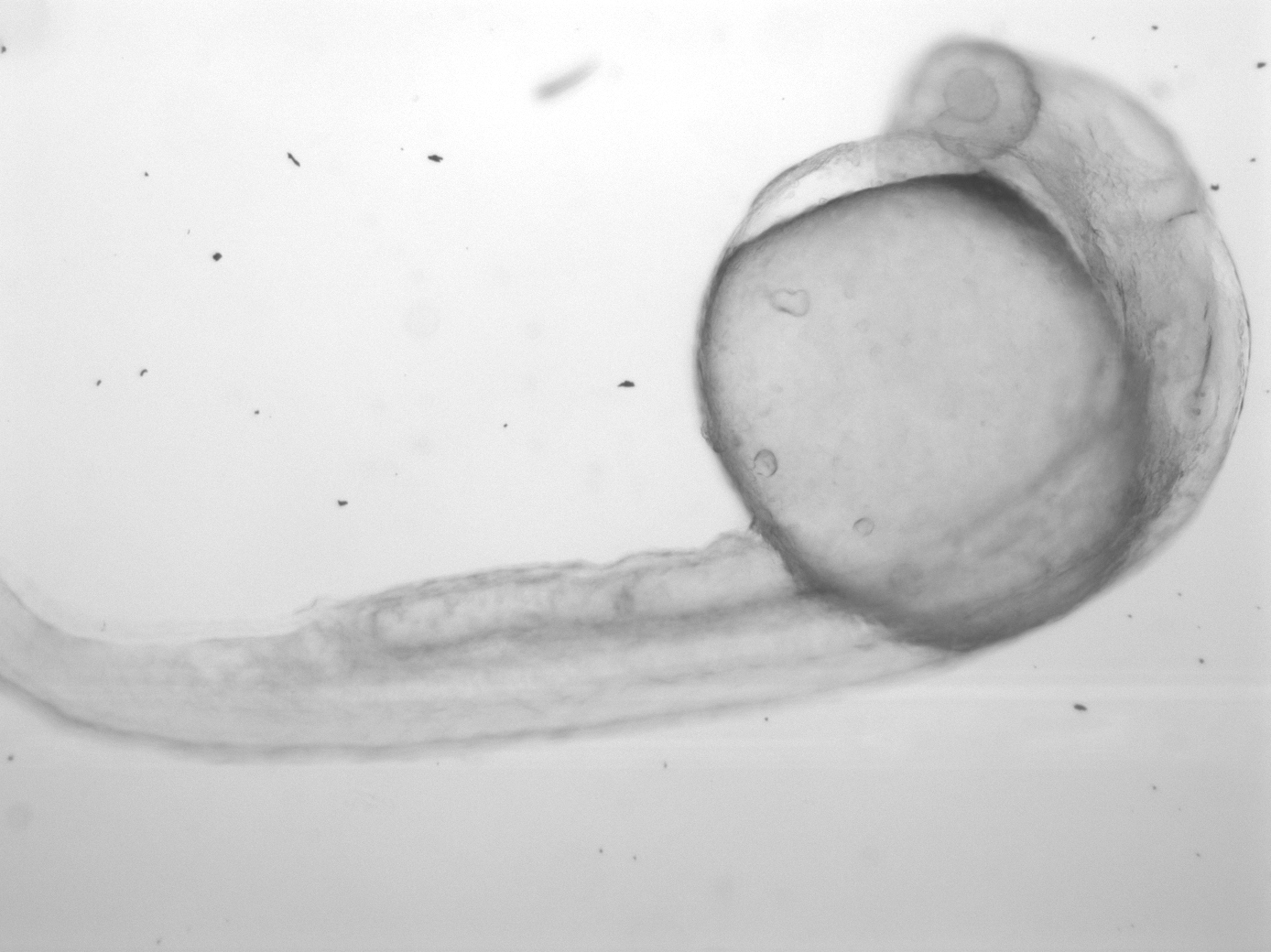

Supplement: Data S1 [file peerj-05-2894-s005.zip › Raw Data/25h-B.jpg]

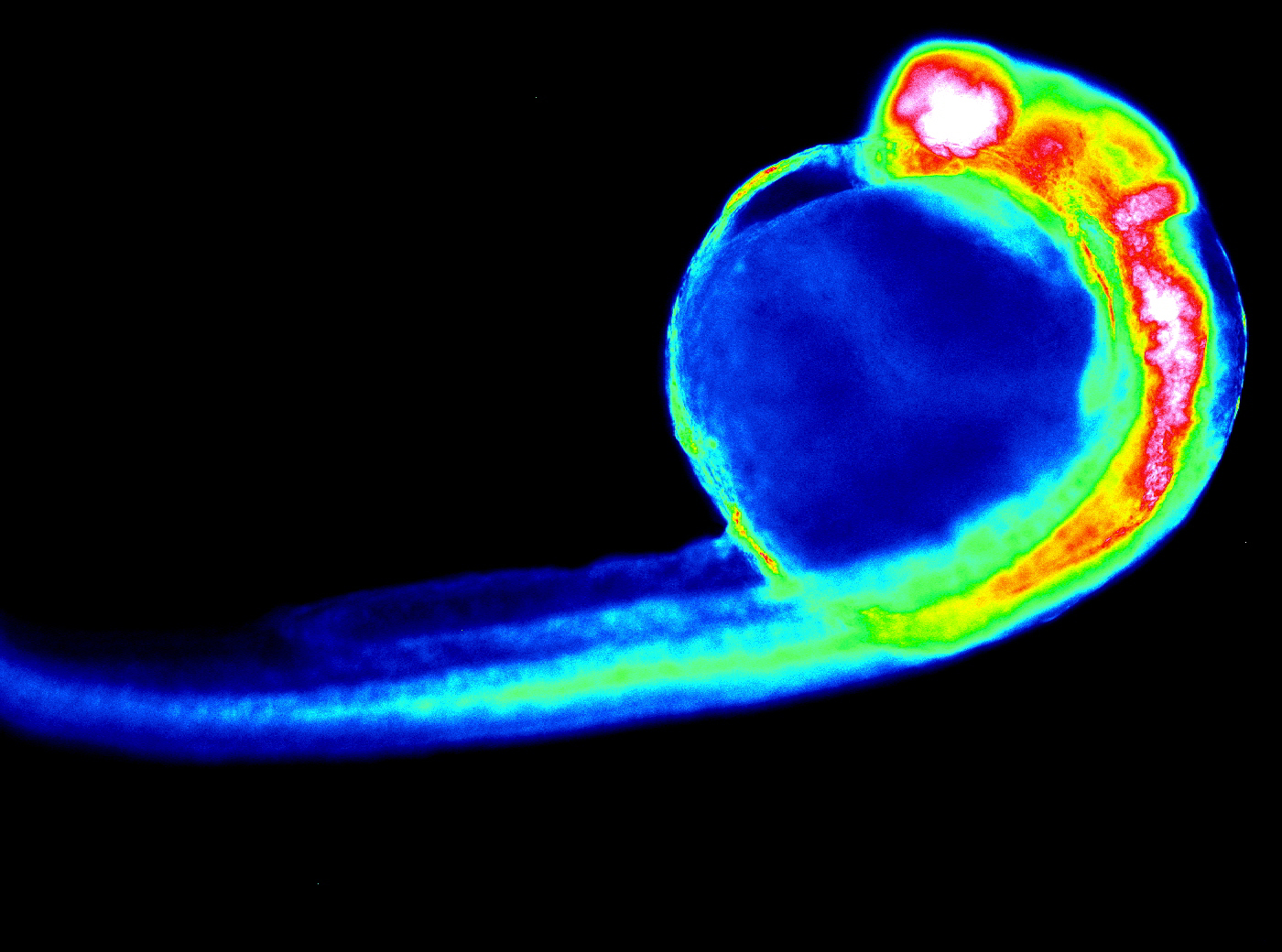

Supplement: Data S1 [file peerj-05-2894-s005.zip › Raw Data/25h-C.jpg]

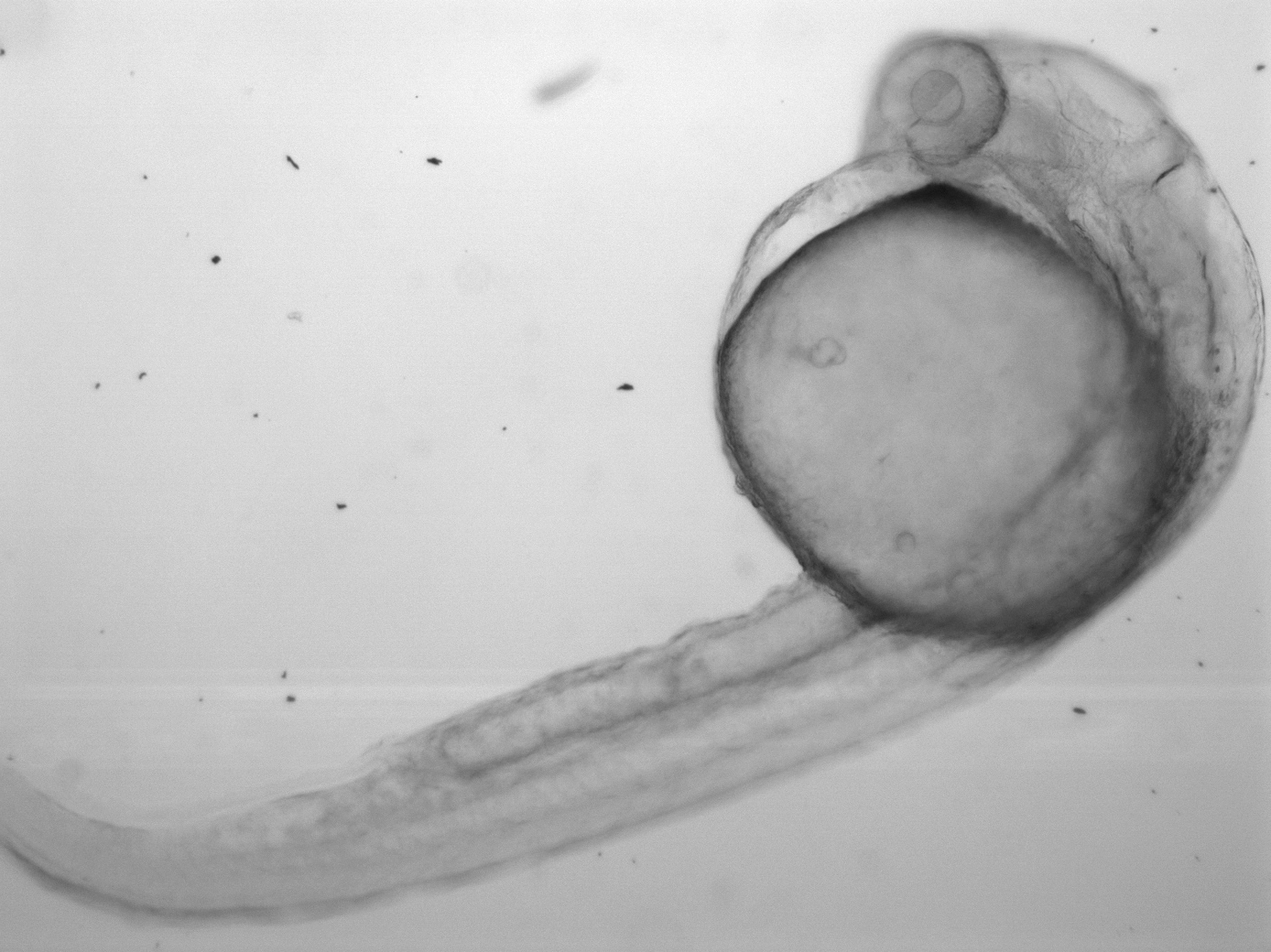

Supplement: Data S1 [file peerj-05-2894-s005.zip › Raw Data/26h-B.jpg]

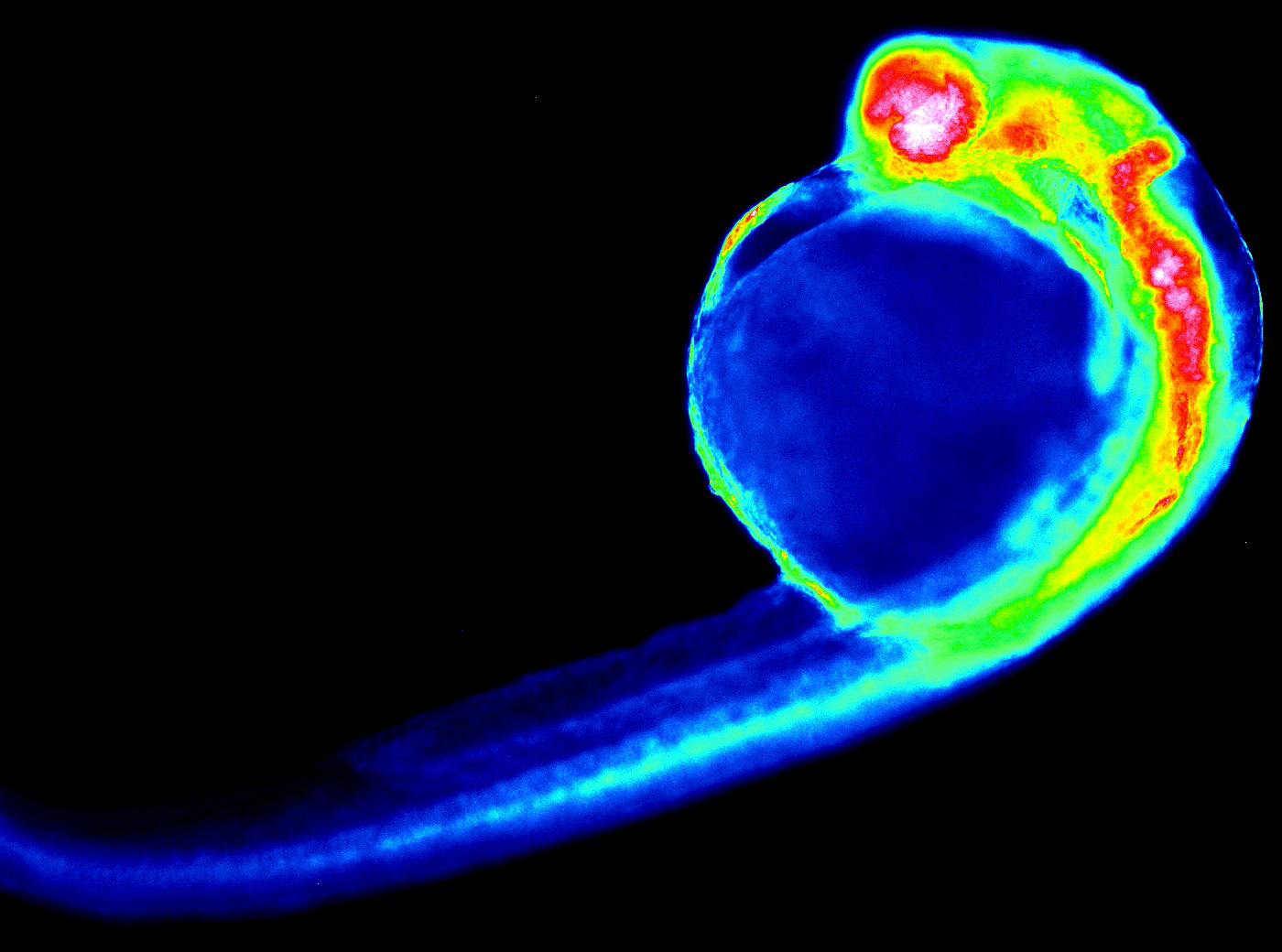

Supplement: Data S1 [file peerj-05-2894-s005.zip › Raw Data/26h-C.jpg]

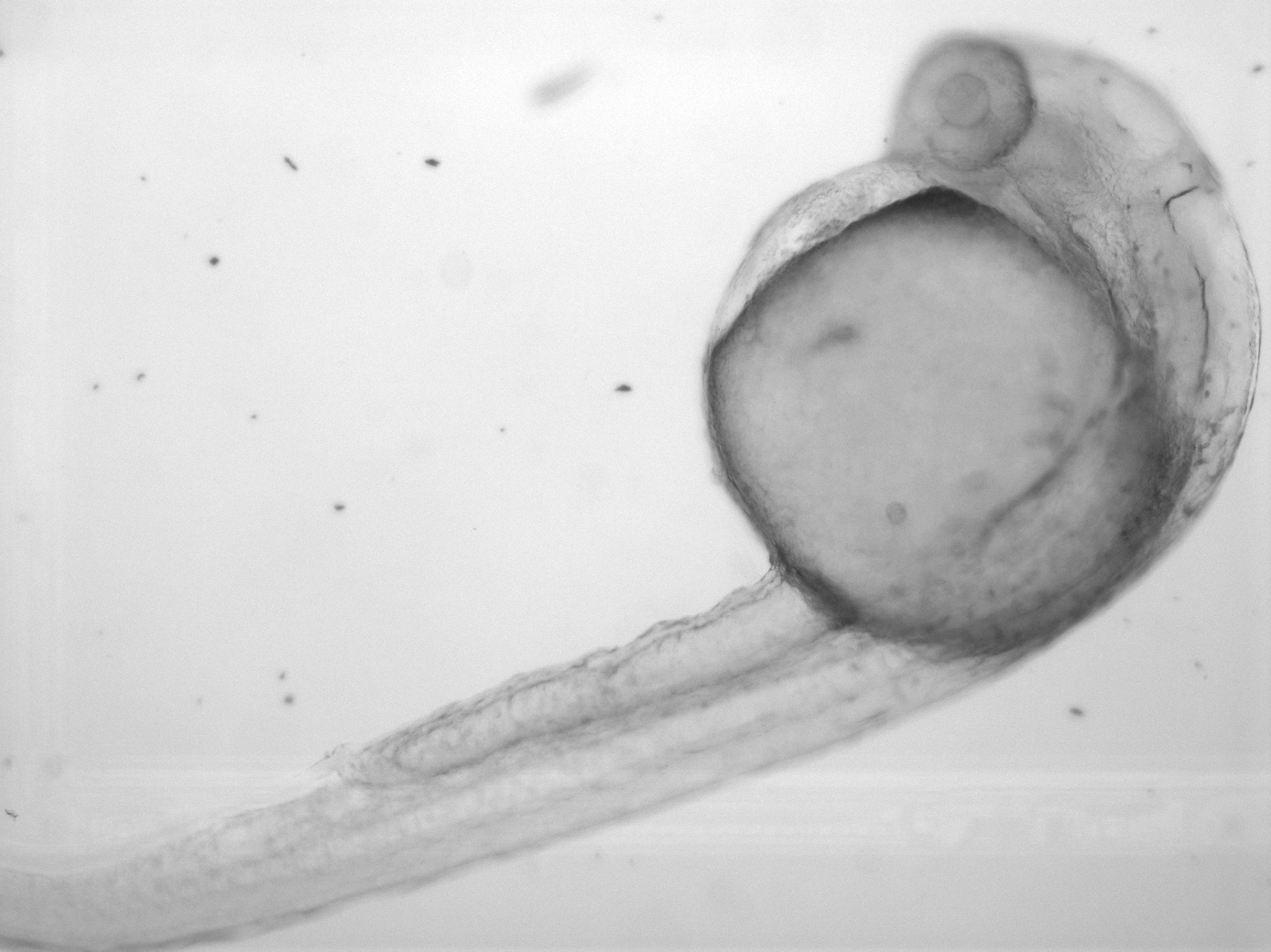

Supplement: Data S1 [file peerj-05-2894-s005.zip › Raw Data/27h-B.jpg]

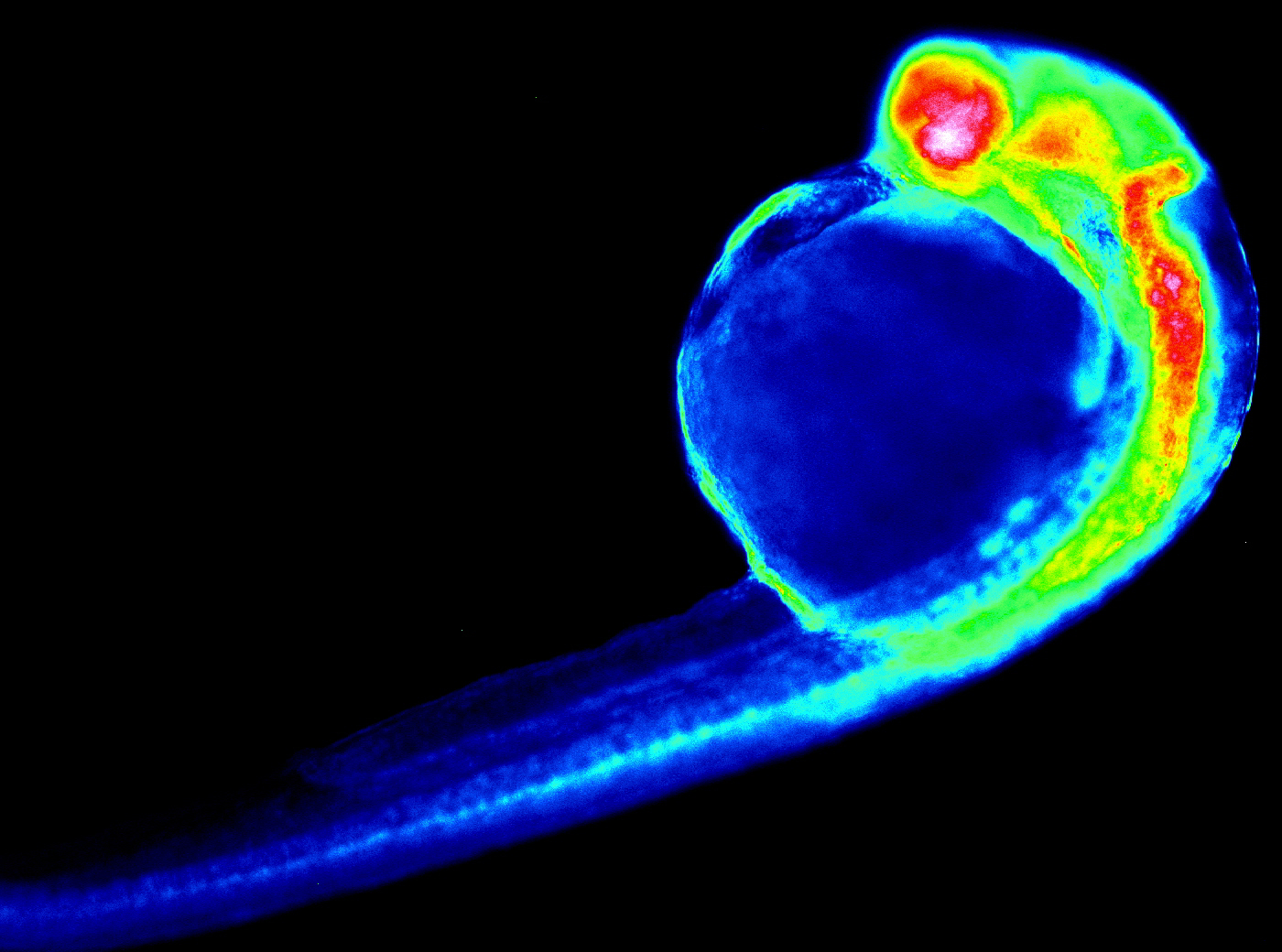

Supplement: Data S1 [file peerj-05-2894-s005.zip › Raw Data/27h-C.jpg]

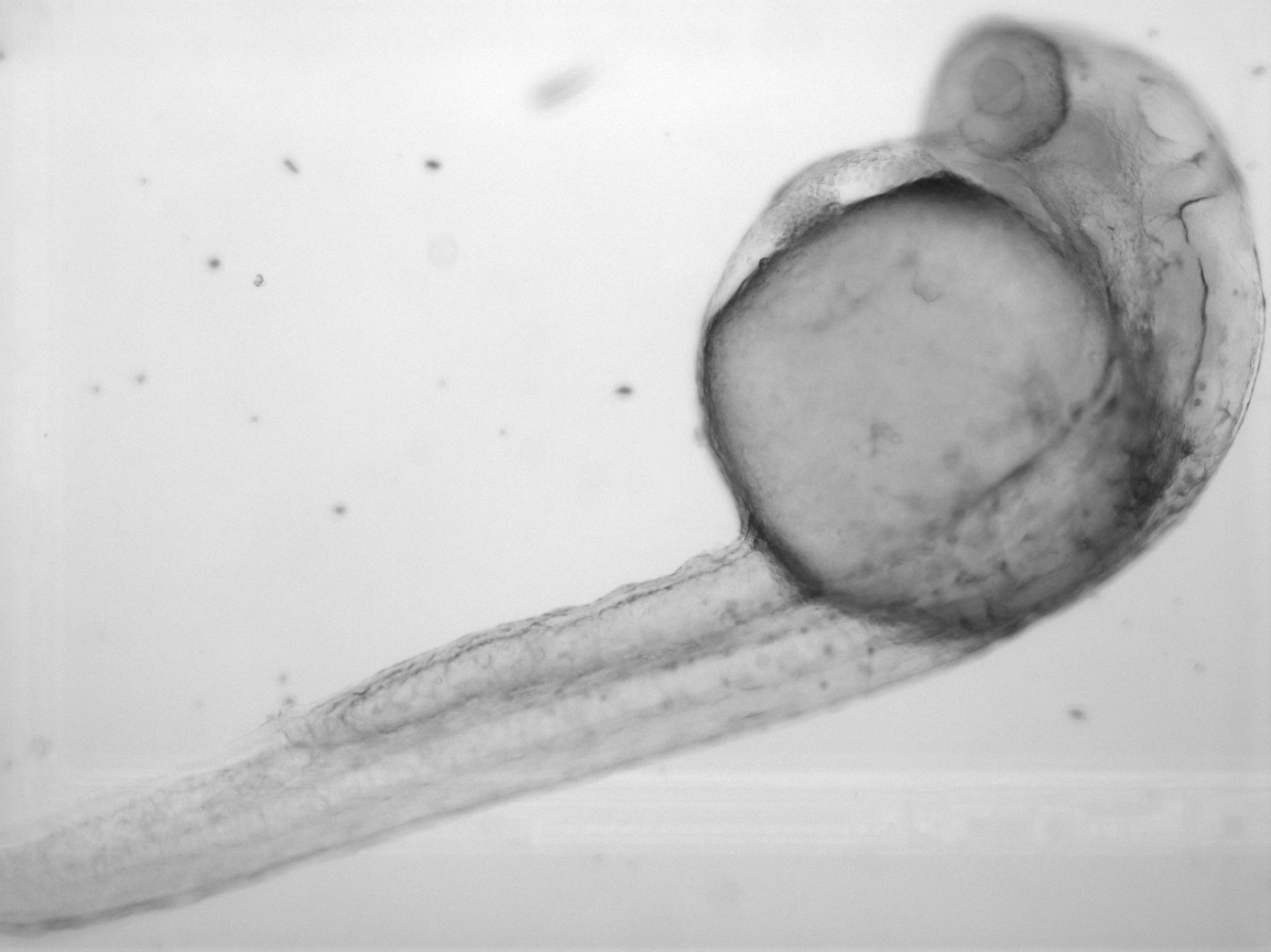

Supplement: Data S1 [file peerj-05-2894-s005.zip › Raw Data/28h-B.jpg]

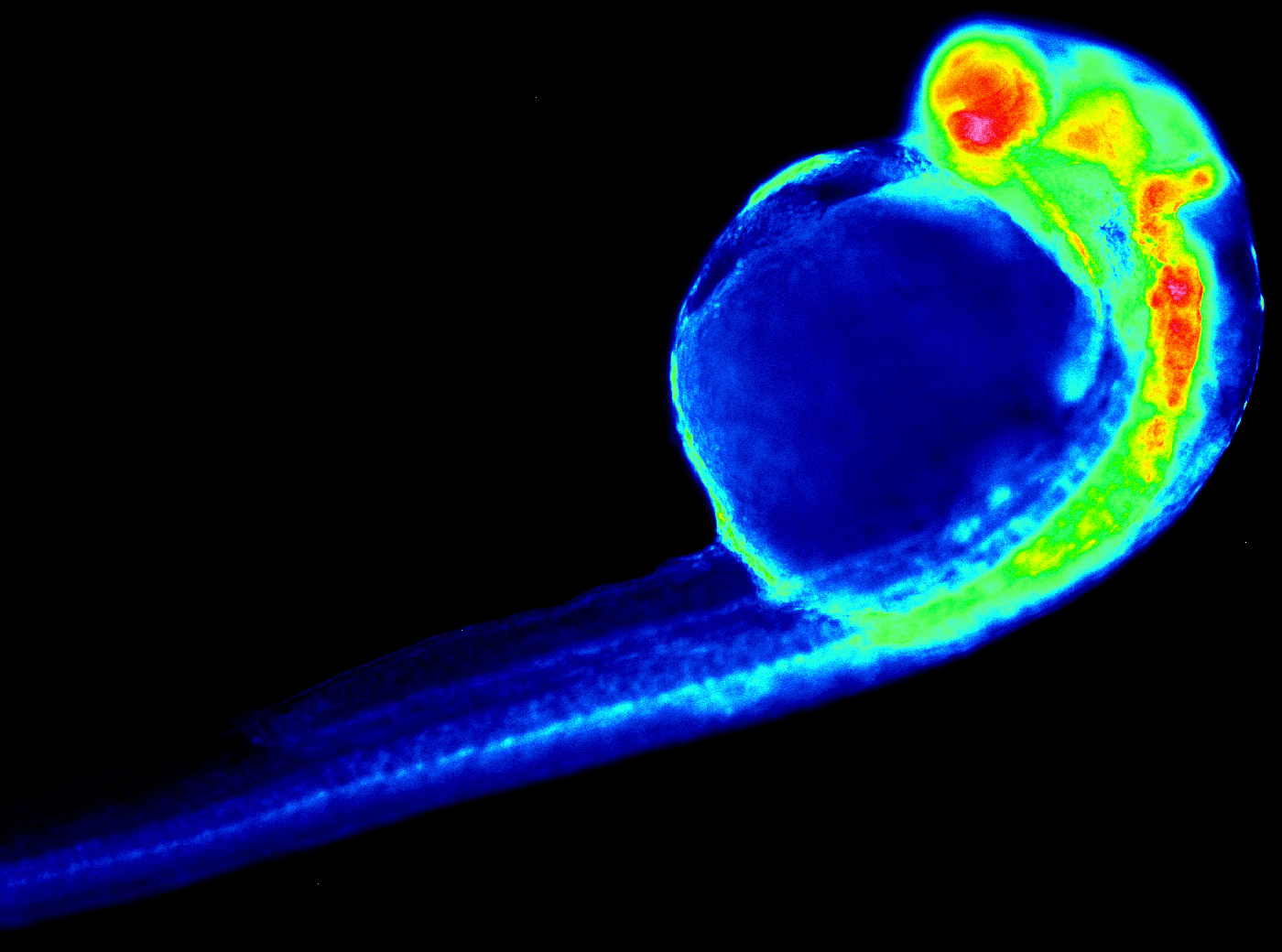

Supplement: Data S1 [file peerj-05-2894-s005.zip › Raw Data/28h-C.jpg]

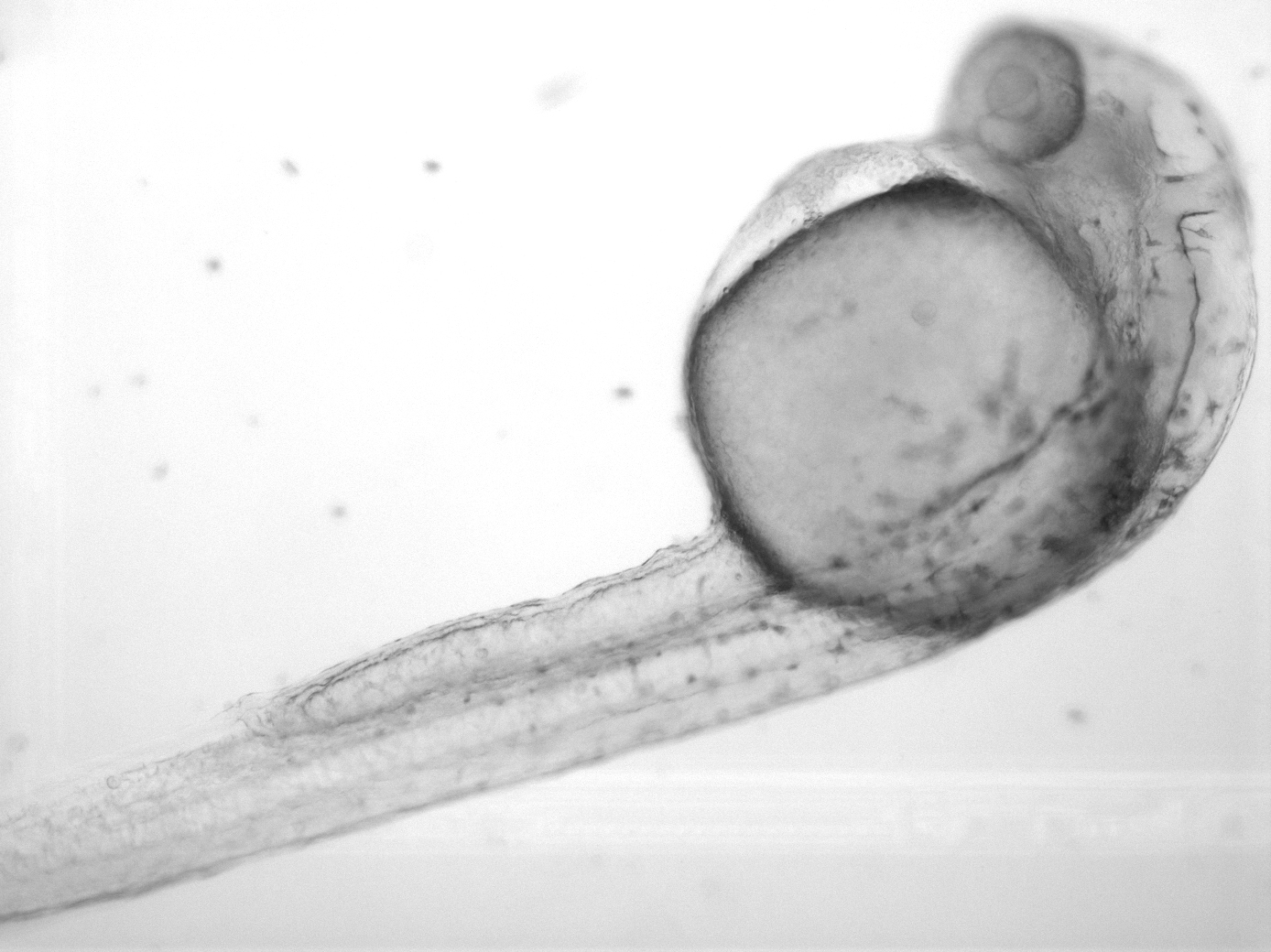

Supplement: Data S1 [file peerj-05-2894-s005.zip › Raw Data/29h-B.jpg]

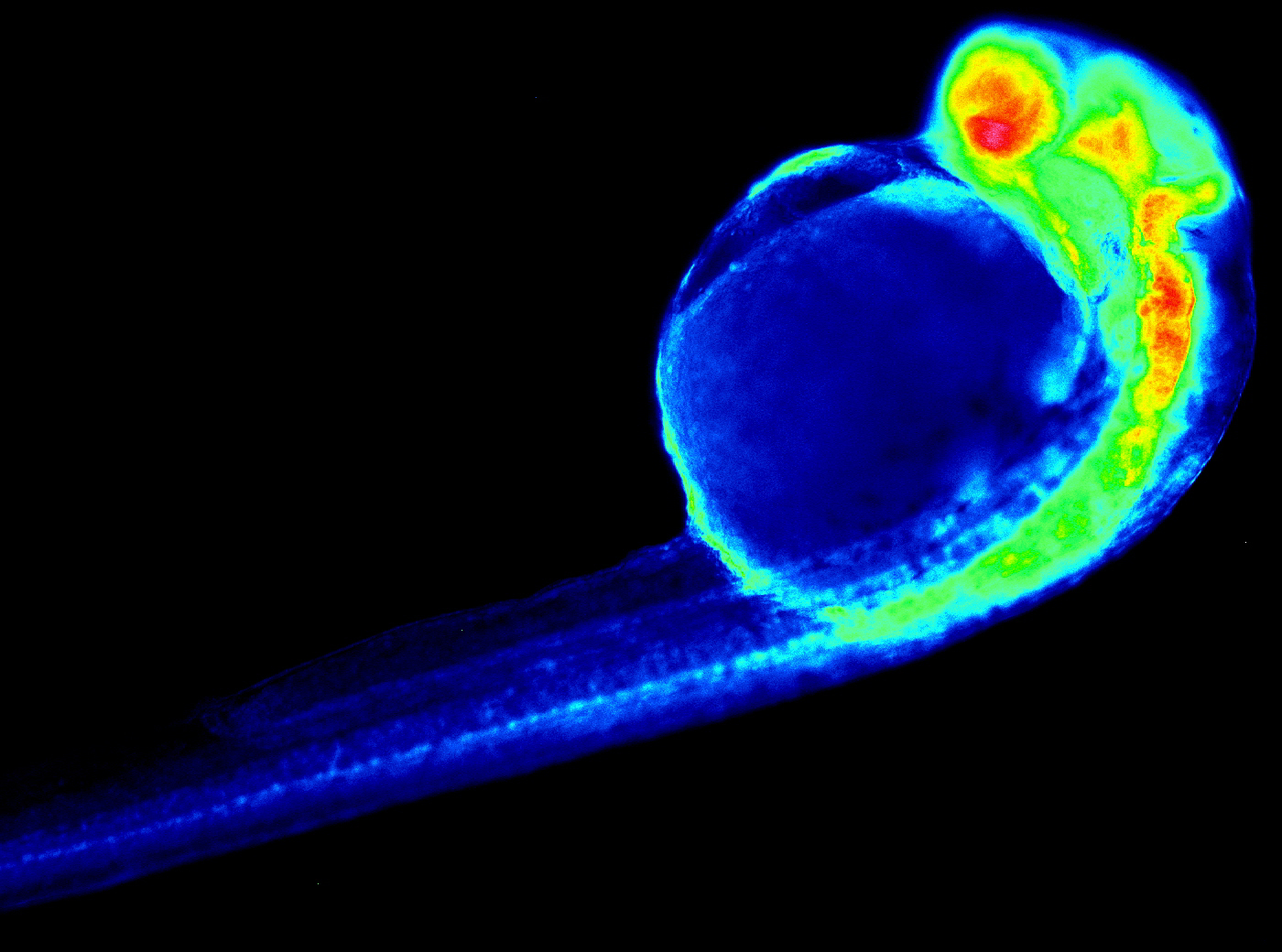

Supplement: Data S1 [file peerj-05-2894-s005.zip › Raw Data/29h-C.jpg]

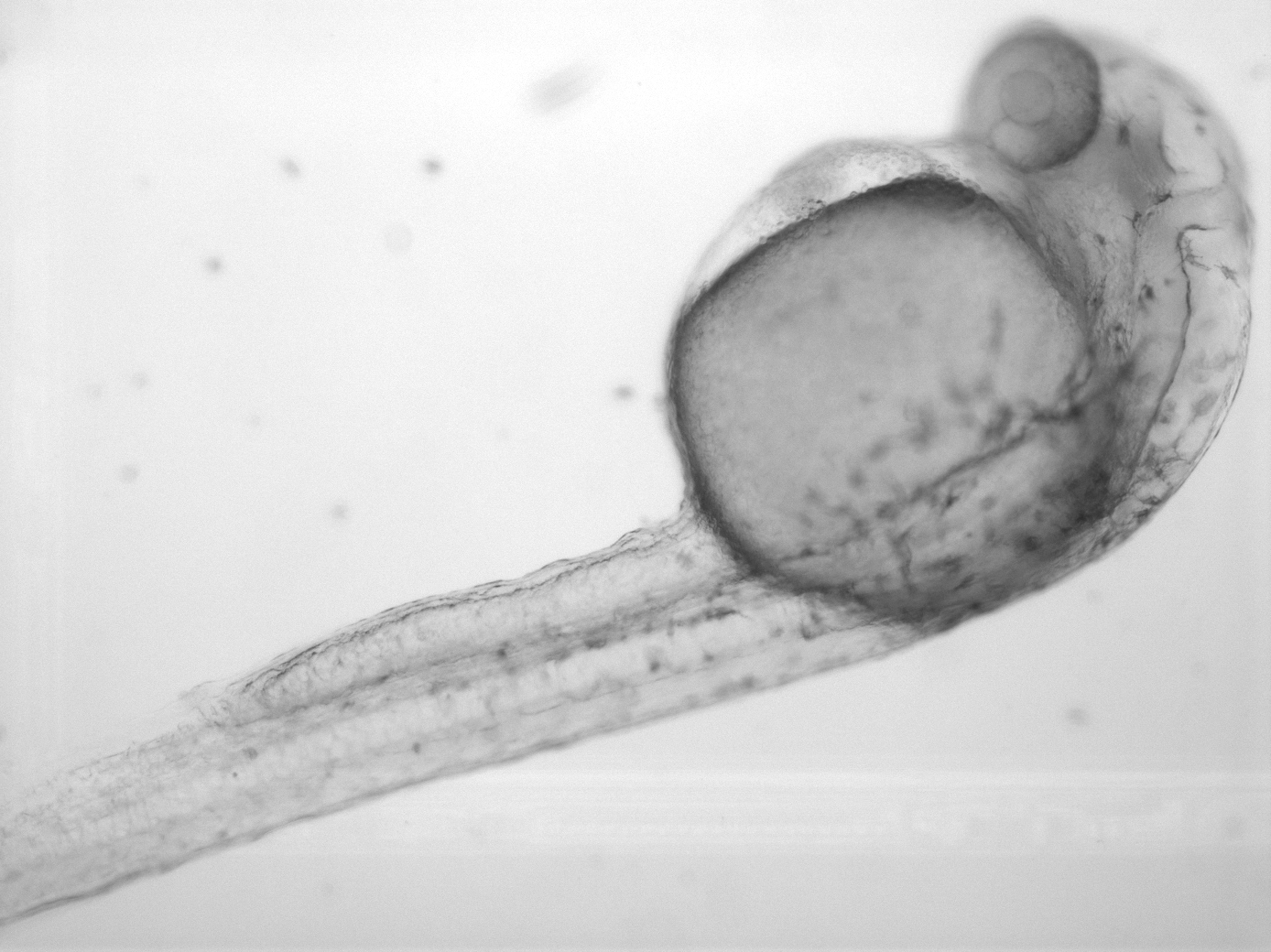

Supplement: Data S1 [file peerj-05-2894-s005.zip › Raw Data/30h-B.jpg]

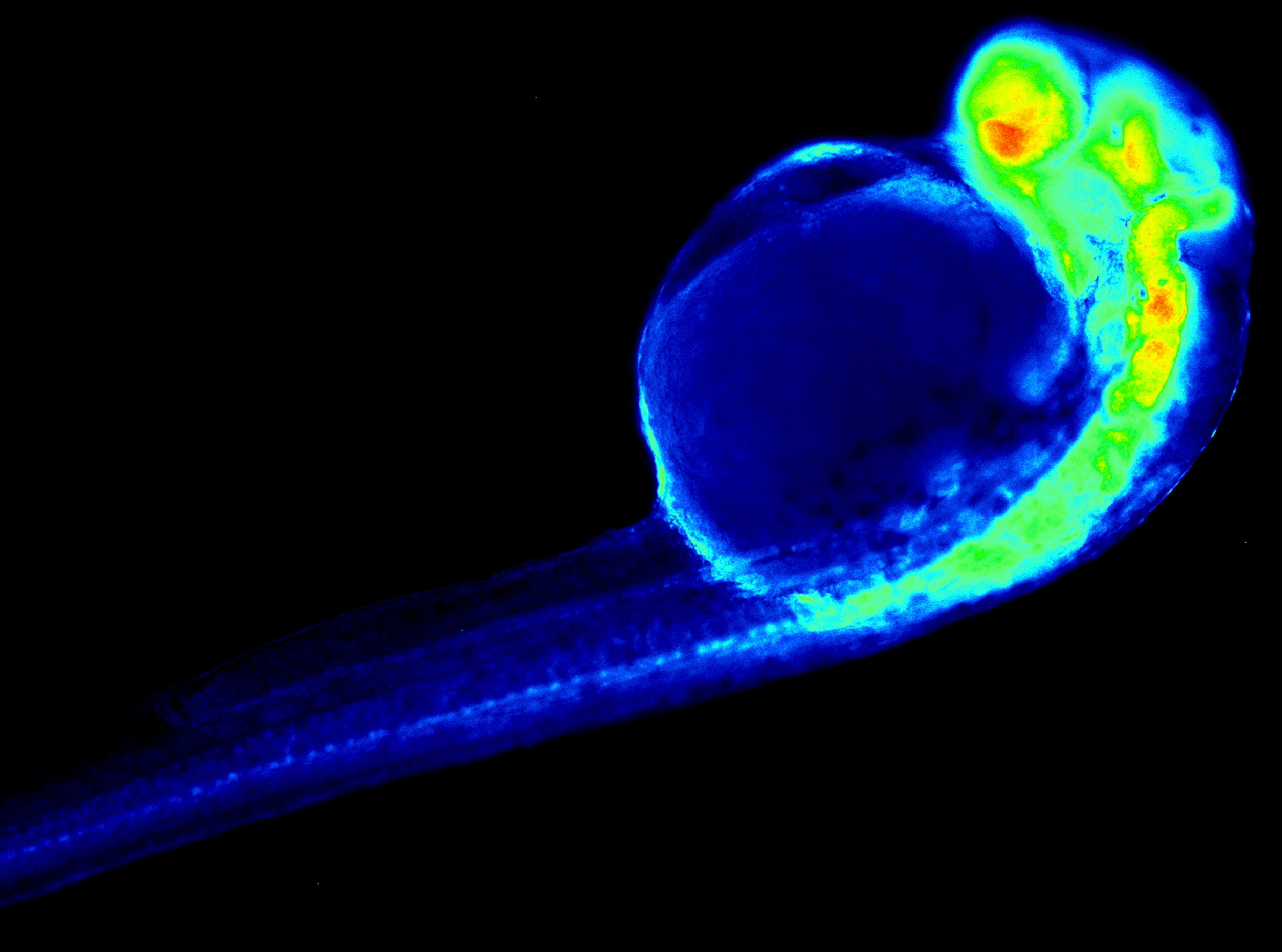

Supplement: Data S1 [file peerj-05-2894-s005.zip › Raw Data/30h-C.jpg]

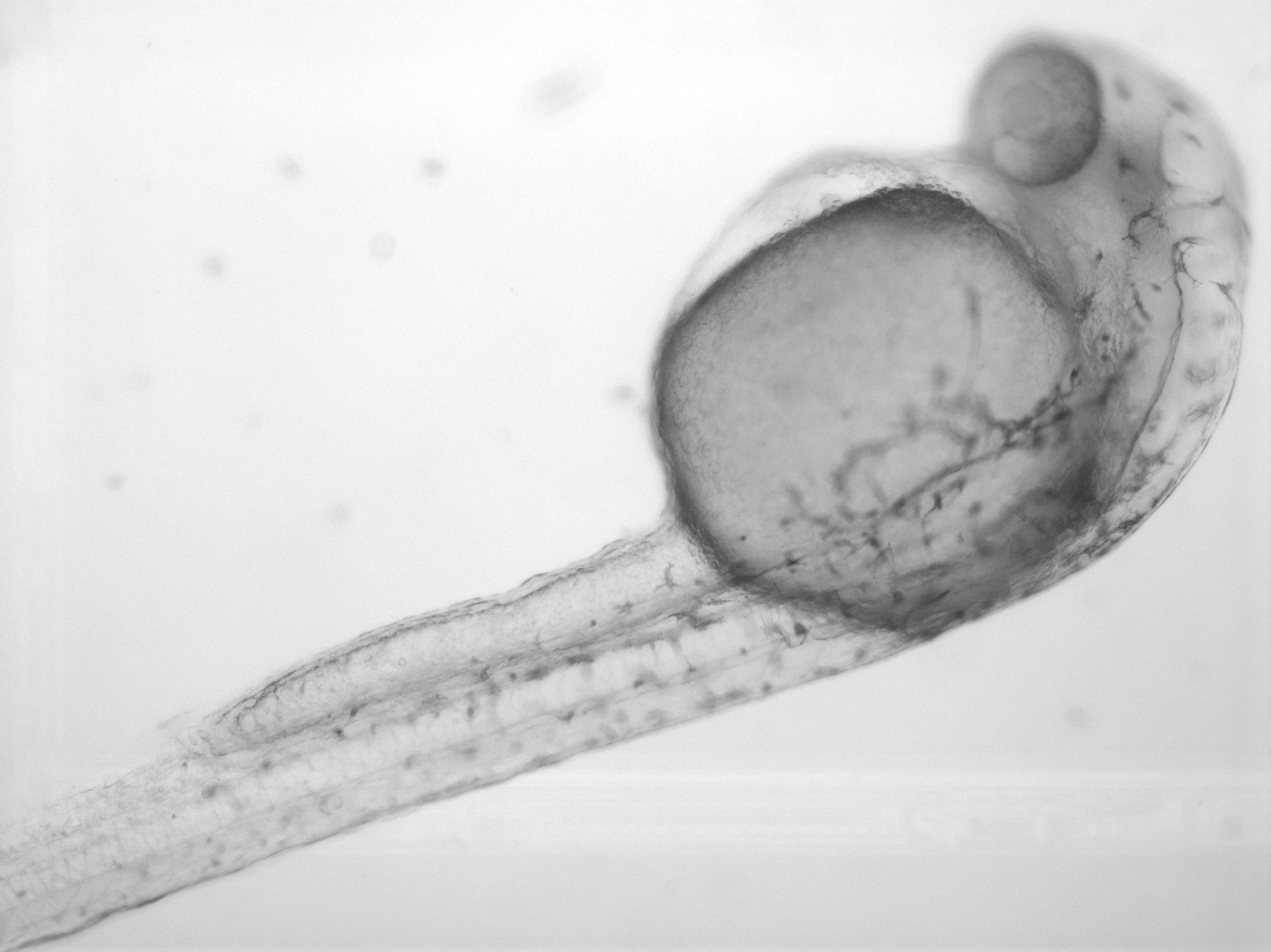

Supplement: Data S1 [file peerj-05-2894-s005.zip › Raw Data/31h-B.jpg]

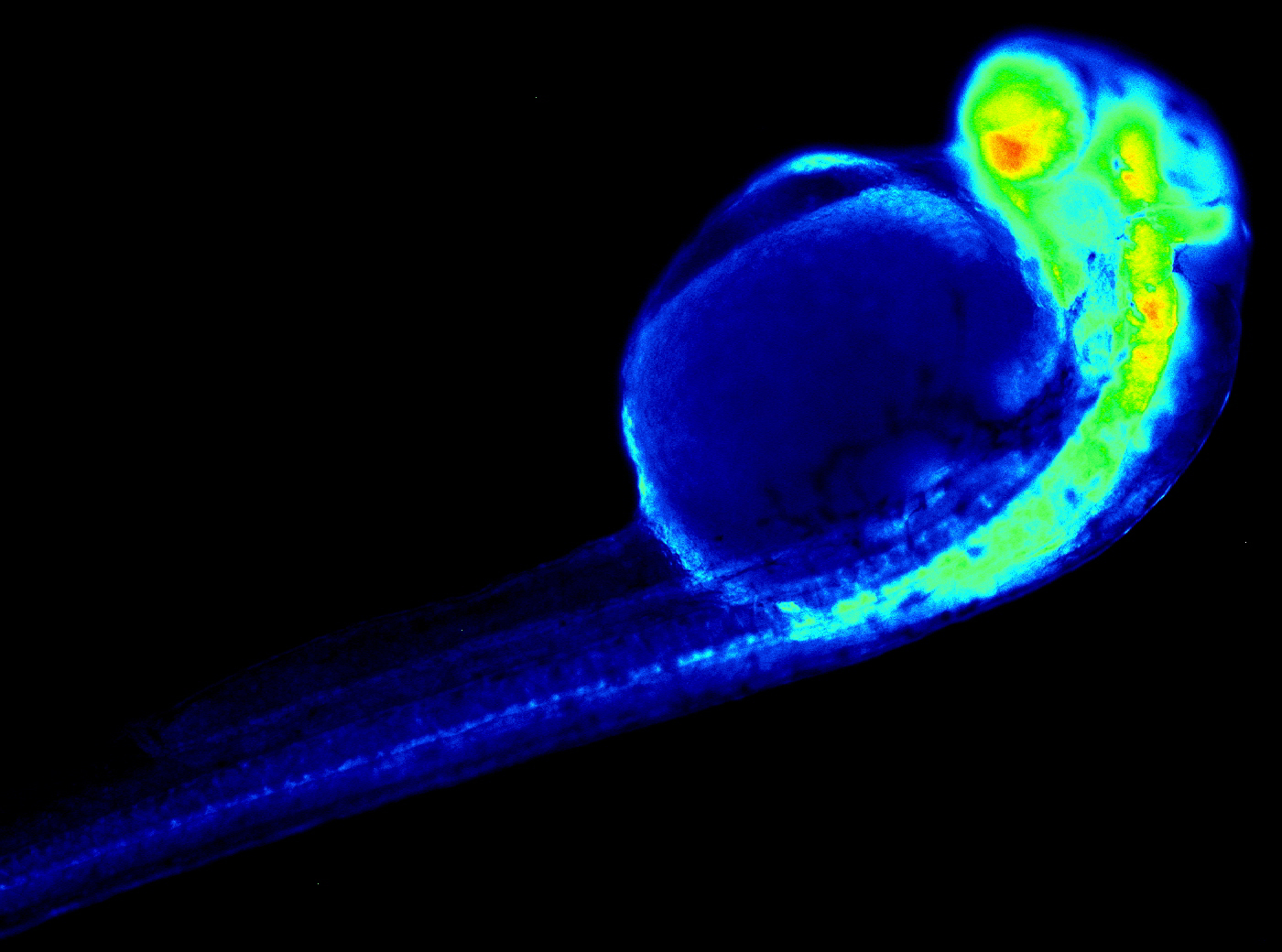

Supplement: Data S1 [file peerj-05-2894-s005.zip › Raw Data/31h-C.jpg]

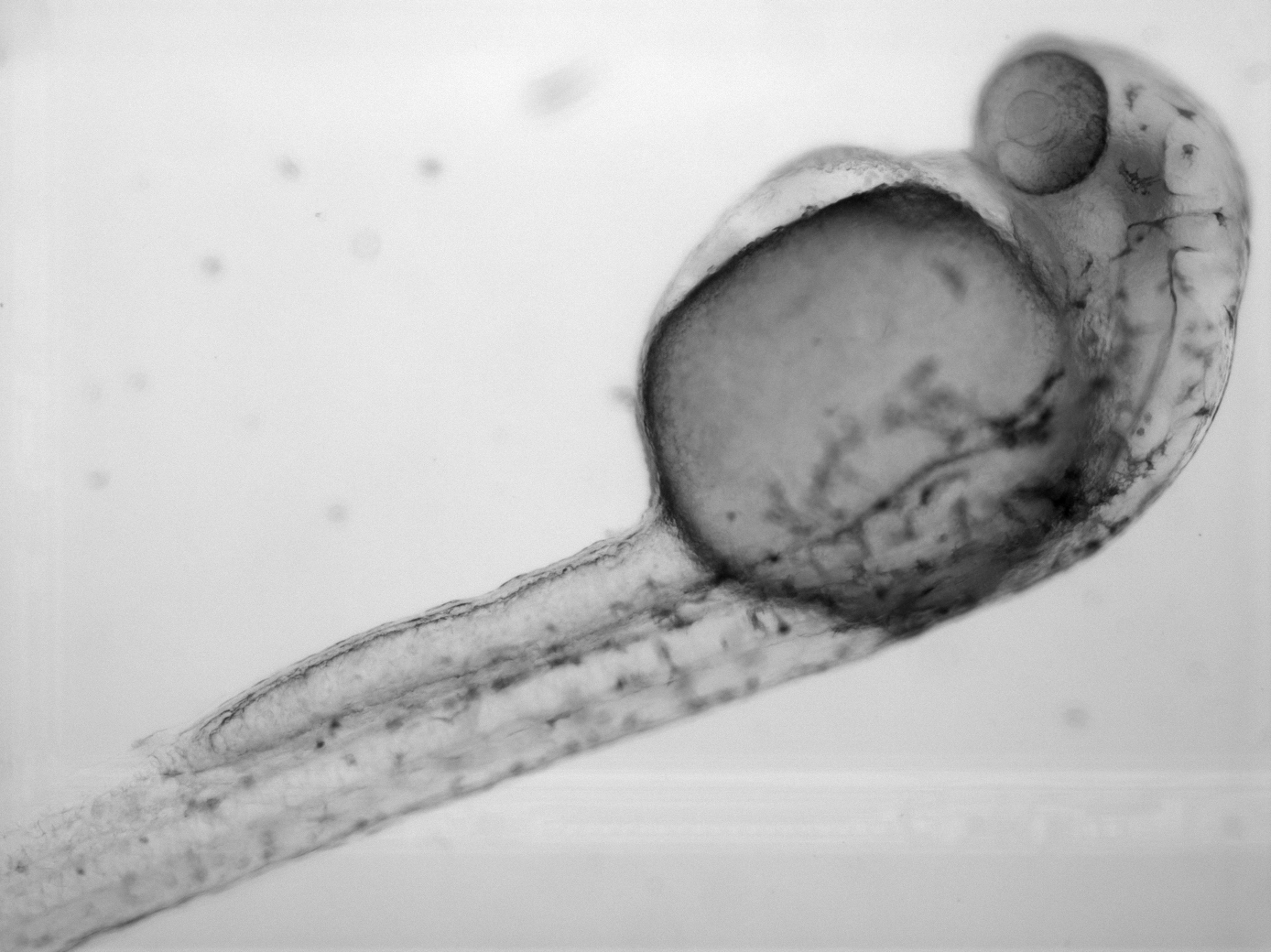

Supplement: Data S1 [file peerj-05-2894-s005.zip › Raw Data/32h-B.jpg]

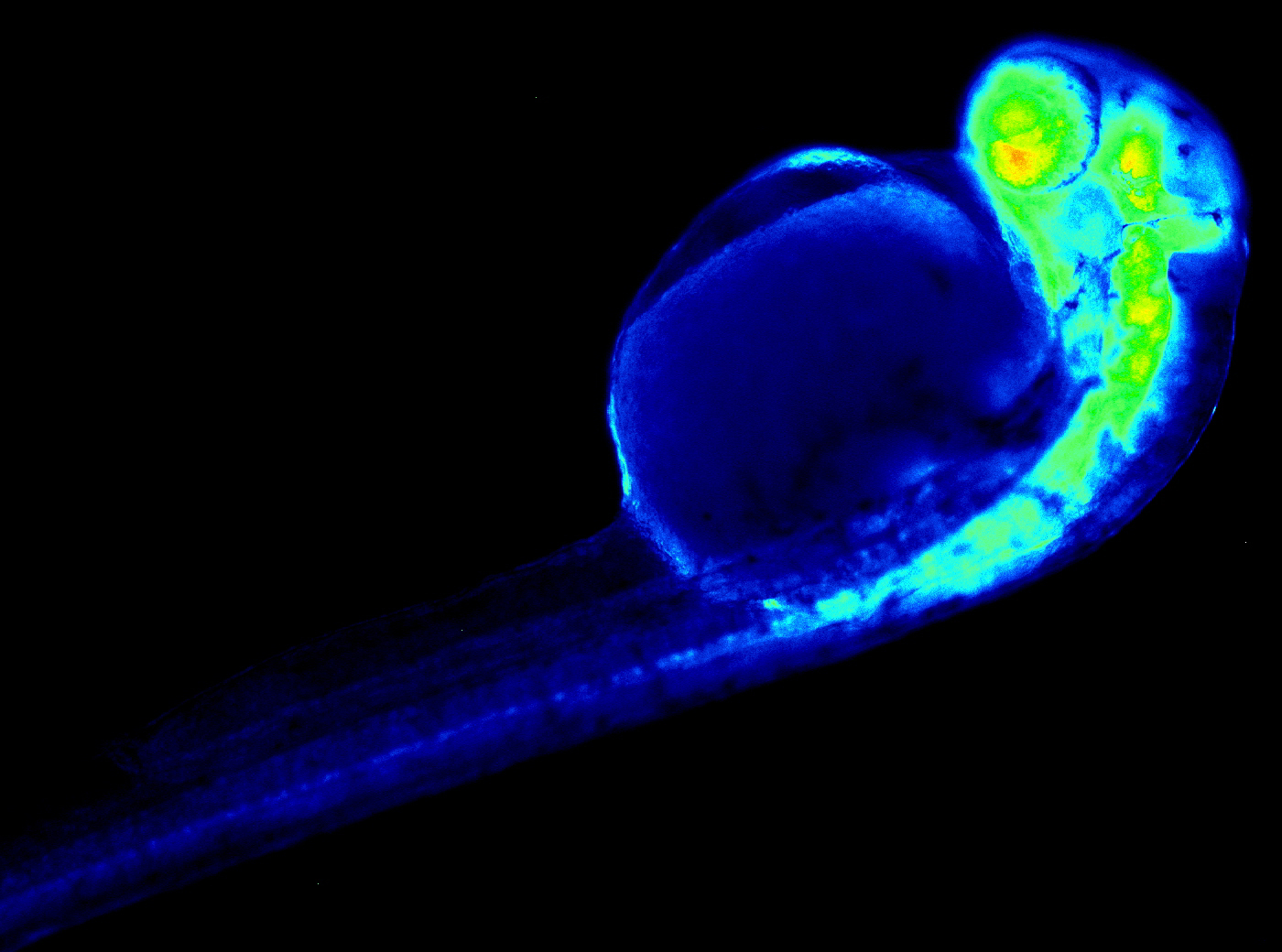

Supplement: Data S1 [file peerj-05-2894-s005.zip › Raw Data/32h-C.jpg]

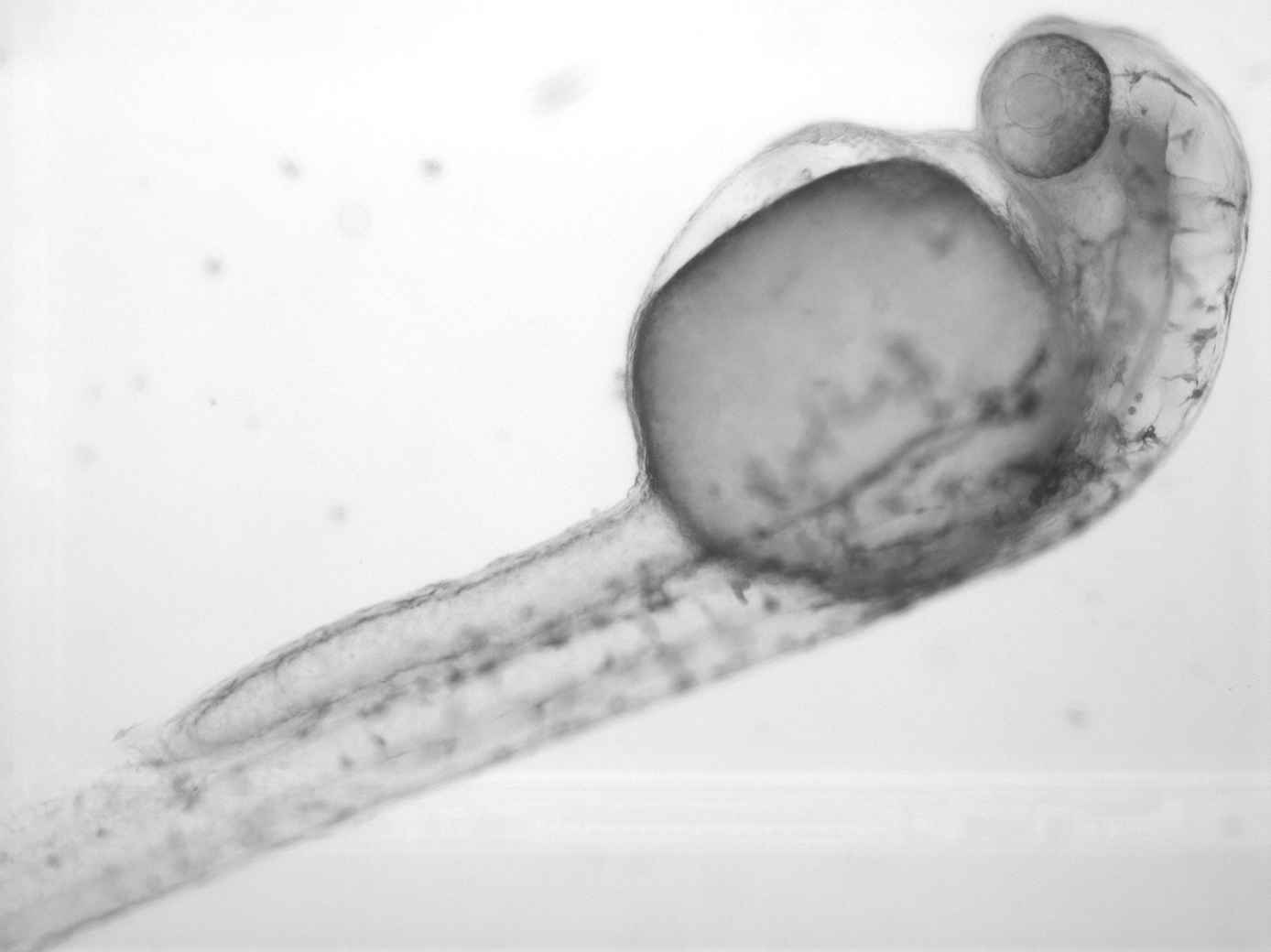

Supplement: Data S1 [file peerj-05-2894-s005.zip › Raw Data/33h-B.jpg]

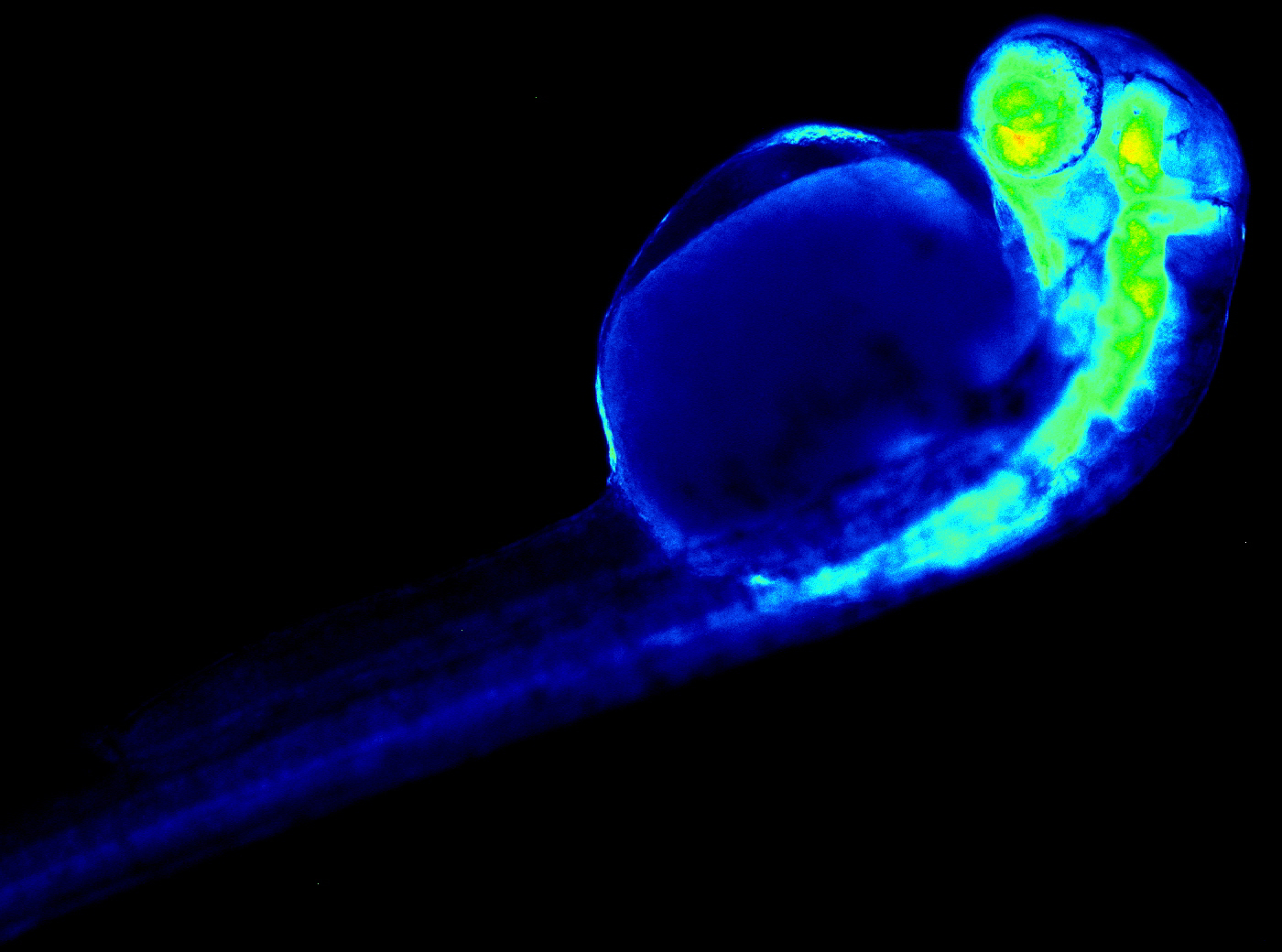

Supplement: Data S1 [file peerj-05-2894-s005.zip › Raw Data/33h-C.jpg]

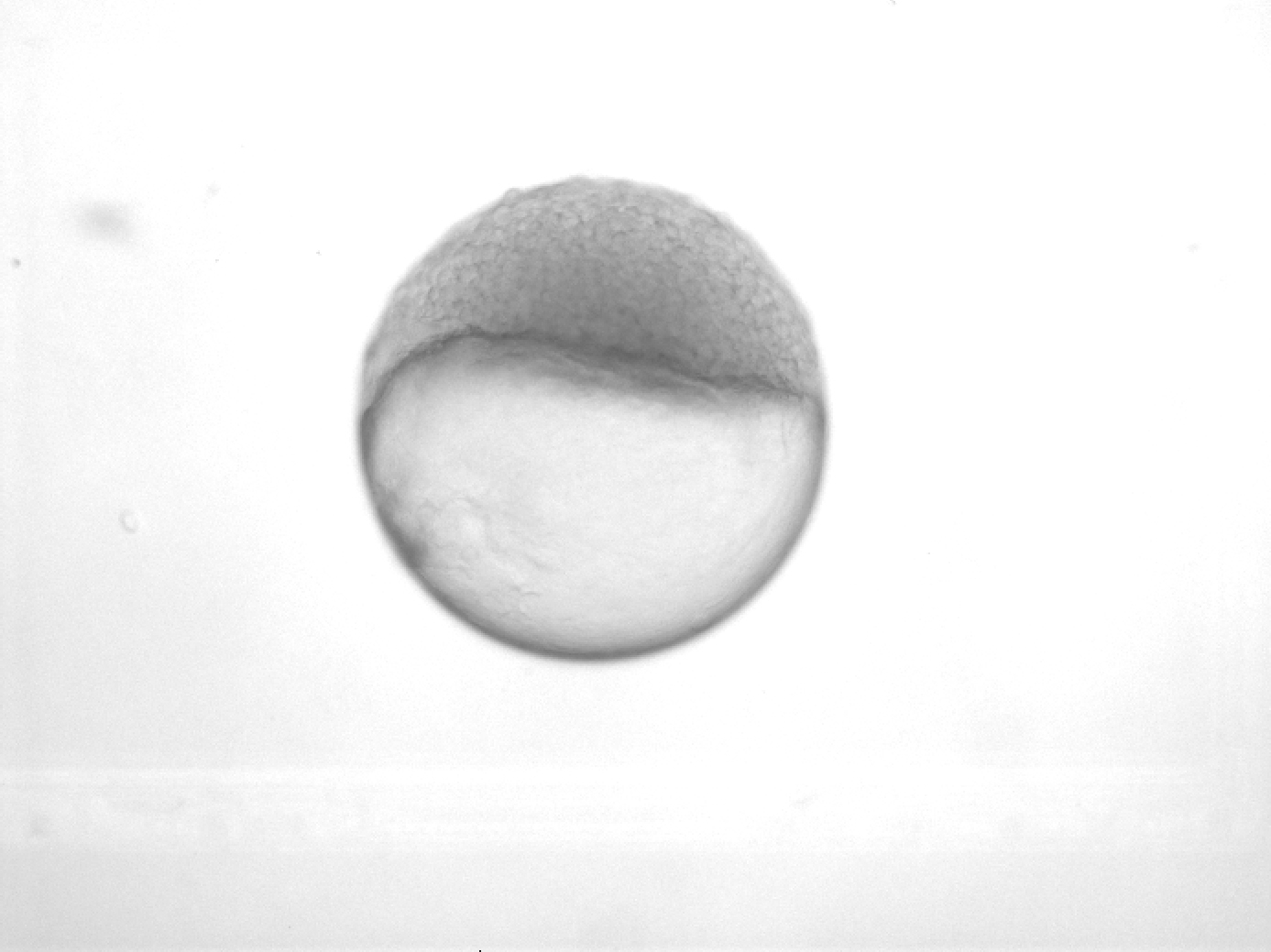

Supplement: Data S1 [file peerj-05-2894-s005.zip › Raw Data/S1_Thap-B.jpg]

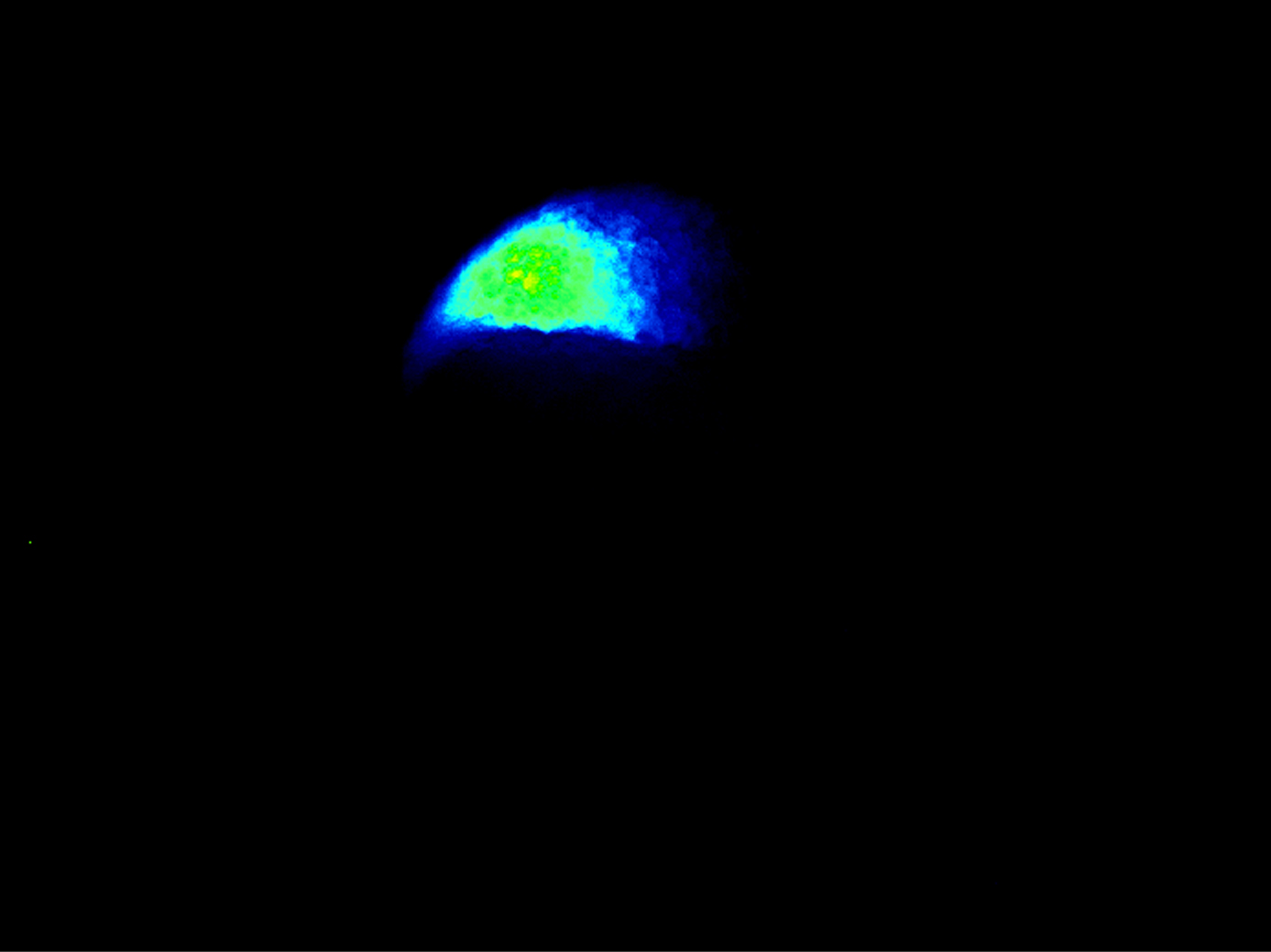

Supplement: Data S1 [file peerj-05-2894-s005.zip › Raw Data/S1_Thap-C.jpg]

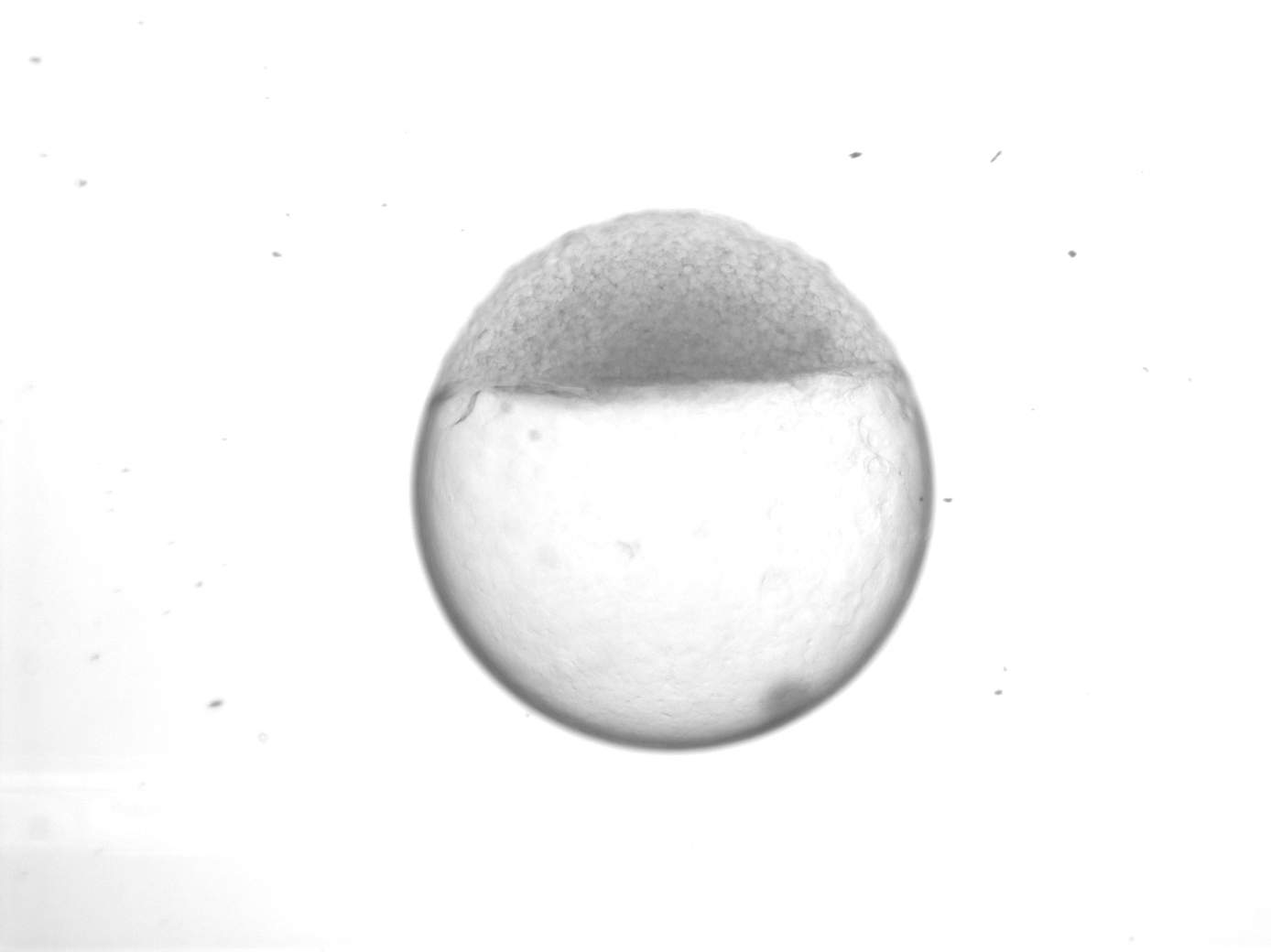

Supplement: Data S1 [file peerj-05-2894-s005.zip › Raw Data/S1_YC2-B.jpg]

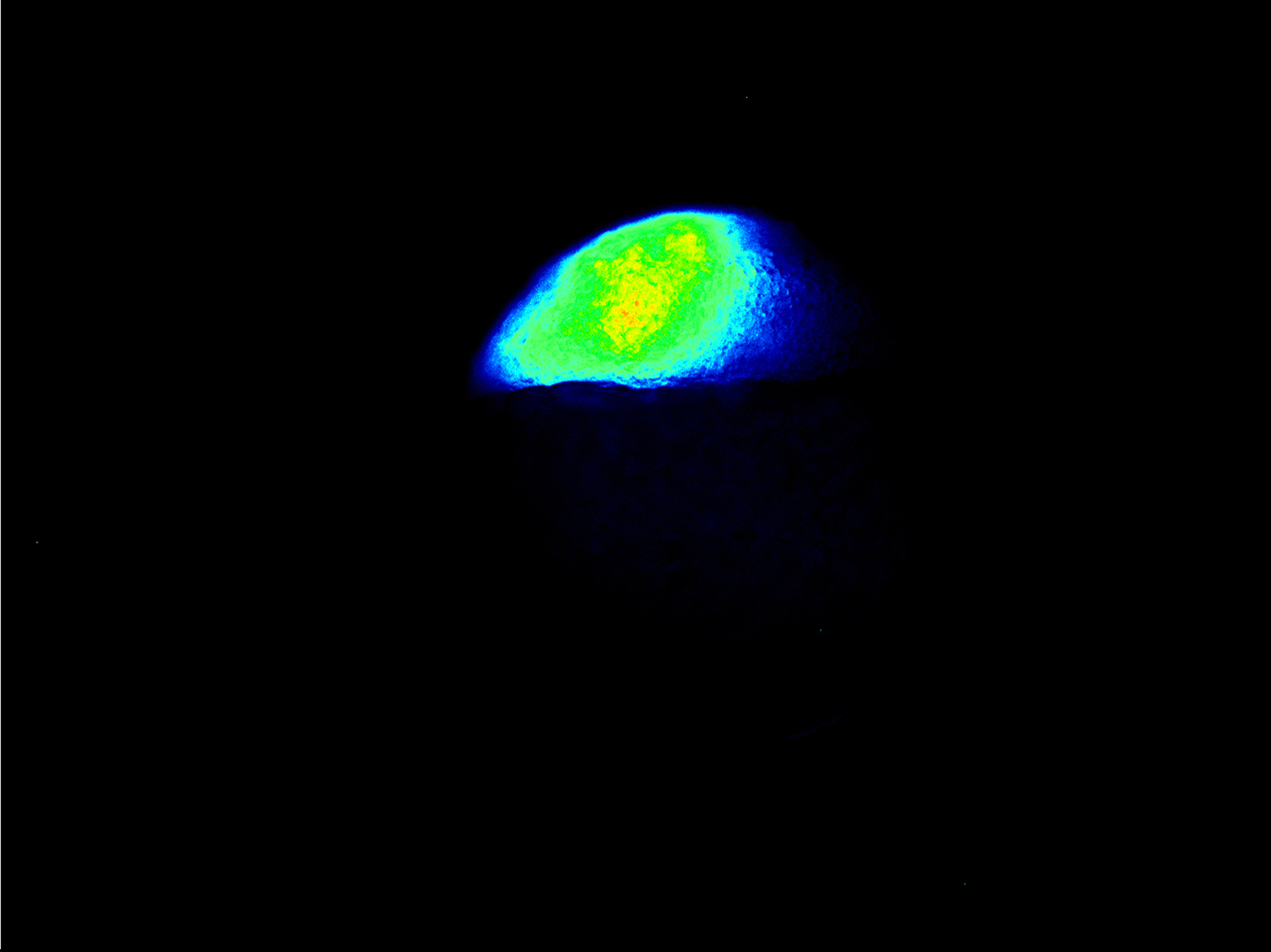

Supplement: Data S1 [file peerj-05-2894-s005.zip › Raw Data/S1_YC2-C.jpg]
